# An Illumina approach to MHC typing of Atlantic salmon

**DOI:** 10.1007/s00251-019-01143-8

**Published:** 2019-11-12

**Authors:** Arvind Y. M. Sundaram, Åse Helen Garseth, Giuseppe Maccari, Unni Grimholt

**Affiliations:** 1 Norwegian Veterinary Institute, P.O. Box 750 Sentrum, 0106 Oslo, Norway; 2 Department of Medical Genetics, Oslo University Hospital, 0450 Oslo, Norway; 3 The Pirbright Institute, Woking, UK; 4 Anthony Nolan Research Institute, London, UK

**Keywords:** MHC, Illumina, IPD-MHC database, Nomenclature, Salmonid, Atlantic salmon

## Abstract

The IPD-MHC Database represents the official repository for non-human major histocompatibility complex (MHC) sequences, overseen and supported by the Comparative MHC Nomenclature Committee, providing access to curated MHC data and associated analysis tools. IPD-MHC gathers allelic MHC class I and class II sequences from classical and non-classical MHC loci from various non-human animals including pets, farmed and experimental model animals. So far, Atlantic salmon and rainbow trout are the only teleost fish species with MHC class I and class II sequences present. For the remaining teleost or ray-finned species, data on alleles originating from given classical locus is scarce hampering their inclusion in the database. However, a fast expansion of sequenced genomes opens for identification of classical loci where high-throughput sequencing (HTS) will enable typing of allelic variants in a variety of new teleost or ray-finned species. HTS also opens for large-scale studies of salmonid MHC diversity challenging the current database nomenclature and analysis tools. Here we establish an Illumina approach to identify allelic MHC diversity in Atlantic salmon, using animals from an endangered wild population, and alter the salmonid MHC nomenclature to accommodate the expected sequence expansions.

**Electronic supplementary material:**

The online version of this article (10.1007/s00251-019-01143-8) contains supplementary material, which is available to authorized users.

## Introduction

Major histocompatibility complex (MHC) molecules have attracted a lot of attention due to their central role in discriminating between self and non-self and their enormous polymorphism. Classical MHC class I molecules are present on most cell types and present peptide fragments from self- and non-self-proteins to CD8+ T cells, thus initiating a protective immune response when presenting peptides from foreign proteins (Klein 1986). Classical MHC class II molecules are present on specialised antigen presenting cells and stimulate CD4+ T cells when presenting peptides originating from foreign endocytosed proteins.

The IPD-MHC Database contains sequence data from classical and non-classical MHC class I and class II genes from non-human species such as important farmed animals, experimental animals or pets (Maccari et al. 2017). To be defined as a classical locus, one needs to know that the gene is highly polymorphic, the alleles must be peptide binders and the molecules must be membrane proteins. Classical MHC class I molecules are expressed on most cells so the expression patterns must comply with this expectation. For class II, the classical molecules are only expressed on specialised antigen presenting cells, influencing the expected transcriptional patterns. For species without a clear-cut understanding of the number of genes and their genomic organization, it is more difficult to link a nucleotide sequence to a specific locus. Thus, the species included in the IPD-MHC Database have well-defined number of classical genes and can link included nucleotide sequences to a given locus. In tetrapod’s, the MHC class I and class II genes are physically linked in one genomic region, but in teleost fishes, there is no major histocompatibility complex in teleost fishes as the classical class I and II loci identified reside on different chromosomes (Bingulac-Popovic et al. 1997).

Currently, only the salmonids Atlantic salmon *Salmo salar* and rainbow trout *Oncorhynchus mykiss* MHC represent ray-finned and teleost species in the IPD-MHC fish section. Due to their economic importance in aquaculture, identifying the classical MHC loci and defining their alleles, peptide binding ability and transcriptional patterns have been a priority (Grimholt et al. 2015; Kiryu et al. 2005; Lukacs et al. 2007; Shiina et al. 2005).

Genomes of a large number of other salmonid species are becoming available (e.g. Chinook salmon *Oncorhynchus tshawytscha* (Christensen et al. 2018a), Arctic char *Salvelinus alpinus* (Christensen et al. 2018b), grayling *Thymallus thymallus* (Savilammi et al. 2019)) making them prime candidates for inclusion in the IPD-MHC Database once MHC alleles are defined such as already initiated for brown trout *Salmo trutta* (O’Farrell et al. 2013).

Among other ray-finned or teleost species, medaka *Oryzias latipes* and zebrafish *Danio rerio* have the most reliable information regarding number of classical loci. Medaka has two defined classical MHCI loci denoted UAA and UBA (Nonaka and Nonaka 2010), but only one classical MHCII alpha and beta gene (Bannai and Nonaka 2013). Zebrafish has haplotypes with varying number of assumed classical MHCI genes (McConnell et al. 2014) while the number of classical MHCII genes is still undefined (Dijkstra et al. 2013; Ono et al. 1992; Sultmann et al. 1994; Sultmann et al. 1993). In other species, the number of classical MHC genes is mostly undefined with the exception of Atlantic cod *Gadus morhua* where the number of class I genes has greatly expanded while the MHCII genes, invariant chain and CD4 have been lost entirely (Star et al. 2011).

In salmonids, there are six characterised MHC class I lineages defined based on sequence identity denoted U, Z, S, L, P and H lineages (Dijkstra et al. 2007; Grimholt et al. 2015; Grimholt et al. 2019; Shum et al. 1999; Stet et al. 1998). Of these six lineages, only the U and Z lineages are peptide binders where the single classical locus UBA identified in rainbow trout and Atlantic salmon belongs to the U lineage (Grimholt et al. 2015). Other U lineage loci, i.e. the UCA, UDA, ULA, UGA and UHA, in these salmonids are defined as non-polymorphic where all but UGA have more restricted tissue distribution patterns. Also for MHC class II, there are multiple lineages denoted A, B and E lineages where the single classical MHC class II alpha (DAA) and class II beta (DAB) loci belong to the A lineage in Atlantic salmon and rainbow trout (Dijkstra et al. 2013; Grimholt 2016). There is a close physical linkage between these salmonid DAA and the DAB genes so their alleles segregate as a functional haplotype (Stet et al. 2002). The stability of these MHC class II haplotypes remains to be established.

For the salmonids Atlantic salmon and rainbow trout, the IPD-MHC Database currently includes 96 MHC class I sequences from the UBA locus and 89 MHC class II sequences originating from the DAA and DAB loci. At least for Atlantic salmon, the small number of 112 MHC class I and class II sequences is mainly due to a limited number of studies on MHC diversity. The overall MHC diversity in wild or farmed populations is currently unknown.

MHC has been firmly linked to pathogen resistance in salmonids (Croisetiere et al. 2008; Grimholt et al. 2003; Kjoglum et al. 2008; Langefors et al. 2001; Lohm et al. 2002) making it an important aspect to consider when cultivating wild stock. Specific MHC class II alleles were found to confer resistance towards furunculosis in Atlantic salmon and brook charr *Salvelinus fontinalis*, while other alleles were associated with susceptibility. Most likely, associations between MHC alleles and other pathogens also exist in salmonids, making it highly sensible to ensure that the MHC diversity is preserved in present and future populations.

More than 400 Norwegian watercourses harbour genetically distinct populations of wild Atlantic salmon. A selection of these populations once formed the basis of commercial breeding programmes for farmed Atlantic salmon (Gjedrem 2000). At least for one of our main breeding populations, the continued selection for production traits such as growth has biased the population to a few dominant river strains (Gjedrem et al. 1991). Many wild salmon populations are endangered or vulnerable due to anthropogenic factors and reduced marine survival (Forseth et al. 2017). Release of hatchery-produced eggs, fry or smolt of wild origin has been used to enhance stocks, compensate for the negative effects of hydropower development and to restore stocks decimated by acid precipitation, *G. salaris* and most recently salmon farming. One of the challenges in stock restoration is to ensure genetic representativeness and diversity. Guidelines have thus been developed and implemented by the Norwegian Environment Agency (NorwegianEnvironmentAgency 2014). The use of genetic tools aimed at excluding salmon of farmed origin, avoiding inbreeding and maximising effective population size is now routinely used.

Infectious diseases are severely hampering aquaculture production (Hjeltnes et al. 2019) and there is a growing concern of the impact on wild salmonids (Garseth et al. 2013). In addition, it has been inferred that climate changes can lead to introduction of new hosts and pathogens, increase pathogen development, survival and transmission, but also affect host susceptibility (Harvell et al. 2002). It is therefore imperative to avoid loss of immune diversity in connection with stock restoration programmes. MHC typing and monitoring of immune diversity thus represent a necessary and timely tool in wild salmon restoration programmes.

The Norwegian national salmon river Vosso once held the largest Atlantic salmon in the world, with a unique cultural legacy and considerable local impact on business and recreation (Barlaup 2008). The salmon population collapsed during the 1980s, and although the circumstances were not fully understood, it has been inferred that the population was negatively affected by acid precipitation, hydropower development, road construction and salmon lice during the past 20–30 years (Barlaup 2008). The spawning stock was at a very low level in the 1990s and 2000s, and genetic analysis suggests that the original wild population was replaced by a population affected by escaped farmed salmon during this period (Glover et al. 2012). A rescue operation was thus launched aimed at restoring the Vosso salmon with material collected by the Genebank programme for wild Atlantic salmon during the late 1980s (http://tema.miljodirektoratet.no/en/Areas-of-activity1/Species-and-ecosystems/Salmon-trout-and-Arctic-char/Gene-banks-for-wild-salmon/) (Barlaup 2008).

Both the number of fish species and the number of alleles in the IPD-MHC Database are expected to grow considerably due to the advances in sequencing technology. High-throughput sequencing (HTS) such as Illumina provides a quick and easy way of genotyping many samples in a limited period. Various NGS technology has been tested and compared for human HLA typing (Carapito et al. 2016; Duke et al. 2016), but NGS approaches are also applied to non-human species including MHC class II beta typing for the teleost fish guppy (Lighten et al. 2014).

HTS also provides new challenges when including such transcripts in the IPD-MHC Database. Previously, the sequences should have been identified in three separate PCR reactions thus eliminating jumping PCR artefacts and the sequences needed to include both alpha 1 and alpha 2 domains for class I and at least the alpha 1 or beta 1 domains for class II. What is required for including sequences originating from Illumina studies is currently undefined for fish. Here we develop an Illumina sequence typing protocol for cDNA typing of Atlantic salmon MHC class I and class II alleles, rename existing alleles in the IPD-MHC Database to accommodate HTS and identify aspects needing special attention.

## Material and methods

### Study animals

This study includes head kidney tissue preserved on RNAlater (ThermoFischer) from ten Atlantic salmon captured in River Vosso during the period 2007 to 2009 (denoted AS1-AS10). Samples were obtained during routine health control of brood fish in the stock restoration programme. During this period, scale characters were used to distinguish between salmon of wild, hatchery-reared and farmed origin (Lund and Hansen 1991), and were also used to determine the number of years spent in the river and in the sea, smolt-age and sea-age respectively (Lea 1910; Lee 1920). Catch-year, in combination with smolt-age and sea-age, was subsequently used to select the ten individuals that were not from the same family group (i.e. not siblings).

For the purpose of this study, head kidney samples underwent genetic analyses to identify individuals having farmed salmon in their pedigree (Karlsson et al. 2014; Karlsson et al. 2011). The method has been mandatory in stock enhancement of anadromous salmon since 2014 (Norwegian Environment Agency 2014). The method generates a *p*(wild) value that reflects the “probability of being wild”, with a high value reflecting a high probability of being wild, while salmon with *p*(wild) values < 0.71 is unlikely to be of pure wild origin (Table 1). Based on scale reading, four animals are considered wild and five hatchery-reared. Based on genetic tests (*p*(wild)), six are wild and two to three are the product of variable genetic introgression from farmed salmon.

**Table 1:** Classification of Atlantic salmon animals based on scale readings and probability of being wild (*p*Wild)

Classification\animal	AS1	AS2	AS3	AS5	AS6	AS7	AS8	AS9	AS10
*p*Wild	0.97	0.98	0.33	0.70	0.95	0.50	0.93	0.98	0.98
Scale classification	Hatchery-reared	Hatchery-reared	Hatchery-reared	Hatchery-reared	Wild	Wild	Wild	Wild	Hatchery-reared

### Preparing the sequence library

RNA was isolated from head kidney tissue preserved in RNAlater according to the manufacturer’s recommendation (RNeasy, Qiagen, NL). One of the ten selected animal samples did not pass the RNA quality control (sample AS#4) and was not included further. cDNA was synthesised using 10 ng total RNA according to the manufacturer’s recommendation (QuantiTect Reverse Transcription Kit, Qiagen, NL) and the resultant cDNA was eluted in 35 μl TE. Due to known and unknown sequence variation in the primer regions, we initially tested different forward primers to ensure detection of all allelic variants in the study material (Table 2; primer testing) prior to ordering the Illumina adapter primers. The primers were chosen to comply with overlapping 300 bp paired end sequences for MiSeq v3 sequencing and the design of primer pairs is based on the successful 16S amplicon project described elsewhere (de Muinck et al. 2017).

**Table 2:** PCR primer sequences used in this study

Primer	Sequence	Animals
Test primers		
UBA1_F	CTGGGAATAGGCCTTCTACAT	All
UBA2_F	AGTTGTATCCTTCTGCTGTTCCT	All
UBA3_F	AGCCCTACATTCTTCATCTGC	All
UBA4_F	GGCATCTGCAGTAACCCACT	All
UBA5_F	CTTCGTGAAGCATCTGCTGTG	All
UBA_R	TCCAGATACTTCTTCAGCCAC	All
DAA_F	TGCTGGCAGGTGTATGCAGAA	All
DAA_R	GGTGAAATCAGCGTTGGGGT	All
DAB_F	ATGTCGATGTCTATCTTCTG	All
DAB_R	GTACCAGTCCCCGTTAGCCAG	All
First gene specific Illumina PCR primer sets		
1F_DAA	TCTACACTCTTTCCCTACACGACGCTCTTCCGATCT_TGCTGGCAGGTGTATGCAGAA	All
1R_DAA	GTGACTGGAGTTCAGACGTGTGCTCTTCCGATCT_GGTGAAATCAGCGTTGGGGT	All
1F_DAB	TCTACACTCTTTCCCTACACGACGCTCTTCCGATCT_ATGTCGATGTCTATCTTCTG	AS2, AS3, AS6, AS9, AS10
1F_DAB2	TCTACACTCTTTCCCTACACGACGCTCTTCCGATCT_TTCTGCGTTTCCCTGACCC	AS1, AS5, AS7, AS8
1R_DAB	GTGACTGGAGTTCAGACGTGTGCTCTTCCGATCT_GTACCAGTCCCCGTTAGCCAG	All
1F_UBA1	TCTACACTCTTTCCCTACACGACGCTCTTCCGATCT_CTGGGAATAGGCCTTCTACAT	All except AS5
1F_UBA4	TCTACACTCTTTCCCTACACGACGCTCTTCCGATCT-GGCATCTGCAGTAACCCACT	AS5
1R_UBA	GTGACTGGAGTTCAGACGTGTGCTCTTCCGATCT_TCCAGATACTTCTTCAGCCAC	All
Second PCR primer sets to introduce Illumina indexes		
2F	AATGATACGGCGACCACCGAGATCTACACTCTTTCCCTACACGAC	All
2R_1	CAAGCAGAAGACGGCATACGAGAT_CGTGAT_GTGACTGGAGTTCAGACGTG	AS1
2R_2	CAAGCAGAAGACGGCATACGAGAT_ACATCG_GTGACTGGAGTTCAGACGTG	AS2
2R_3	CAAGCAGAAGACGGCATACGAGAT_GCCTAA_GTGACTGGAGTTCAGACGTG	AS3
2R_4	CAAGCAGAAGACGGCATACGAGAT_TGGTCA_GTGACTGGAGTTCAGACGTG	AS6
2R_5	CAAGCAGAAGACGGCATACGAGAT_CACTGT_GTGACTGGAGTTCAGACGTG	AS7
2R_6	CAAGCAGAAGACGGCATACGAGAT_ATTGGC_GTGACTGGAGTTCAGACGTG	AS8
2R_7	CAAGCAGAAGACGGCATACGAGAT_GATCTG_GTGACTGGAGTTCAGACGTG	AS9
2R_8	CAAGCAGAAGACGGCATACGAGAT_TCAAGT_GTGACTGGAGTTCAGACGTG	AS10
2R_9	CAAGCAGAAGACGGCATACGAGAT_CTGATC_GTGACTGGAGTTCAGACGTG	AS5
Primers to amplify fragments for Sanger sequencing		
DAA-F	TGCTGGCAGGTGTATGCAGAA	AS3 and AS8
DAA-R	GAATGTTCCGGCAGCCACTCC	AS3 and AS8

We used 10 ng of the cDNA in 10 μl PCR reactions for each of the three UBA, DAA and DAB genes with 0.625 units OneTAQ DNA polymerase (NEB Inc., USA), one times standard reaction buffer, 200 μM dNTPs and 0,2 μM each primer. Based on the initial testing, two different forward primers were chosen for amplifying DAB and UBA fragments each, for Illumina sequencing. Twenty-five microliter reactions were performed with the first Illumina primer sets (Table 2; 1F/1R primers) using a PCR reaction mix as described above. Products were verified on a 1% agarose gel prior to cleanup using 1.8 × PCR volume of Agencourt AMPure XP PCR purification kit (Beckman Coulte, Brea, CA, USA) according to the manufacturer’s recommendation and dissolved in 20 μl TE. DNA concentrations and fragment sizes were measured on a Qubit fluorometer (Invitrogen, Carlsbad, CA, USA) and Agilent Bioanalyzer (Santa Clara, CA, USA), respectively.

Libraries containing each of the UBA, DAA and DAB PCR products were blended in proportions to ensure similar coverage and subjected to 10 cycles of PCR using the second set of primers (Table 2; F2+R2), thus adding one unique Illumina index for each animal. This second amplification was carried out with 0.625 units OneTAQ DNA polymerase (NEB Inc., USA), one times standard reaction buffer, 200 μM dNTPs, 0.2 μM each primer and 10 μl template pool at 3 ng/μl. The following programme was used for amplification: 94 °C for 2 min; ten cycles of 94 °C for 30 s, 58 °C for 30 s, 72 °C for 60 s; 72 °C for 10 min.

The nine resultant PCR pools were subjected to two additional AMPure cleanups using a 1:1 ration to eliminate shorter fragments. Based on data from Bioanalyzer and Qubit, the nine PCR pools were mixed totalling 2 μg DNA in 130 μl TE-1 buffer (10 mM Tris-HCl, 0.1 mM disodium EDTA, pH 8.0) as recommended for Illumina MiSeq sequencing. qPCR was performed to check the library size before proceeding with sequencing. Sequencing was performed on Illumina MiSeq (Illumina, USA) platform using the v3 chemistry to achieve 300 bp paired end reads.

### Bioinformatic analyses

**Fig. 1:**
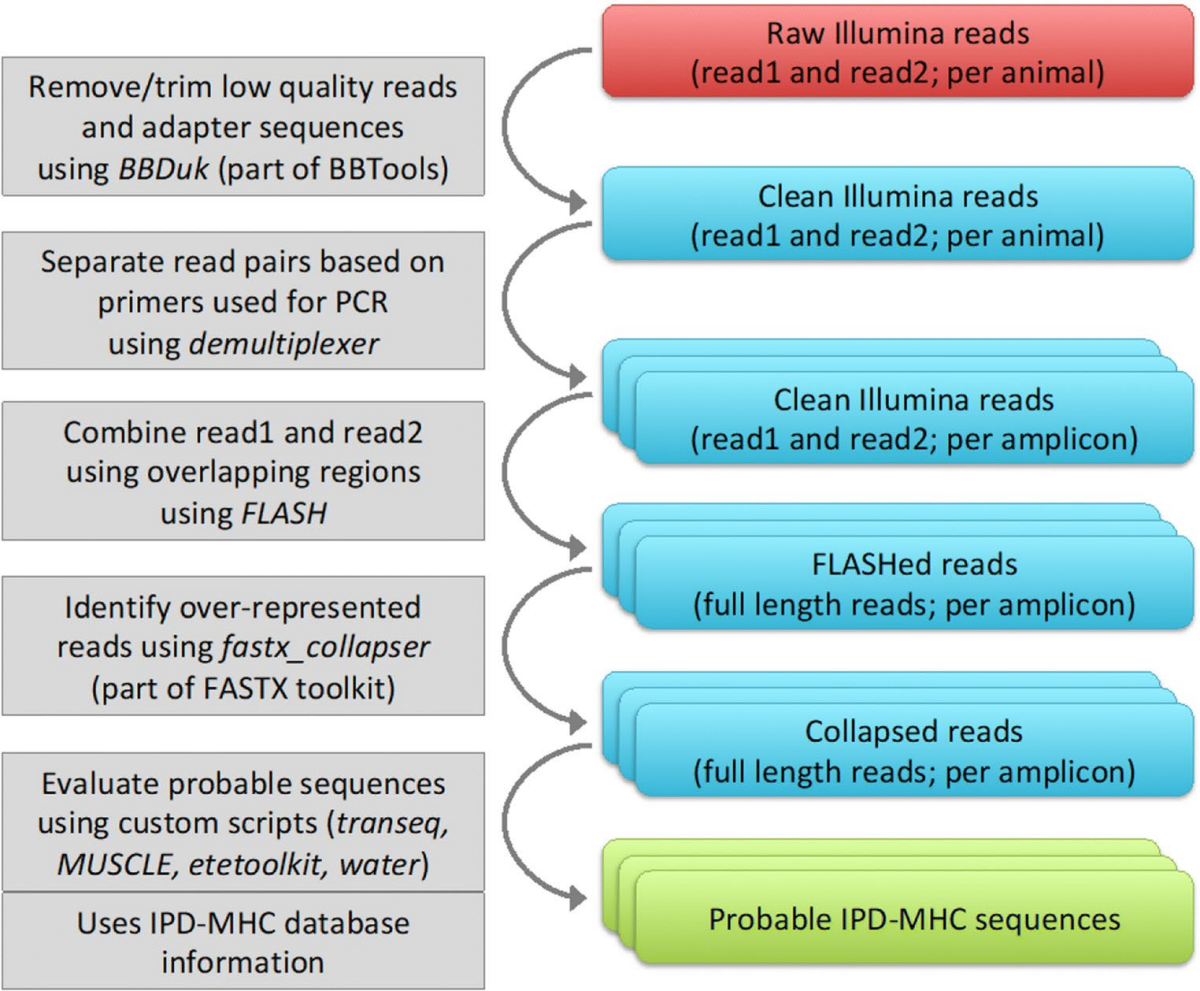
Data analysis flowchart. Flowchart describes the analysis workflow used in this study and explained in detail under the “Bioinformatic analyses” section within the “Materials and Methods” section. All the tools are available as open source software/tool and the custom script used in the last step can be found at https://github.com/NorwegianVeterinaryInstitute/Salmonid_MHC_classifier

Illumina raw reads (fastq format; read 1 and read 2 pairs) were obtained for each animal as the sequence data was demultiplexed using the Illumina index introduced during the second PCR reaction. The bioinformatic pipeline described below is also explained in Fig. 1 as a flowchart. Sequence data has been submitted to NCBI SRA under the BioProject accession number PRJNA578031.

Data for each animal was processed using BBDuk v34.56 (part of BBTools; https://jgi.doe.gov/data-and-tools/bbtools/bb-tools-user-guide/) to remove/trim bad quality reads and sequencing adapter sequences. Cleaned reads were further demultiplexed based on the primers used during the first PCR reaction using demultiplexer v1.7 (https://github.com/nsc-norway/triple_index-demultiplexing) allowing zero mismatches between the primers and the sequenced reads. This step separates the reads into each MHC subgroups as targeted during the first PCR reaction and removes the primer sequences from the reads.

Read 1 and read 2 for each MHC subgroup were combined using FLASH v1.2.11 (Magoc and Salzberg 2011) with default settings (-r 300 was used to specify the read length). FLASH uses the overlapping information between the paired reads to combine them into one long read. The resulting full-length amplified reads were collapsed to identity all the unique reads and sorted based on the number of times it was present in the data using fastx_collapser (part of FASTX Toolkit v0.0.13; http://hannonlab.cshl.edu/fastx_toolkit/). The top five most represented (1–2% of the FLASHed reads) full-length amplified reads (Fasta format) were further processed.

Potential MHC allele sequences identified using the above approach were evaluated using custom python scripts (https://github.com/NorwegianVeterinaryInstitute/Salmonid_MHC_classifier). 0–2 nucleotides were removed from either/both ends of the sequences to accommodate frame shift issues while converting to amino acid sequences before proceeding with the analyses.

The scripts were developed in collaboration with the IPD-MHC Database to use a library of official alleles to identify the closer match to the input sequence. The scripts automatically retrieve relevant information from the IPD-MHC Database, thus facilitating the analysis and identification of novel sequences against the up-to-date dataset.

The input fasta records were converted to amino acid sequences using transeq [part of EMBOSS v6.6.0.0; (Rice et al. 2000)] followed by multiple sequence alignment (only nucleotide) with relevant IPD-MHC Database entries using MUSCLE v3.8.1551 (Edgar 2004). Closest clade/sibling information from the tree produced by MUSCLE was extracted using python module ETE toolkit (Huerta-Cepas et al. 2016). Sequence similarity and identity between the fasta record and the closest sibling was calculated using Water [part of EMBOSS v6.6.0.0; (Rice et al. 2000)] alignment tool for both nucleotide and amino acid sequences, respectively. A report file was generated with all the relevant information for each fasta record and the user can make an educated evaluation regarding the nomenclature and submit the probable MHC sequences to the IPD-MHC Database for further verification and official name assignment.

### Phylogenetic analysis

Amino acid sequence alignments were performed in ClustalX (Larkin et al. 2007) after 5′ and 3′ sequences including primer sequences were removed using Jalview 2 (Waterhouse et al. 2009). The evolutionary history was inferred using the maximum likelihood method based on the Whelan and Goldman model (Whelan and Goldman 2001). The percentage of trees in which the associated taxa clustered together is shown next to the branches. Initial tree(s) for the heuristic search were obtained automatically by applying Neighbour-Join and BioNJ algorithms to a matrix of pairwise distances estimated using a JTT model, and then selecting the topology with superior log likelihood value. A discrete Gamma distribution was used to model evolutionary rate differences among sites (5 categories (+G, parameter = 0.0515)). The trees are drawn to scale, with branch lengths measured in the number of substitutions per site. All positions with less than 95% site coverage were eliminated. That is, fewer than 5% alignment gaps, missing data and ambiguous bases were allowed at any position. Evolutionary analyses were conducted in MEGA7 (Kumar et al. 2016).

### Sanger sequencing

To decipher between some MHC class II alpha alleles, we amplified cDNA fragments from selected animals using primers shown in Table 2. The PCR was performed as described above and fragments were cloned into the pCR2.1 vector (ThermoFischer) and transformed into Oneshot Top10 competent cells (ThermoFischer) and individual clones were sequenced using BigDye Terminator 3.1 (Applied Biosystems) according to the manufacturer’s protocol.

## Results and discussion

### New nomenclature

Salmonid MHC alleles are currently denoted according to the set of guidelines promoted by the MHC Nomenclature committee (Maccari et al. 2018), where a unique four-letter code identifying the organism is followed by the gene name and an allele number. For example, MHC-Sasa-DAA*0101 or Sasa-DAA*0101 for short, Sasa-DAA*0102 etc. where Sasa denotes *Salmo salar*, DAA is the locus MHC class II alpha and *0101 denotes the first sequence in this sequence group. The second allele in this sequence group is denoted *0102. To represent a new sequence group, 4 amino acid differences are required between class I alleles, and 3 amino acid differences are required for class II alleles. In the case of silent mutations, when the amino acid sequences are identical but the nucleotide sequences are different, a third double-digit number is added e.g. Sasa-DAA*010101, Sasa-DAA*010102 etc.

In order to facilitate the comparison of genomic data, this paper introduces the use of the human MHC nomenclature (Marsh et al. 2010) to describe allele variation in fish, as suggested for non-human species (Maccari et al. 2018). The gene prefix name is identical i.e. MHC-Sasa -UBA* defines an allele originating from the UBA gene of Atlantic salmon. The allele group is shown using a two-digit number (Sasa -UBA*01) where individual groups have four amino acid differences for class I and three amino acid differences for class II. The specific protein is shown with an additional two digits divided by a colon (Sasa UBA*01:01). Synonymous substitution in coding region is represented using an additional two-three digit introduced after an additional colon (Sasa -UBA*01:01:01). Additional differences in non-coding regions are shown using another set of two-digit number (Sasa -UBA*01:01:01:01) (see Supplementary File 1). The same nomenclature will be also applied for the MHC alleles from rainbow trout (see Supplementary File 1).

### Sequence analyses—scripts linked to IPD-MHC

Illumina sequencing yielded 0.65–1.4 M read pairs per animal and out of these more than 82% were retained after removing/trimming low-quality reads and adapter sequences. Further demultiplexing the data using the primers used for amplification provided more than 97,000 read pairs per MHC group for each animal. FLASH was able to combine 50–90% of these read pairs based on the overlapping regions, which provide 95–395,000 full-length amplified reads per group for each animal (Supplementary file 2: Sheet1).

Collapsing the reads using fastx_collapser to identify the most represented unique reads found 26–40% of the reads being represented by one allele (Supplementary file 2: Sheet2). Out of the 27 groups (3 groups for 9 animals), 8 had one over-represented allele while the rest had two or more. The percentage difference between the first and second most over-represented allele was much pronounced in DAA and UBA while the difference was negligible across DABs. The top five nucleotide and deduced amino acid sequences for each gene in each animal are listed in Supplementary file 3.

We established our library and bioinformatics pipeline using only nine animals. However, based on results, this Illumina Miseq v3 approach could most likely be adapted to 96 animals, three genes each.

### MHC analysis

To prepare for Illumina typing of MHC alleles in a new population, one needs to identify primers that both comply with the read length of the Illumina sequencing mode but also ensure that all alleles in that populations are identified using the chosen set of primers. In particular, the sequence variation in the leader sequence region of UBA alleles (Supplementary file 4) makes it necessary to test a variety of forward primers to ensure that all alleles are represented in the final library. We found that only two of the five tested primers produced fragments for UBA in our animals. We also used two different MHC class II beta forward primers as primer efficiency varied between animals. For MHC class II alpha, all animals showed good amplification using just one primer set.

The sequences discussed below are given names to identify animal i.e.AS1 to AS10 (AS4 did not pass RNA quality and thus not present in the analysis) and allele class as follows: class II alpha is DAA plus a number referring to the sequence number found in the MiSEq v3 data analysis. Thus, AS1_DAA_s1 would refer to the collapsed DAA sequence with the highest number of reads found in the data analysis of animal number 1. Class II beta is denoted DAB and class I is denoted UBA making AS1_DAB1_s1 and AS1_UBA1-1 the DAB and the UBA sequences with highest number of reads found in the analyses of sequences from animal number 1 using the forward primers DAB1 and UBA1.

**Fig. 2:**
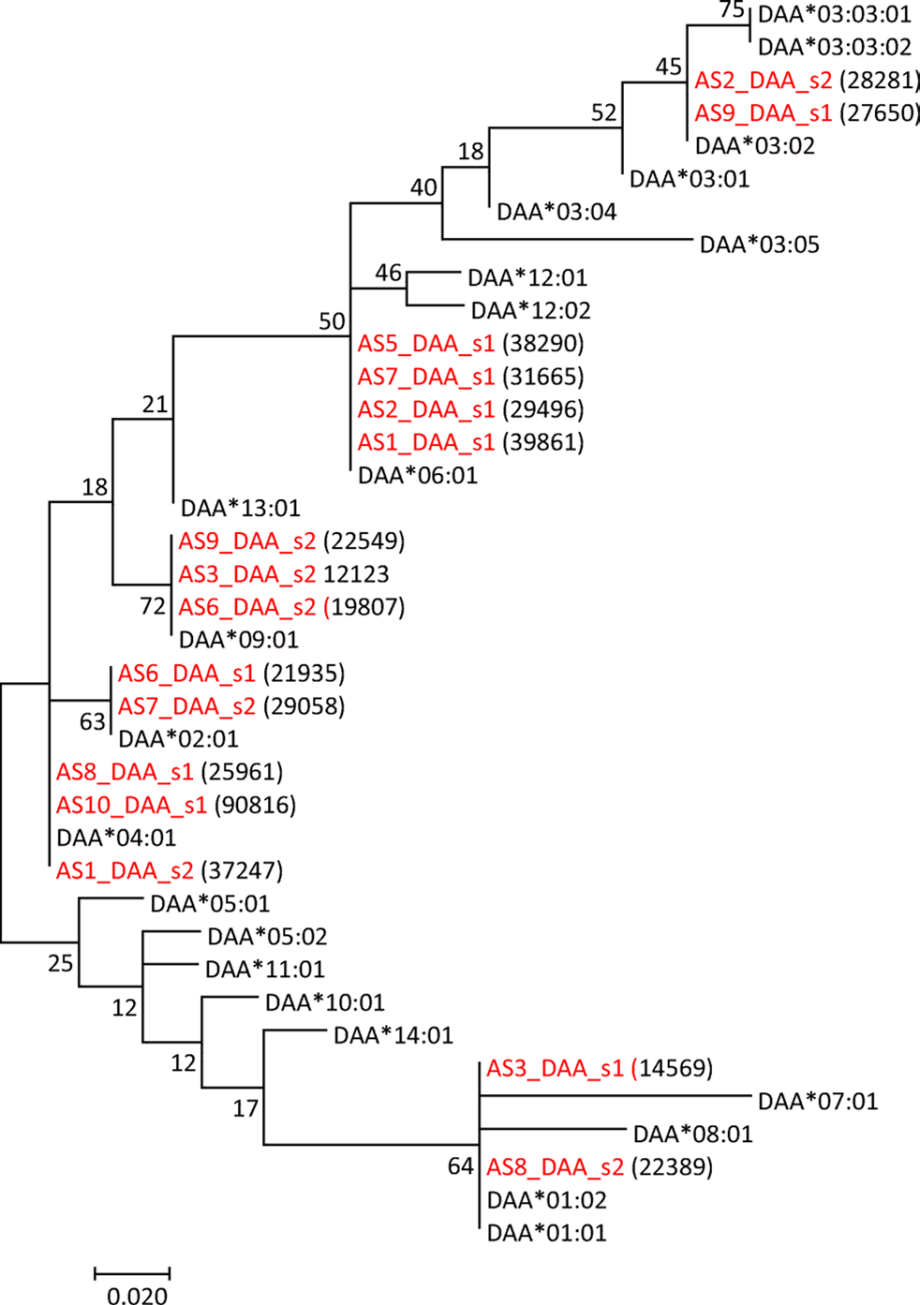
DAA tree. Evolutionary relationships of MHC class II alpha DAA amino acid sequences. Sequences originating from our dataset are shown using red font. A number of Illumina reads per sequence are shown in parenthesis. The tree with the highest log likelihood (− 444.69) is shown. The percentage of trees in which the associated taxa clustered together is shown next to the branches. A discrete Gamma distribution was used to model evolutionary rate differences among sites (5 categories (+G, parameter = 0.0515)). The analysis involved 38 amino acid sequences. There were a total of 70 positions in the final dataset

Seven of nine animals were heterozygous for MHC class II alpha (Table 3). We found six alleles in the material, all present in the current IPD-MHC Database (Fig. 2, Supplementary file 5). Seven of nine animals were heterozygous where only animal AS5 and AS10 were homozygous. Based on the amplified region, we could not determine if the AS3_DAA3_s1 and AS8_DAA8_s2 sequences were DAA*01:01 or DAA*01:02. We thus PCR amplified more of the coding region, cloned and Sanger sequenced fragments from the two samples and found them to be DAA*01:02 in both animals (data not shown).

**Table 3:** MHC alleles identified in Atlantic salmon animals

Animal allele	AS1	AS2	AS3	AS5	AS6	AS7	AS8	AS9	AS10
DAA_1	DAA*06:01 S1_39861	DAA*06:01 S1_29496	DAA*01:02^1^ S1_14569	DAA*06:01 S1_38290	DAA*02:01 S1_21935	DAA*06:01 S1_31665	DAA*04:01 S1_25961	DAA*03:02 S1_27650	DAA*04:01 S1_90816
DAA_2	DAA*04:01 S2_37247	DAA*03:02 S2_28281	DAA*09:01 S2_12123	0	DAA*09:01 S2_19807	DAA*02:01 S2_29058	DAA*01:02^1^ S2_22389	DAA*09:01 S2_22549	0
DAB_1	DAB*09:01 2s1_63791	DAB*20:01 1s1_30852	DAB*07:01 1s1_10179	DAB*06:01 2S1_11545	DAB*09:01 1s1_10396	DAB*02:01 2s1_71872	DAB*08:01 2s2_29683	DAB*07:01 1s1_16301	DAB*09:01 1s1_74482
DAB_2	DAB*06:01 2S2_20621	DAB*06:01 1S2_17876	DAB*08:01 1s2_7127	AS5DABs2 2s2_9933	DAB*02:01 1s2_9420	DAB*06:01 2S2_24758	DAB*09:02 2s1_41329	DAB*20:01 1s2_10525	0
UBA_1	UBA*07:01 1s1_30927	AS2UBAs1 1s1_46437	UBA*07:01 1s1_13465	AS5UBAs1 2s1_22247	UBA*13:01 1s1_18686	AS7UBAs1 1s1_25193	UBA*13:01 1s1_30959	UBA*13:01 1s1_20357	UBA*34:01 1s1_71332
UBA_2	0	AS2UBAs2 1s2_33647	UBA*13:01 1s2_12935	AS5UBAs2 2s2_20661	UBA*02:01 1s2_3956	UBA*20:01 1s2_6454	UBA*06:01 1s2_24959	AS9UBAs2 1s2_14112	0
Scale class	Hatchery-reared	Hatchery-reared	Hatchery-reared	Hatchery-reared	Wild	Wild	Wild	Wild	Hatchery-reared

Animal no. 4 is missing, as RNA did not pass quality control. The two allelic sequences per gene per animal are shown with specific primer for DAB and UBA and matching number of reads per each of the two allelic sequences (s1 or s2). The underscore between animal number and gene and sequence number are not shown for the new allelic sequences due to space restrictions. ^1^Illumina sequence did not discriminate between DAA*01:01 and DAA*01:02 so verification using other primers and Sanger sequencing was performed to identify allele

A few of the animals show more than two MHC sequences with considerable support in number of collapsed reads. For instance, for AS2_DAA, the two top sequences are supported by more than 28,000 reads, but the next three sequences are supported by more than 3700 reads (Supplementary files 2-3). Aligning these five sequences shows a pattern of jumping PCR i.e. one MHC allele is partly elongated during one PCR cycle, then denatured and then the sequence re-associates with another allele for further elongation. This means that all the variable sites have amino acids from one of the two alleles but in different combinations. We used this approach to exclude the third highest supported sequence for all classes of genes, thus supporting our expectation of only two alleles per animal.

**Fig. 3:**
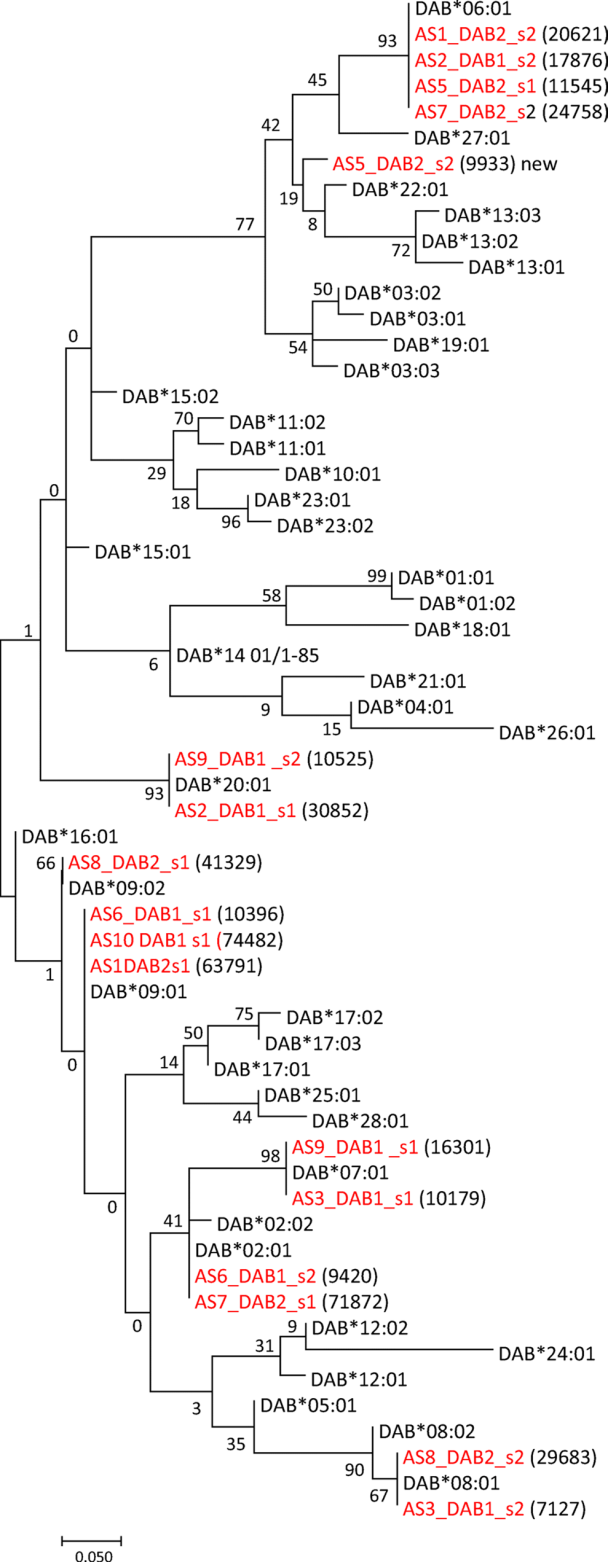
DAB tree. Evolutionary relationships of MHC class II beta DAB amino acid sequences. Sequences originating from our dataset are shown using red font. A number of Illumina reads per sequence are shown in parenthesis. The tree with the highest log likelihood (− 1093.62) is shown. The percentage of trees in which the associated taxa clustered together is shown next to the branches. A discrete Gamma distribution was used to model evolutionary rate differences among sites (5 categories (+G, parameter = 0.2153)). There were a total of 85 positions in the final dataset

Eight of our nine animals were heterozygous for MHC class II beta (Table 3). We identified eight DAB alleles in our study material where seven were already included in the IPD-MHC database (Fig. 3, Supplementary file 5). Our DAB*09:01 allele sequence is an extension of IPD-MHC allele sequence. As the DAA and DAB genes are closely linked on chromosome 12, only separated by 3 kb, we expect these alleles to segregate as haplotypes with specific combinations of alpha and beta alleles. Two of the seven haplotypes we identified in a previous study (Grimholt et al. 2003) were also found in this material, i.e. DAA*02:01-DAB*02:01 and DAA*06:01-DAB*06:01. Four new haplotypes were supported by more than one animal, i.e. DAA*01:02-DAB*08:01 present in animals AS3 and AS8, DAA*03:02-DAB*20:01 present in animals AS2 and AS9, DAA*04:01-DAB*09:01 found in animals AS1 and AS10 and DAA*09:01-DAB*07:01 found in animals AS3 and AS9. The DAA*01:02-DAB*08:01 haplotype found in animals AS3 and AS8 differs from the DAA*01:01-DAB*08:01 haplotype found in our previous study in only one DAA*01:02 amino acid. Previously, we found the DAA*04:01-DAB*07:01 haplotype, but in this material, DAA*04:01 seems linked to either DAB*09:01 or DAB*09:02, where only one amino acid separates DAB*09:01 from DAB*09:02. Judging by the two additional haplotypes DAA*09:01-DAB*09:01 and DAA*04:01-DAA*09:02, this may suggest that there has been a crossing over between the DAA*04:01-DAA*07:01 and DAA*09:01-DAB*09:01 haplotypes providing the new DAA*09:01-DAB*07:01 and DAA*04:01-DAB*09:01 haplotypes found in this study. A problematic issue is the fact that AS5 has only one DAA allele, but two DAB alleles where one of the DAB alleles is new (Fig. 3, AS5_DAB2_s2_9933). One explanation is that the DAA*06:01 allele segregates with two different DAB alleles, where the DAB sequences differ by four amino acids each supported by a similar number of collapsed reads. Comparing our Vosso population with our previously MHC typed farmed population, there does not seem to be a stable link between MHC class II alpha and MHC class II beta alleles making it necessary to genotype both the alpha and the beta alleles.

**Fig. 4:**
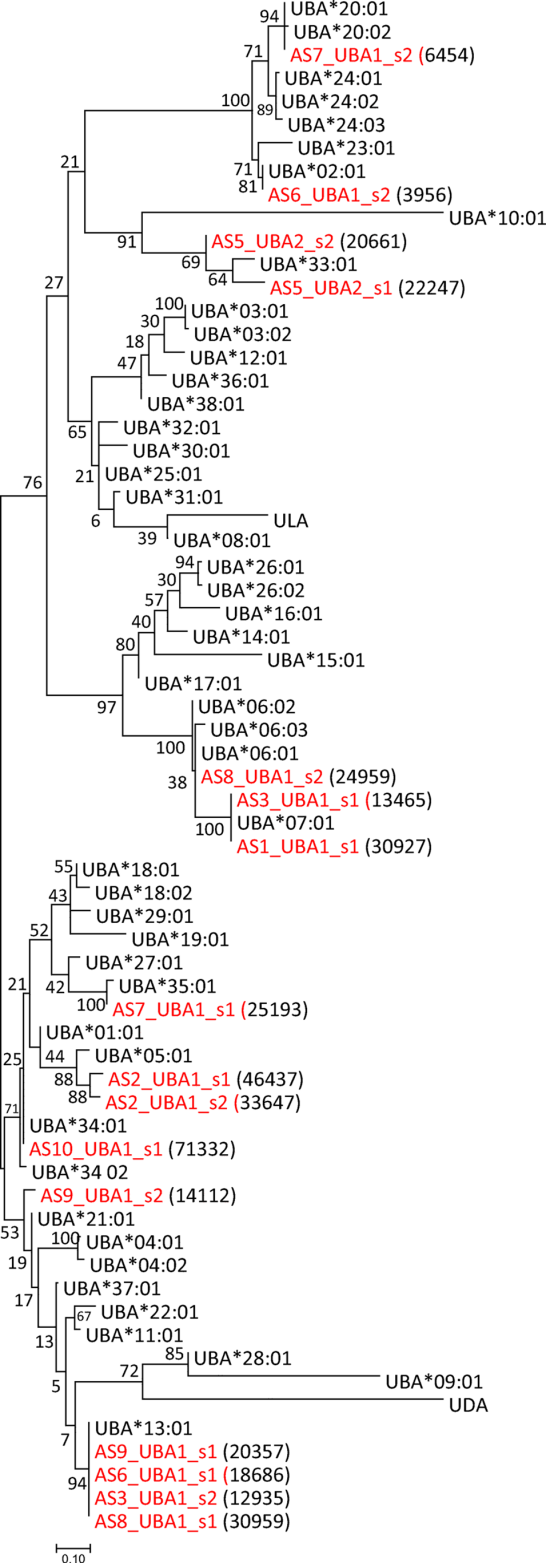
U lineage tree. Evolutionary relationships of MHC class I U lineage amino acid sequences. Sequences originating from our dataset are shown using red font. A number of Illumina reads per sequence are shown in parenthesis. The tree with the highest log likelihood (− 4391.31) is shown. The percentage of trees in which the associated taxa clustered together is shown next to the branches. A discrete Gamma distribution was used to model evolutionary rate differences among sites (5 categories (+G, parameter = 0.6296)). The analysis involved 66 amino acid sequences. There were a total of 160 positions in the final dataset

Seven of our nine animals were also heterozygous for MHC class I (Table 3) representing a total of twelve MHC class I UBA alleles (Fig. 4, Supplementary file 5). The five alleles UBA*02:01, UBA*06:01, UBA*13:01, UBA*20:01 and UBA*34:01 were already present in the IPD-MHC database. Two of these alleles, i.e. AS6_UBA1_s2_3956 is UBA*02:01 and AS7_UBA1_s2_6454 is UBA*20:01, had lower number of collapsed read support than the remaining sequences defined as alleles. We chose to explain this by efficiency differences in PCR primers rather than being contaminations. One allele differed only slightly from an IPD-MHC alleles i.e. AS7_UBA_s1 differing in two amino acids from UBA*35:01 and thus qualifies for being named UBA*35:02. The six remaining allele sequences differed with more than four amino acids from existing alleles and thus represent new IPD-MHC alleles (Fig. 4, Supplementary files 3 and 5; AS2_UBA1_s1_46537, AS2_UBA1_s2_33647, AS5_UBA2_s1_22247, AS5_UBA2_s2_20661, AS7_UBA1_s1_25193, AS9_UBA1_s2_14112).

UBA alleles have highly diverse alpha 1 domain sequences where different lineages are shared between distantly related species (Aoyagi et al. 2002; Grimholt et al. 2015; Kiryu et al. 2005). These alpha 1 domain lineage sequences are then combined with different lineages of alpha 2 domain and downstream sequences potentially due to recombination in the large intron between the alpha 1 and alpha 2 domains. This is clearly visible for the new allele AS7_UBA1_s1 which has an alpha 1 domain identical to the UBA*27:01 allele, but then the alpha 2 domain is similar to e.g. UBA*35:01. Another example is AS9_UBA1_s2 which shares an alpha 1 domain with for instance UBA*22:01 while the alpha 2 domain sequence is similar to for instance UBA*21:01. This combination of different alpha 1 and alpha 2 domain sequences is a very good argument for amplifying as much from both regions as possible to enable correct allele identification.

### Requirements for including new allelic MHC sequences into the IPD-MHC fish database

We identified seven new MHC alleles in this study, i.e. one DAB sequence (AS5_DAB2_s2_9933) and six UBA sequences (AS2_UBA1_s1_46537, AS2_UBA1_s2_33647, AS5_UBA2_s1_22247, AS5_UBA2_s2_20661, AS7_UBA1_s1_25193, AS9_UBA1_s2_14112). These new sequences need to be verified using new PCR and Sanger sequencing prior to submission to the IPD-MHC Database for an official name assigned. Preferentially most of the coding region should be amplified, at least for UBA, it is required that submitted sequences include the three extracellular domain as well as the transmembrane domain, as the allele needs to be verified as UBA and not another U lineage sequence.

### MHC diversity in the Vosso population

The selected material showed unexpected diversity. This may have been caused by genetic introgression from farmed escapees (Glover et al. 2012) and by straying—that salmon fails to return to their native river. About 3–6% of wild salmon and 15% of hatchery-reared salmon may stray to other rivers during homeward spawning migration (Jonsson et al. 1991; Jonsson et al. 2003; Stabell 1984). Studies show that most of the straying salmon will enter nearby rivers (Jonsson et al. 2003). A verdict on which alleles belong to the original Vosso population and what originates from farmed fish could be resolved genotyping scales sampled prior to 1980s, i.e. before aquaculture appeared in these fjords. However, that would require a different strategy for Illumina typing than the one presented here.

## Conclusion

We have established a library preparation and bioinformatics analysis pipeline using Illumina MiSeq v3 paired end sequencing for MHC cDNA. This pipeline enables studies into salmonid MHC diversity among different strains in local rivers as well as breeding populations, thus expanding our knowledge on salmonid MHC diversity. To accommodate this IPD-MHC sequence expansion, we updated the IPD-MHC fish nomenclature for both Atlantic salmon and for rainbow trout following the MHC Nomenclature Committee guidelines, allowing the unambiguous naming and comparison of genomic data. Furthermore, the obtained haplotype data will be included into the next release of the IPD-MHC Database (December 2019) to enrich the information available in the IPD-MHC fish section.

We established the pipeline on Atlantic salmon animals from an endangered river strain and found a surprisingly high number of different alleles, with seven newly identified alleles (one Sasa-DAB and six Sasa UBA). Most likely, this diversity reflects interference from farmed Atlantic salmon in addition to potential straying from nearby river populations. To test allele changes over time, additional typing strategies need to be developed enabling genotyping using genomic DNA from historically preserved fish scales.

### Electronic supplementary material

Supplementary file 1. New IPD-MHC Fish nomenclature (PDF 725 kb)

Supplementary file 2. Illumina Statistics (PDF 518 kb)

Supplementary file 3. MHC sequences (PDF 589 kb)

Supplementary file 4. UBA primer regions (PDF 543 kb)

Supplementary file 5. MHC sequence alignments (PDF 1303 kb)

## References

[CR1] Aoyagi K, Dijkstra JM, Xia C, Denda I, Ototake M, Hashimoto K, Nakanishi T (2002). Classical MHC class I genes composed of highly divergent sequence lineages share a single locus in rainbow trout (*Oncorhynchus mykiss*). J Immunol.

[CR2] Bannai HP, Nonaka M (2013). Comprehensive analysis of medaka major histocompatibility complex (MHC) class II genes: implications for evolution in teleosts. Immunogenetics.

[CR3] Barlaup BTE (2008) Now or never for the Vosso salmon- recommended actions based on population development and threat factors. Norwegian Environment Agency, https://www.miljodirektoratet.no/globalassets/dokumenter/publikasjoner/overvakingsrapporter/vossolaksen_rapport.pdf

[CR4] Bingulac-Popovic J, Figueroa F, Sato A, Talbot WS, Johnson SL, Gates M, Postlethwait JH, Klein J (1997). Mapping of mhc class I and class II regions to different linkage groups in the zebrafish, *Danio rerio*. Immunogenetics.

[CR5] Carapito R, Radosavljevic M, Bahram S (2016). Next-generation sequencing of the HLA locus: methods and impacts on HLA typing, population genetics and disease association studies. Hum Immunol.

[CR6] Christensen KA, Leong JS, Sakhrani D, Biagi CA, Minkley DR, Withler RE, Rondeau EB, Koop BF, Devlin RH (2018). Chinook salmon (*Oncorhynchus tshawytscha*) genome and transcriptome. PLoS One.

[CR7] Christensen KA, Rondeau EB, Minkley DR, Leong JS, Nugent CM, Danzmann RG, Ferguson MM, Stadnik A, Devlin RH, Muzzerall R, Edwards M, Davidson WS, Koop BF (2018). The Arctic charr (*Salvelinus alpinus*) genome and transcriptome assembly. PLoS One.

[CR8] Croisetiere S, Tarte PD, Bernatchez L, Belhumeur P (2008). Identification of MHC class IIbeta resistance/susceptibility alleles to *Aeromonas salmonicida* in brook charr (*Salvelinus fontinalis*). Mol Immunol.

[CR9] de Muinck EJ, Trosvik P, Gilfillan GD, Hov JR, Sundaram AYM (2017). A novel ultra high-throughput 16S rRNA gene amplicon sequencing library preparation method for the Illumina HiSeq platform. Microbiome.

[CR10] Dijkstra JM, Katagiri T, Hosomichi K, Yanagiya K, Inoko H, Ototake M, Aoki T, Hashimoto K, Shiina T (2007). A third broad lineage of major histocompatibility complex (MHC) class I in teleost fish; MHC class II linkage and processed genes. Immunogenetics.

[CR11] Dijkstra JM, Grimholt U, Leong J, Koop BF, Hashimoto K (2013). Comprehensive analysis of MHC class II genes in teleost fish genomes reveals dispensability of the peptide-loading DM system in a large part of vertebrates. BMC Evol Biol.

[CR12] Duke JL, Lind C, Mackiewicz K, Ferriola D, Papazoglou A, Gasiewski A, Heron S, Huynh A, McLaughlin L, Rogers M, Slavich L, Walker R, Monos DS (2016). Determining performance characteristics of an NGS-based HLA typing method for clinical applications. HLA.

[CR13] Edgar RC (2004). MUSCLE: multiple sequence alignment with high accuracy and high throughput. Nucleic Acids Res.

[CR14] Forseth T (2017). The major threats to Atlantic salmon in Norway. ICES J Mar Sci.

[CR15] Garseth AH, Ekrem T, Biering E (2013). Phylogenetic evidence of long distance dispersal and transmission of piscine reovirus (PRV) between farmed and wild Atlantic salmon. PLoS One.

[CR16] Gjedrem T (2000) Genetic improvement of cold-water fish species. Aquac Res:25-33 doi:10.1046/j.1365-2109.2000.00389.x

[CR17] Gjedrem T, Gjøen HM, Gjerde B (1991). Genetic origin of Norwegian farmed Atlantic salmon. Aquaculture.

[CR18] Glover K, Quintela M, Wennevik V, Besnier F, Sørvik AGE (2012). Three decades of farmed escapees in the wild: a spatio-temporal analysis of Atlantic salmon population genetic structure throughout Norway. PLoS One.

[CR19] Grimholt U (2016) MHC and evolution in teleosts. Biology (Basel) 5 doi:10.3390/biology5010006 10.3390/biology5010006 PMC4810163 26797646

[CR20] Grimholt U, Larsen S, Nordmo R, Midtlyng P, Kjoeglum S, Storset A, Saebø S, Stet RJ (2003). MHC polymorphism and disease resistance in Atlantic salmon (*Salmo salar*); facing pathogens with single expressed major histocompatibility class I and class II loci. Immunogenetics.

[CR21] Grimholt U, Tsukamoto K, Azuma T, Leong J, Koop BF, Dijkstra JM (2015) A comprehensive analysis of teleost MHC class I sequences. BMC Evol Biol 15. 10.1186/s12862-015-0309-1 10.1186/s12862-015-0309-1 PMC4364491 25888517

[CR22] Grimholt U, Tsukamoto K, Hashimoto K, Dijkstra JM (2019) Discovery of a novel MHC class I lineage in teleost fish which shows unprecedented levels of ectodomain deterioration while possessing an impressive cytoplasmic tail motif. Cells 8 doi:10.3390/cells8091056 10.3390/cells8091056 PMC6769792 31505831

[CR23] Harvell CD, Mitchell CE, Ward JR, Altizer S, Dobson AP, Ostfeld RS, Samuel MD (2002). Climate warming and disease risks for terrestrial and marine biota. Science.

[CR24] Hjeltnes B, Bang Jensen B, Bornø G, Haukaas A, Walde CS (2019) Fish Health Report 2018. Norwegian Veterinary Institute, https://www.vetinst.no/rapporter-og-publikasjoner/rapporter/2019/fiskehelserapporten-2018

[CR25] Huerta-Cepas J, Serra F, Bork P (2016). ETE 3: reconstruction, analysis, and visualization of phylogenomic data. Mol Biol Evol.

[CR26] Jonsson B, Jonsson N, Hansen LP (1991). Differences in life-history and migratory behavior between wild and hatchery-reared Atlantic salmon in nature. Aquaculture.

[CR27] Jonsson B, Jonsson N, Hansen LP (2003). Atlantic salmon straying from the River Imsa. J Fish Biol.

[CR28] Karlsson S, Moen T, Lien S, Glover KA, Hindar K (2011). Generic genetic differences between farmed and wild Atlantic salmon identified from a 7K SNP-chip. Mol Ecol Resour.

[CR29] Karlsson S, Diserud OH, Moen T, Hindar K (2014). A standardized method for quantifying unidirectional genetic introgression. Ecol Evol.

[CR30] Kiryu I, Dijkstra JM, Sarder RI, Fujiwara A, Yoshiura Y, Ototake M (2005) New MHC class Ia domain lineages in rainbow trout (*Oncorhynchus mykiss*) which are shared with other fish species. Fish Shellfish Immun:243-254 doi:10.1016/j.fsi.2004.07.007 10.1016/j.fsi.2004.07.007 15519543

[CR31] Kjoglum S, Larsen S, Bakke HG, Grimholt U (2008). The effect of specific MHC class I and class II combinations on resistance to furunculosis in Atlantic salmon (*Salmo salar*). Scand J Immunol.

[CR32] Klein J (1986). The natural history of the major histocompatibility complex.

[CR33] Kumar S, Stecher G, Tamura K (2016). MEGA7: molecular evolutionary genetics analysis version 7.0 for bigger datasets. Mol Biol Evol.

[CR34] Langefors A, Lohm J, Grahn M, Andersen O, von Schantz T (2001). Association between major histocompatibility complex class IIB alleles and resistance to *Aeromonas salmonicida* in Atlantic salmon. ProcBiolSci.

[CR35] Larkin MA, Blackshields G, Brown NP, Chenna R, McGettigan P, McWilliam H, Valentin F, Wallace IM, Wilm A, Lopez R, Thompson JD, Gibson TJ, Higgins DG (2007). Clustal W and Clustal X version 2.0. Bioinformatics.

[CR36] Lea E (1910) On the methods used in the Herring-investigations. Conseil permanent international pour l’exploration de la mer. vol 53. Publ. de Circonst. Copenhagen, Denmark

[CR37] Lee RM (1920) A review of the methods of age and growth determination in fishes by means of scales. Fisheries Investigations London Series 2:1–32

[CR38] Lighten J, van Oosterhout C, Paterson IG, McMullan M, Bentzen P (2014). Ultra-deep Illumina sequencing accurately identifies MHC class IIb alleles and provides evidence for copy number variation in the guppy (*Poecilia reticulata*). Mol Ecol Resour.

[CR39] Lohm J, Grahn M, Langefors A, Andersen O, Storset A, von Schantz T (2002) Experimental evidence for major histocompatibility complex-allele-specific resistance to a bacterial infection. Proc Biol Sci 269:2029–2033. 10.1098/rspb.2002.2114 10.1098/rspb.2002.2114 PMC1691130 12396502

[CR40] Lukacs MF (2007). Genomic organization of duplicated major histocompatibility complex class I regions in Atlantic salmon (*Salmo salar*). BMC Genomics.

[CR41] Lund RA, Hansen LP (1991) Identification of wild and reared Atlantic salmon, *Salmo salar* L., using scale characters. Aquac Res 22(4):499-508

[CR42] Maccari G, Robinson J, Ballingall K, Guethlein LA, Grimholt U, Kaufman J, Ho CS, de Groot NG, Flicek P, Bontrop RE, Hammond JA, Marsh SG (2017). IPD-MHC 2.0: an improved inter-species database for the study of the major histocompatibility complex. Nucleic Acids Res.

[CR43] Maccari G, Robinson J, Bontrop RE, Otting N, de Groot NG, Ho CS, Ballingall KT, Marsh SGE, Hammond JA (2018). IPD-MHC: nomenclature requirements for the non-human major histocompatibility complex in the next-generation sequencing era. Immunogenetics.

[CR44] Magoc T, Salzberg SL (2011). FLASH: fast length adjustment of short reads to improve genome assemblies. Bioinformatics.

[CR45] Marsh SG, Albert ED, Bodmer WF, Bontrop RE, Dupont B, Erlich HA, Fernández-Viña M, Geraghty DE, Holdsworth R, Hurley CK, Lau M, Lee KW, Mach B, Maiers M, Mayr WR, Müller CR, Parham P, Petersdorf EW, Sasazuki T, Strominger JL, Svejgaard A, Terasaki PI, Tiercy JM, Trowsdale J (2010). Nomenclature for factors of the HLA system, 2010. Tissue Antigens.

[CR46] McConnell SC, Restaino AC, de Jong JL (2014). Multiple divergent haplotypes express completely distinct sets of class I MHC genes in zebrafish. Immunogenetics.

[CR47] Nonaka MI, Nonaka M (2010). Evolutionary analysis of two classical MHC class I loci of the medaka fish, *Oryzias latipes*: haplotype-specific genomic diversity, locus-specific polymorphisms, and interlocus homogenization. Immunogenetics.

[CR48] NorwegianEnvironmentAgency (2014) Guidelines for stock enhancement for anadromous salmonids. vol M-nummer: 186 M.N.E.Agency. https://www.miljodirektoratet.no/globalassets/publikasjoner/M186/M186.pdf

[CR49] O’Farrell B, Benzie JA, McGinnity P, de Eyto E, Dillane E, Coughlan J, Cross TF (2013). Selection and phylogenetics of salmonid MHC class I: wild brown trout (*Salmo trutta*) differ from a non-native introduced strain. PLoS One.

[CR50] Ono H, Klein D, Vincek V, Figueroa F, O’hUigin C, Tichy H, Klein J (1992). Major histocompatibility complex class II genes of zebrafish. Proc Natl Acad Sci U S A.

[CR51] Rice P, Longden I, Bleasby A (2000). EMBOSS: the European molecular biology open software suite. Trends Genet.

[CR52] Savilammi T (2019). The chromosome-level genome assembly of European grayling reveals aspects of a unique genome evolution process within salmonids. G3 (Bethesda).

[CR53] Shiina T, Dijkstra JM, Shimizu S, Watanabe A, Yanagiya K, Kiryu I, Fujiwara A, Nishida-Umehara C, Kaba Y, Hirono I, Yoshiura Y, Aoki T, Inoko H, Kulski JK, Ototake M (2005). Interchromosomal duplication of major histocompatibility complex class I regions in rainbow trout (*Oncorhynchus mykiss*), a species with a presumably recent tetraploid ancestry. Immunogenetics.

[CR54] Shum BP, Rajalingam R, Magor KE, Azumi K, Carr WH, Dixon B, Stet RJ, Adkison MA, Hedrick RP, Parham P (1999). A divergent non-classical class I gene conserved in salmonids. Immunogenetics.

[CR55] Stabell O (1984). Homing and olfaction in salmonids: a critical review with special reference to the Atlantic salmon. Biol Rev.

[CR56] Star B, Nederbragt AJ, Jentoft S, Grimholt U, Malmstrøm M, Gregers TF, Rounge TB, Paulsen J, Solbakken MH, Sharma A, Wetten OF, Lanzén A, Winer R, Knight J, Vogel JH, Aken B, Andersen O, Lagesen K, Tooming-Klunderud A, Edvardsen RB, Tina KG, Espelund M, Nepal C, Previti C, Karlsen BO, Moum T, Skage M, Berg PR, Gjøen T, Kuhl H, Thorsen J, Malde K, Reinhardt R, du L, Johansen SD, Searle S, Lien S, Nilsen F, Jonassen I, Omholt SW, Stenseth NC, Jakobsen KS (2011). The genome sequence of Atlantic cod reveals a unique immune system. Nature.

[CR57] Stet RJ, Kruiswijk CP, Saeij JP, Wiegertjes GF (1998). Major histocompatibility genes in cyprinid fishes: theory and practice. Immunol Rev.

[CR58] Stet RJ, de Vries B, Mudde K, Hermsen T, van Heerwaarden J, Shum BP, Grimholt U (2002). Unique haplotypes of co-segregating major histocompatibility class II A and class II B alleles in Atlantic salmon (*Salmo salar*) give rise to diverse class II genotypes. Immunogenetics.

[CR59] Sultmann H, Meyer WE, Figueroa F, O’hUigin C, Klein J (1993). Zebrafish Mhc class II alpha chain-encoding genes: polymorphism, expression and function. Immunogenetics.

[CR60] Sultmann H, Mayer WE, Figueroa F, O’Huigin C, Klein J (1994). Organization of Mhc class II B genes in the zebrafish (*Brachydanio rerio*). Genomics.

[CR61] Waterhouse AM, Procter JB, Martin DM, Clamp M, Barton GJ (2009). Jalview Version 2--a multiple sequence alignment editor and analysis workbench. Bioinformatics.

[CR62] Whelan S, Goldman N (2001). A general empirical model of protein evolution derived from multiple protein families using a maximum-likelihood approach. Mol Biol Evol.

