# Correction to: An Illumina approach to MHC typing of Atlantic salmon

**DOI:** 10.1007/s00251-019-01152-7

**Published:** 2019-12-10

**Authors:** Arvind Y. M. Sundaram, Åse Helen Garseth, Giuseppe Maccari, Unni Grimholt

**Affiliations:** 1 Norwegian Veterinary Institute, P.O. Box 750 Sentrum, 0106 Oslo, Norway; 2 Department of Medical Genetics, Oslo University Hospital, 0450 Oslo, Norway; 3 The Pirbright Institute, Woking, UK; 4 Anthony Nolan Research Institute, London, UK

**Correction to: Immunogenetics**

10.1007/s00251-019-01143-8

The article “An Illumina approach to MHC typing of Atlantic salmon”, written by Arvind Y. M. Sundaram, Åse Helen Garseth, Giuseppe Maccari and Unni Grimholt, was originally published electronically on the publisher’s internet portal on 12 November 2019 without open access. 

With the author(s)’ decision to opt for Open Choice the copyright of the article changed on December 2019 to © The Author(s) 2019 and the article is forthwith distributed under a Creative Commons Attribution 4.0 International License (https://creativecommons.org/licenses/by/4.0/), which permits use, sharing, adaptation, distribution and reproduction in any medium or format, as long as you give appropriate credit to the original author(s) and the source, provide a link to the Creative Commons license, and indicate if changes were made.

The original article was corrected.

