# Supplementary material for: An Illumina approach to MHC typing of Atlantic salmon
Source: Immunogenetics. 2019 Nov 12;72(1-2):89–100. doi: 10.1007/s00251-019-01143-8 (PMC6970960; doi:10.1007/s00251-019-01143-8)
Supplement: Supplementary file 1 — New IPD-MHC Fish nomenclature (PDF 725 kb) [file 251_2019_1143_MOESM1_ESM.pdf]

Supplementary file (SF) 1. New and previous nomenclature for the Fish IPD-MHC Database

Table of contents		Page
SF1A	Atlantic salmon (*Salmo salar*) names	1
SF1B	Rainbow trout (*Oncorhynchus mykiss*) names	3

SF1A. Atlantic salmon (*Salmo salar*) names:

IPD Accession	New name	Previous Name	Species
FISH08192	Sasa-DAA*01:01	Sasa-DAA*0101	*Salmo salar*
FISH08193	Sasa-DAA*01:02	Sasa-DAA*0102	*Salmo salar*
FISH08194	Sasa-DAA*02:01	Sasa-DAA*0201	*Salmo salar*
FISH08195	Sasa-DAA*03:01	Sasa-DAA*0301	*Salmo salar*
FISH08196	Sasa-DAA*03:02	Sasa-DAA*0302	*Salmo salar*
FISH08197	Sasa-DAA*03:03:01	Sasa-DAA*030301	*Salmo salar*
FISH08198	Sasa-DAA*03:03:02	Sasa-DAA*030302	*Salmo salar*
FISH08199	Sasa-DAA*03:04	Sasa-DAA*0304	*Salmo salar*
FISH08200	Sasa-DAA*03:05	Sasa-DAA*0305	*Salmo salar*
FISH08201	Sasa-DAA*04:01	Sasa-DAA*0401	*Salmo salar*
FISH08202	Sasa-DAA*05:01	Sasa-DAA*0501	*Salmo salar*
FISH08203	Sasa-DAA*05:02	Sasa-DAA*0502	*Salmo salar*
FISH08204	Sasa-DAA*06:01	Sasa-DAA*0601	*Salmo salar*
FISH08205	Sasa-DAA*07:01	Sasa-DAA*0701	*Salmo salar*
FISH08206	Sasa-DAA*08:01	Sasa-DAA*0801	*Salmo salar*
FISH08207	Sasa-DAA*09:01	Sasa-DAA*0901	*Salmo salar*
FISH08208	Sasa-DAA*10:01	Sasa-DAA*1001	*Salmo salar*
FISH08209	Sasa-DAA*11:01	Sasa-DAA*1101	*Salmo salar*
FISH08210	Sasa-DAA*12:01	Sasa-DAA*1201	*Salmo salar*
FISH08211	Sasa-DAA*12:02	Sasa-DAA*1202	*Salmo salar*
FISH08212	Sasa-DAA*13:01	Sasa-DAA*1301	*Salmo salar*
FISH08213	Sasa-DAA*14:01	Sasa-DAA*1401	*Salmo salar*
FISH08214	Sasa-DAB*01:01	Sasa-DAB*0101	*Salmo salar*
FISH08215	Sasa-DAB*01:02	Sasa-DAB*0102	*Salmo salar*
FISH08216	Sasa-DAB*02:01	Sasa-DAB*0201	*Salmo salar*
FISH08217	Sasa-DAB*02:02	Sasa-DAB*0202	*Salmo salar*
FISH08218	Sasa-DAB*03:01	Sasa-DAB*0301	*Salmo salar*
FISH08219	Sasa-DAB*03:02	Sasa-DAB*0302	*Salmo salar*
FISH08220	Sasa-DAB*03:03	Sasa-DAB*0303	*Salmo salar*
FISH08221	Sasa-DAB*04:01	Sasa-DAB*0401	*Salmo salar*
FISH08222	Sasa-DAB*05:01	Sasa-DAB*0501	*Salmo salar*
FISH08223	Sasa-DAB*06:01	Sasa-DAB*0601	*Salmo salar*
FISH08224	Sasa-DAB*07:01	Sasa-DAB*0701	*Salmo salar*
FISH08225	Sasa-DAB*08:01	Sasa-DAB*0801	*Salmo salar*

FISH08226	Sasa-DAB*08:02	Sasa-DAB*0802	*Salmo salar*
FISH08227	Sasa-DAB*09:01	Sasa-DAB*0901	*Salmo salar*
FISH08228	Sasa-DAB*09:02	Sasa-DAB*0902	*Salmo salar*
FISH08229	Sasa-DAB*10:01	Sasa-DAB*1001	*Salmo salar*
FISH08230	Sasa-DAB*11:01	Sasa-DAB*1101	*Salmo salar*
FISH08231	Sasa-DAB*11:02	Sasa-DAB*1102	*Salmo salar*
FISH08232	Sasa-DAB*12:01	Sasa-DAB*1201	*Salmo salar*
FISH08233	Sasa-DAB*12:02	Sasa-DAB*1202	*Salmo salar*
FISH08234	Sasa-DAB*13:01	Sasa-DAB*1301	*Salmo salar*
FISH08235	Sasa-DAB*13:02	Sasa-DAB*1302	*Salmo salar*
FISH08236	Sasa-DAB*13:03	Sasa-DAB*1303	*Salmo salar*
FISH08237	Sasa-DAB*14:01	Sasa-DAB*1401	*Salmo salar*
FISH08238	Sasa-DAB*15:01	Sasa-DAB*1501	*Salmo salar*
FISH08239	Sasa-DAB*15:02	Sasa-DAB*1502	*Salmo salar*
FISH08240	Sasa-DAB*16:01	Sasa-DAB*1601	*Salmo salar*
FISH08241	Sasa-DAB*17:01	Sasa-DAB*1701	*Salmo salar*
FISH08242	Sasa-DAB*17:02	Sasa-DAB*1702	*Salmo salar*
FISH08243	Sasa-DAB*17:03	Sasa-DAB*1703	*Salmo salar*
FISH08244	Sasa-DAB*18:01	Sasa-DAB*1801	*Salmo salar*
FISH08245	Sasa-DAB*19:01	Sasa-DAB*1901	*Salmo salar*
FISH08246	Sasa-DAB*20:01	Sasa-DAB*2001	*Salmo salar*
FISH08247	Sasa-DAB*21:01	Sasa-DAB*2101	*Salmo salar*
FISH08248	Sasa-DAB*22:01	Sasa-DAB*2201	*Salmo salar*
FISH08249	Sasa-DAB*23:01	Sasa-DAB*2301	*Salmo salar*
FISH08250	Sasa-DAB*23:02	Sasa-DAB*2302	*Salmo salar*
FISH08251	Sasa-DAB*24:01	Sasa-DAB*2401	*Salmo salar*
FISH08252	Sasa-DAB*25:01	Sasa-DAB*2501	*Salmo salar*
FISH08253	Sasa-DAB*26:01	Sasa-DAB*2601	*Salmo salar*
FISH08254	Sasa-DAB*27:01	Sasa-DAB*2701	*Salmo salar*
FISH08255	Sasa-DAB*28:01	Sasa-DAB*2801	*Salmo salar*
FISH08256	Sasa-UBA*01:01	Sasa-UBA*0101	*Salmo salar*
FISH08257	Sasa-UBA*02:01	Sasa-UBA*0201	*Salmo salar*
FISH08258	Sasa-UBA*03:01	Sasa-UBA*0301	*Salmo salar*
FISH08300	Sasa-UBA*03:02	Sasa-UBA*0302	*Salmo salar*
FISH08259	Sasa-UBA*04:01	Sasa-UBA*0401	*Salmo salar*
FISH08260	Sasa-UBA*04:02	Sasa-UBA*0402	*Salmo salar*
FISH08261	Sasa-UBA*05:01	Sasa-UBA*0501	*Salmo salar*
FISH08262	Sasa-UBA*06:01	Sasa-UBA*0601	*Salmo salar*
FISH08263	Sasa-UBA*06:02	Sasa-UBA*0602	*Salmo salar*
FISH08264	Sasa-UBA*06:03	Sasa-UBA*0603	*Salmo salar*
FISH08265	Sasa-UBA*07:01	Sasa-UBA*0701	*Salmo salar*
FISH08266	Sasa-UBA*08:01	Sasa-UBA*0801	*Salmo salar*
FISH08267	Sasa-UBA*09:01	Sasa-UBA*0901	*Salmo salar*

FISH08268	Sasa-UBA*10:01	Sasa-UBA*1001	*Salmo salar*
FISH08269	Sasa-UBA*11:01	Sasa-UBA*1101	*Salmo salar*
FISH08270	Sasa-UBA*12:01	Sasa-UBA*1201	*Salmo salar*
FISH08271	Sasa-UBA*13:01	Sasa-UBA*1301	*Salmo salar*
FISH08272	Sasa-UBA*14:01	Sasa-UBA*1401	*Salmo salar*
FISH08273	Sasa-UBA*15:01	Sasa-UBA*1501	*Salmo salar*
FISH08274	Sasa-UBA*16:01	Sasa-UBA*1601	*Salmo salar*
FISH08275	Sasa-UBA*17:01	Sasa-UBA*1701	*Salmo salar*
FISH08276	Sasa-UBA*18:01	Sasa-UBA*1801	*Salmo salar*
FISH08277	Sasa-UBA*18:02	Sasa-UBA*1802	*Salmo salar*
FISH08278	Sasa-UBA*19:01	Sasa-UBA*1901	*Salmo salar*
FISH08279	Sasa-UBA*20:01	Sasa-UBA*2001	*Salmo salar*
FISH08280	Sasa-UBA*20:02	Sasa-UBA*2002	*Salmo salar*
FISH08281	Sasa-UBA*21:01	Sasa-UBA*2101	*Salmo salar*
FISH08282	Sasa-UBA*22:01	Sasa-UBA*2201	*Salmo salar*
FISH08283	Sasa-UBA*23:01	Sasa-UBA*2301	*Salmo salar*
FISH08284	Sasa-UBA*24:01	Sasa-UBA*2401	*Salmo salar*
FISH08285	Sasa-UBA*24:02	Sasa-UBA*2402	*Salmo salar*
FISH08286	Sasa-UBA*24:03	Sasa-UBA*2403	*Salmo salar*
FISH08287	Sasa-UBA*25:01	Sasa-UBA*2501	*Salmo salar*
FISH08288	Sasa-UBA*26:01	Sasa-UBA*2601	*Salmo salar*
FISH08289	Sasa-UBA*26:02	Sasa-UBA*2602	*Salmo salar*
FISH08290	Sasa-UBA*27:01	Sasa-UBA*2701	*Salmo salar*
FISH08291	Sasa-UBA*28:01	Sasa-UBA*2801	*Salmo salar*
FISH08292	Sasa-UBA*29:01	Sasa-UBA*2901	*Salmo salar*
FISH08293	Sasa-UBA*30:01	Sasa-UBA*3001	*Salmo salar*
FISH08294	Sasa-UBA*31:01	Sasa-UBA*3101	*Salmo salar*
FISH08295	Sasa-UBA*32:01	Sasa-UBA*3201	*Salmo salar*
FISH08296	Sasa-UBA*33:01	Sasa-UBA*3301	*Salmo salar*
FISH08297	Sasa-UBA*34:01	Sasa-UBA*3401	*Salmo salar*
FISH08302	Sasa-UBA*34:02	Sasa-UBA*3402	*Salmo salar*
FISH08303	Sasa-UBA*35:01	Sasa-UBA*3501	*Salmo salar*
FISH08299	Sasa-UBA*36:01	Sasa-UBA*3601	*Salmo salar*
FISH08301	Sasa-UBA*37:01	Sasa-UBA*3701	*Salmo salar*
FISH08298	Sasa-UBA*38:01	Sasa-UBA*3801	*Salmo salar*

SF1B. New Rainbow trout (*Oncorhynchus mykiss*) names:

IPD Accession	New name	Previous name	Species
FISH08119	Onmy-DAA*01:01	DAA*0101	*Oncorhynchus mykiss*
FISH08120	Onmy-DAA*02:01	DAA*0201	*Oncorhynchus mykiss*
FISH08121	Onmy-DAA*03:01	DAA*0301	*Oncorhynchus mykiss*
FISH08122	Onmy-DAB*01:01	DAB*0101	*Oncorhynchus mykiss*

FISH08123	Onmy-DAB*02:01	DAB*0201	*Oncorhynchus mykiss*
FISH08124	Onmy-DAB*03:01:01	DAB*030101	*Oncorhynchus mykiss*
FISH08125	Onmy-DAB*03:01:02	DAB*030102	*Oncorhynchus mykiss*
FISH08126	Onmy-DAB*04:01	DAB*0401	*Oncorhynchus mykiss*
FISH08127	Onmy-DAB*05:01:01	DAB*050101	*Oncorhynchus mykiss*
FISH08128	Onmy-DAB*05:01:02	DAB*050102	*Oncorhynchus mykiss*
FISH08129	Onmy-DAB*05:02	DAB*0502	*Oncorhynchus mykiss*
FISH08130	Onmy-DAB*06:01:01	DAB*060101	*Oncorhynchus mykiss*
FISH08131	Onmy-DAB*06:01:02	DAB*060102	*Oncorhynchus mykiss*
FISH08132	Onmy-DAB*07:01	DAB*0701	*Oncorhynchus mykiss*
FISH08133	Onmy-DAB*08:01	DAB*0801	*Oncorhynchus mykiss*
FISH08134	Onmy-DAB*09:01	DAB*0901	*Oncorhynchus mykiss*
FISH08135	Onmy-DAB*10:01	DAB*1001	*Oncorhynchus mykiss*
FISH08136	Onmy-DAB*11:01	DAB*1101	*Oncorhynchus mykiss*
FISH08137	Onmy-DAB*12:01	DAB*1201	*Oncorhynchus mykiss*
FISH08138	Onmy-DAB*13:01	DAB*1301	*Oncorhynchus mykiss*
FISH08139	Onmy-DAB*14:01	DAB*1401	*Oncorhynchus mykiss*
FISH08140	Onmy-DAB*15:01	DAB*1501	*Oncorhynchus mykiss*
FISH08141	Onmy-DAB*16:01	DAB*1601	*Oncorhynchus mykiss*
FISH08142	Onmy-DAB*16:02	DAB*1602	*Oncorhynchus mykiss*
FISH08143	Onmy-DAB*17:01	DAB*1701	*Oncorhynchus mykiss*
FISH08144	Onmy-UBA*01:01:01	UBA*010101	*Oncorhynchus mykiss*
FISH08145	Onmy-UBA*01:01:02	UBA*010102	*Oncorhynchus mykiss*
FISH08146	Onmy-UBA*01:01:03	UBA*010103	*Oncorhynchus mykiss*
FISH08147	Onmy-UBA*01:02	UBA*0102	*Oncorhynchus mykiss*
FISH08148	Onmy-UBA*01:03	UBA*0103	*Oncorhynchus mykiss*
FISH08149	Onmy-UBA*01:04	UBA*0104	*Oncorhynchus mykiss*
FISH08150	Onmy-UBA*02:01	UBA*0201	*Oncorhynchus mykiss*
FISH08151	Onmy-UBA*02:02	UBA*0202	*Oncorhynchus mykiss*
FISH08152	Onmy-UBA*03:01	UBA*0301	*Oncorhynchus mykiss*
FISH08153	Onmy-UBA*04:01:01	UBA*040101	*Oncorhynchus mykiss*
FISH08154	Onmy-UBA*04:01:02	UBA*040102	*Oncorhynchus mykiss*
FISH08155	Onmy-UBA*05:01	UBA*0501	*Oncorhynchus mykiss*
FISH08156	Onmy-UBA*05:02	UBA*0502	*Oncorhynchus mykiss*
FISH08157	Onmy-UBA*05:03	UBA*0503	*Oncorhynchus mykiss*
FISH08158	Onmy-UBA*06:01	UBA*0601	*Oncorhynchus mykiss*
FISH08159	Onmy-UBA*07:01	UBA*0701	*Oncorhynchus mykiss*
FISH08160	Onmy-UBA*08:01	UBA*0801	*Oncorhynchus mykiss*
FISH08161	Onmy-UBA*09:01	UBA*0901	*Oncorhynchus mykiss*
FISH08162	Onmy-UBA*09:02	UBA*0902	*Oncorhynchus mykiss*
FISH08163	Onmy-UBA*10:01	UBA*1001	*Oncorhynchus mykiss*
FISH08164	Onmy-UBA*11:01	UBA*1101	*Oncorhynchus mykiss*
FISH08165	Onmy-UBA*12:01	UBA*1201	*Oncorhynchus mykiss*

FISH08166	Onmy-UBA*13:01	UBA*1301	*Oncorhynchus mykiss*
FISH08167	Onmy-UBA*14:01	UBA*1401	*Oncorhynchus mykiss*
FISH08168	Onmy-UBA*15:01	UBA*1501	*Oncorhynchus mykiss*
FISH08169	Onmy-UBA*15:01:01	UBA*150101	*Oncorhynchus mykiss*
FISH08170	Onmy-UBA*15:01:02	UBA*150102	*Oncorhynchus mykiss*
FISH08171	Onmy-UBA*15:02	UBA*1502	*Oncorhynchus mykiss*
FISH08172	Onmy-UBA*16:01	UBA*1601	*Oncorhynchus mykiss*
FISH08173	Onmy-UBA*17:01	UBA*1701	*Oncorhynchus mykiss*
FISH08174	Onmy-UBA*18:01	UBA*1801	*Oncorhynchus mykiss*
FISH08175	Onmy-UBA*18:02	UBA*1802	*Oncorhynchus mykiss*
FISH08176	Onmy-UBA*19:01	UBA*1901	*Oncorhynchus mykiss*
FISH08177	Onmy-UBA*20:01	UBA*2001	*Oncorhynchus mykiss*
FISH08178	Onmy-UBA*21:01	UBA*2101	*Oncorhynchus mykiss*
FISH08179	Onmy-UBA*22:01	UBA*2201	*Oncorhynchus mykiss*
FISH08180	Onmy-UBA*23:01	UBA*2301	*Oncorhynchus mykiss*
FISH08181	Onmy-UBA*24:01	UBA*2401	*Oncorhynchus mykiss*
FISH08182	Onmy-UBA*25:01	UBA*2501	*Oncorhynchus mykiss*
FISH08183	Onmy-UBA*26:01	UBA*2601	*Oncorhynchus mykiss*
FISH08184	Onmy-UBA*27:01:01	UBA*270101	*Oncorhynchus mykiss*
FISH08185	Onmy-UBA*27:01:02	UBA*270102	*Oncorhynchus mykiss*
FISH08186	Onmy-UBA*27:01:03	UBA*270103	*Oncorhynchus mykiss*
FISH08187	Onmy-UBA*28:01	UBA*2801	*Oncorhynchus mykiss*
FISH08188	Onmy-UBA*29:01	UBA*2901	*Oncorhynchus mykiss*
FISH08189	Onmy-UBA*30:01	UBA*3001	*Oncorhynchus mykiss*
FISH08190	Onmy-UBA*31:01	UBA*3101	*Oncorhynchus mykiss*
FISH08191	Onmy-UBA*32:01	UBA*3201	*Oncorhynchus mykiss*
