# Supplementary material for: An Illumina approach to MHC typing of Atlantic salmon
Source: Immunogenetics. 2019 Nov 12;72(1-2):89–100. doi: 10.1007/s00251-019-01143-8 (PMC6970960; doi:10.1007/s00251-019-01143-8)
Supplement: Supplementary file 2 — Illumina Statistics (PDF 518 kb) [file 251_2019_1143_MOESM2_ESM.pdf]

Supplementary file 2 (SF2) legend: Table SF2A shows the number of sequenced reads, cleaned reads and number of demultiplexed reads for each primer pair used for all animals. The SF2B table shows the total number of usable FLASHed reads for each of the groups. Table SF2C provides the information from collapsing the FLASHed reads using fastx_collapser and provides the abundance of the top five unique full-length amplified reads.

SF2A. Number of sequenced and clean demultiplexed reads

Animal	number of raw_reads	number of clean_reads	percentage of clean_reads	DAA	DAB(1/2)*	UBA(1/2)*	number of clean reads demultiplexed	percentage of clean reads demultiplexed
**AS1**	1 274 830	1 074 939	84,32 %	259 515	442 196	167 749	869 460	80,88%
**AS2**	1 381 710	1 175 102	85,05 %	228 618	310 003	444 075	982 696	83,63%
**AS3**	655 886	560 510	85,46 %	107 425	245 052	121 123	473 600	84,49%
**AS5**	922 452	809 459	87,75 %	97 336	168 025	418 500	683 861	84,48%
**AS6**	815 238	687 238	84,30 %	136 911	350 616	107 258	594 785	86,55%
**AS7**	1 241 121	1 068 570	86,10 %	222 453	469 997	165 882	858 332	80,33%
**AS8**	1 288 043	1 082 609	84,05 %	157 642	379 079	376 031	912 752	84,31%
**AS9**	1 128 638	937 474	83,06 %	182 598	345 572	269 411	797 581	85,08%
**AS10**	1 335 300	1 094 359	81,96 %	259 547	361 149	302 651	923 347	84,37%

* Either forward primer 1 or 2 was used

SF2B. Number of FLASHed reads

Animal	DAA FLASHed	DAB* FLASHed	UBA* FLASHed	number of clean reads used	percentage of clean reads used
**AS1**	255 485	385 794	120 150	761 429	70,83%
**AS2**	223 915	207 379	395 469	826 763	70,36%
**AS3**	104 573	84 258	104 715	293 546	52,37%
**AS5**	95 531	94 978	186 847	377 356	46,62%
**AS6**	132 825	68 395	89 116	290 336	42,25%
**AS7**	217 156	429 139	131 219	777 514	72,76%
**AS8**	154 498	354 960	250 708	760 166	70,22%
**AS9**	178 584	121 816	238 519	538 919	57,49%
**AS10**	252 011	232 974	269 763	754 748	68,97%

* Either forward primer 1 or 2 was used

SF2C. Number of unique and FLASHed reads per gene per animal

DAA			DAB*			UBA*		
AS1 (number of FLASHed reads: 255,485)	number of unique reads	number of FLASHed reads	AS1 (number of FLASHed reads: 385,794)	number of unique reads	number of FLASHed reads	AS1 (number of FLASHed reads: 120,150)	number of unique reads	number of FLASHed reads
AS1_DAA_s1	39 861	15,60 %	AS1_DAB2_s1	63 791	16,53 %	AS1_UBA1_s1	30 927	25,74 %
AS1_DAA_s2	37 247	14,58 %	AS1_DAB2_s2	20 621	5,35 %	AS1_UBA1_s2	203	0,17 %
AS1_DAA_s3	2 785	1,09 %	AS1_DAB2_s3	5 382	1,40 %	AS1_UBA1_s3	169	0,14 %
AS1_DAA_s4	2 703	1,06 %	AS1_DAB2_s4	4 227	1,10 %	AS1_UBA1_s4	161	0,13 %
AS1_DAA_s5	2 591	1,01 %	AS1_DAB2_s5	3 463	0,90 %	AS1_UBA1_s5	153	0,13 %
AS2 (number of FLASHed reads: 223,915)	number of unique reads	number of FLASHed reads	AS2 (number of FLASHed reads: 207,379)	number of unique reads	number of FLASHed reads	AS2 (number of FLASHed reads: 395,469)	number of unique reads	number of FLASHed reads
AS2_DAA_s1	29 496	13,17 %	AS2_DAB1_s1	30 852	14,88 %	AS2_UBA1_s1	46 437	11,74 %
AS2_DAA_s2	28 281	12,63 %	AS2_DAB1_s2	17 876	8,62 %	AS2_UBA1_s2	33 647	8,51 %
AS2_DAA_s3	4 098	1,83 %	AS2_DAB1_s3	3 147	1,52 %	AS2_UBA1_s3	8 559	2,16 %
AS2_DAA_s4	4 026	1,80 %	AS2_DAB1_s4	2 822	1,36 %	AS2_UBA1_s4	7 940	2,01 %
AS2_DAA_s5	3 783	1,69 %	AS2_DAB1_s5	1 143	0,55 %	AS2_UBA1_s5	4 195	1,06 %
AS3 (number of FLASHed reads: 104,573)	number of unique reads	number of FLASHed reads	AS3 (number of FLASHed reads: 84,258)	number of unique reads	number of FLASHed reads	AS3 (number of FLASHed reads: 104,715)	number of unique reads	number of FLASHed reads
AS3_DAA_s1	14 569	13,93 %	AS3_DAB1_s1	10 179	12,08 %	AS3_UBA1_s1	13 465	12,86 %
AS3_DAA_s2	12 123	11,59 %	AS3_DAB1_s2	7 127	8,46 %	AS3_UBA1_s2	12 935	12,35 %
AS3_DAA_s3	2 271	2,17 %	AS3_DAB1_s3	1 585	1,88 %	AS3_UBA1_s3	609	0,58 %
AS3_DAA_s4	2 119	2,03 %	AS3_DAB1_s4	1 553	1,84 %	AS3_UBA1_s4	609	0,58 %
AS3_DAA_s5	888	0,85 %	AS3_DAB1_s5	1 509	1,79 %	AS3_UBA1_s5	288	0,28 %

AS5 (number of FLASHed reads: 95,531)	number of unique reads	number of FLASHed reads	AS5 (number of FLASHed reads: 94,978)	number of unique reads	number of FLASHed reads	AS5 (number of FLASHed reads: 186,847)	number of unique reads	number of FLASHed reads
AS5_DAA_s1	38 290	40,08 %	AS5_DAB2_s1	11 545	12,16 %	AS5_UBA2_s1	22 247	11,91 %
AS5_DAA_s2	181	0,19 %	AS5_DAB2_s2	9 933	10,46 %	AS5_UBA2_s2	20 661	11,06 %
AS5_DAA_s3	176	0,18 %	AS5_DAB2_s3	1 500	1,58 %	AS5_UBA2_s3	576	0,31 %
AS5_DAA_s4	172	0,18 %	AS5_DAB2_s4	1 433	1,51 %	AS5_UBA2_s4	489	0,26 %
AS5_DAA_s5	162	0,17 %	AS5_DAB2_s5	994	1,05 %	AS5_UBA2_s5	480	0,26 %
**AS6 (number of FLASHed reads: 132,825)**	**number of unique reads**	**number of FLASHed reads**	**AS6 (number of FLASHed reads: 68,395)**	**number of unique reads**	**number of FLASHed reads**	**AS6 (number of FLASHed reads: 89,116)**	**number of unique reads**	**number of FLASHed reads**
AS6_DAA_s1	21 935	16,51 %	AS6_DAB1_s1	10 396	15,20 %	AS6_UBA1_s1	18 686	20,97 %
AS6_DAA_s2	19 807	14,91 %	AS6_DAB1_s2	9 420	13,77 %	AS6_UBA1_s2	3 956	4,44 %
AS6_DAA_s3	2 360	1,78 %	AS6_DAB1_s3	1 464	2,14 %	AS6_UBA1_s3	399	0,45 %
AS6_DAA_s4	2 123	1,60 %	AS6_DAB1_s4	1 282	1,87 %	AS6_UBA1_s4	297	0,33 %
AS6_DAA_s5	393	0,30 %	AS6_DAB1_s5	82	0,12 %	AS6_UBA1_s5	287	0,32 %
**AS7 (number of FLASHed reads: 217,156)**	**number of unique reads**	**number of FLASHed reads**	**AS7 (number of FLASHed reads: 429,139)**	**number of unique reads**	**number of FLASHed reads**	**AS7 (number of FLASHed reads: 131,219)**	**number of unique reads**	**number of FLASHed reads**
AS7_DAA_s1	31 665	14,58 %	AS7_DAB2_s1	71 872	16,75 %	AS7_UBA1_s1	25 193	19,20 %
AS7_DAA_s2	29 058	13,38 %	AS7_DAB2_s2	24 758	5,77 %	AS7_UBA1_s2	6 454	4,92 %
AS7_DAA_s3	2 271	1,05 %	AS7_DAB2_s3	7 352	1,71 %	AS7_UBA1_s3	481	0,37 %
AS7_DAA_s4	2 177	1,00 %	AS7_DAB2_s4	5 908	1,38 %	AS7_UBA1_s4	289	0,22 %
AS7_DAA_s5	2 085	0,96 %	AS7_DAB2_s5	1 869	0,44 %	AS7_UBA1_s5	171	0,13 %

AS8 (number of FLASHed reads: 154,498)	number of unique reads	number of FLASHed reads	AS8 (number of FLASHed reads: 354,960)	number of unique reads	number of FLASHed reads	AS8 (number of FLASHed reads: 250,708)	number of unique reads	number of FLASHed reads
AS8_DAA_s1	25 961	16,80 %	AS8_DAB2_s1	41 329	11,64 %	AS8_UBA1_s1	30 959	12,35 %
AS8_DAA_s2	22 389	14,49 %	AS8_DAB2_s2	29 683	8,36 %	AS8_UBA1_s2	24 959	9,96 %
AS8_DAA_s3	1 672	1,08 %	AS8_DAB2_s3	7 380	2,08 %	AS8_UBA1_s3	2 725	1,09 %
AS8_DAA_s4	1 497	0,97 %	AS8_DAB2_s4	7 176	2,02 %	AS8_UBA1_s4	2 424	0,97 %
AS8_DAA_s5	876	0,57 %	AS8_DAB2_s5	5 682	1,60 %	AS8_UBA1_s5	1 597	0,64 %
AS9 (number of FLASHed reads: 178,584)	number of unique reads	number of FLASHed reads	AS9 (number of FLASHed reads: 121,816)	number of unique reads	number of FLASHed reads	AS9 (number of FLASHed reads: 238,519)	number of unique reads	number of FLASHed reads
AS9_DAA_s1	27 650	15,48 %	AS9_DAB1_s1	16 301	13,38 %	AS9_UBA1_s1	20 357	8,53 %
AS9_DAA_s2	22 549	12,63 %	AS9_DAB1_s2	10 525	8,64 %	AS9_UBA1_s2	14 112	5,92 %
AS9_DAA_s3	2 292	1,28 %	AS9_DAB1_s3	2 669	2,19 %	AS9_UBA1_s3	3 551	1,49 %
AS9_DAA_s4	2 277	1,28 %	AS9_DAB1_s4	2 356	1,93 %	AS9_UBA1_s4	3 146	1,32 %
AS9_DAA_s5	1 054	0,59 %	AS9_DAB1_s5	2 051	1,68 %	AS9_UBA1_s5	2 549	1,07 %
AS10 (number of FLASHed reads: 252,011)	number of unique reads	number of FLASHed reads	AS10 (number of FLASHed reads: 232,974)	number of unique reads	number of FLASHed reads	AS10 (number of FLASHed reads: 269,763)	number of unique reads	number of FLASHed reads
AS10_DAA_s1	90 816	36,04 %	AS10_DAB1_s1	74 482	31,97 %	AS10_UBA1_s1	71 332	26,44 %
AS10_DAA_s2	422	0,17 %	AS10_DAB1_s2	380	0,16 %	AS10_UBA1_s2	448	0,17 %
AS10_DAA_s3	419	0,17 %	AS10_DAB1_s3	353	0,15 %	AS10_UBA1_s3	331	0,12 %
AS10_DAA_s4	410	0,16 %	AS10_DAB1_s4	342	0,15 %	AS10_UBA1_s4	325	0,12 %
AS10_DAA_s5	407	0,16 %	AS10_DAB1_s5	337	0,14 %	AS10_UBA1_s5	322	0,12 %
