# Supplementary material for: An Illumina approach to MHC typing of Atlantic salmon
Source: Immunogenetics. 2019 Nov 12;72(1-2):89–100. doi: 10.1007/s00251-019-01143-8 (PMC6970960; doi:10.1007/s00251-019-01143-8)
Supplement: Supplementary file 3 — MHC sequences (PDF 589 kb) [file 251_2019_1143_MOESM3_ESM.pdf]

Supplementary file (SF) 3. MHC class I and class II sequences identified in this study

SF3A	MHC nucleotide sequences	Page
	MHC class II alpha (DAA) nucleotide sequences	1
	MHC class II beta (DAB) nucleotide sequences	7
	MHC class I alpha (UBA) nucleotide sequences	14
SF3B	Deduced MHC amino acid sequences	21
	Deduced MHC class II alpha (DAA) amino acid sequences	21
	Deduced MHC class II beta (DAB) amino acid sequences	24
	Deduced MHC class I alpha (UBA) amino acid sequences	27

SF3A. Allelic MHC nucleotide sequences

Nucleotide sequences for the MHC sequences identified in this study using the custom library preparation and Illumina MiSeq sequencing. These were further processed using the custom scripts. Individual sequence names refer to the animal (Atlantic salmon animal number one is AS1), the MHC class (e.g. MHC class II alpha is DAA), the sequence number based on total number of reads (e.g. sequence one is s1) and the number of collapsed reads for each sequence. The top five collapsed sequences are shown for each gene in each animal.

MHC class II alpha (DAA) nucleotide sequences:

```
>AS1_DAA_s1_39861
CACAAAGTTCTGCATATTGATTTAGTTATTAGTGGATGCAGTGATTCAGATGGAGTGGAC
ATGTATGGACTGGATGGGGAAGAGATGTGGTACGCAGACTTCAACAAGGGGGAGGGAGTG
ATGCCACTGCCTCCGTTTGCAGATCCATTTACCTACCCTGGAGCTTATGAACAGGCTGTA
GGTAACCAGGGGGTATGCAAAGCAAACCTGGCCGTAAACATAAAAGCTTACAAGAACCCA
GAAGAGAAAATAGCCCCTCCTCACAGCAGCATCTACCCCCGGGACGATGTGGACCTGGGG
GTGGAGAACACCCTCATCTGCCATGTCAGCGGGTTCTACCCTGCACCTGTCCGAGTCAGG
TGGACCAGGAACAATCAGAACCTGACAGAGGGAGTACGTCTCAGCACCCCC
>AS1_DAA_s2_37247
CACAAAGTTCTGCATATTGATTTACATATTATTGGATGCAGTGATTCAGATGGAGTGGAC
ATGTATGGACTGGATGGGGAAGAGATGTGGTACGCAGACTTCAACAAGGGGGAGGGAGTG
GTGGCCCTGCCTCCGTTTGCAGATCCATTTACCTTCCCTGGATTTTATGAAGGGGCTGTA
GGTAACCAGGGGGTATGCAAAGCAAACCTGGCCGTAAACATAAAAGCTTACAAGAACCCA
GAAGAGAAAATAGACCCTCCTCACAGCAGCATCTACCCCCGGGACGATGTGGACCTGGGG
GTGGAGAACACCCTCATCTGCCATGTCAGCGGGTTCTTCCCTGCACCTGTCCGAGTCAGG
TGGACCAGGAACAATCAGAACCTGACAGAGGGAGTACGTCTCAGCACCCCC
>AS1_DAA_s3_2785
CACAAAGTTCTGCATATTGATTTACATATTATTGGATGCAGTGATTCAGATGGAGTGGAC
ATGTATGGACTGGATGGGGAAGAGATGTGGTACGCAGACTTCAACAAGGGGGAGGGAGTG
GTGGCCCTGCCTCCGTTTGCAGATCCATTTACCTTCCCTGGATTTTATGAAGGGGCTGTA
GGTAACCAGGGGGTATGCAAAGCAAACCTGGCCGTAAACATAAAAGCTTACAAGAACCCA
GAAGAGAAAATAGACCCTCCTCACAGCAGCATCTACCCCCGGGACGATGTGGACCTGGGG
GTGGAGAACACCCTCATCTGCCATGTCAGCGGGTTCTACCCTGCACCTGTCCGAGTCAGG
TGGACCAGGAACAATCAGAACCTGACAGAGGGAGTACGTCTCAGCACCCCC
>AS1_DAA_s4_2703
CACAAAGTTCTGCATATTGATTTAGTTATTAGTGGATGCAGTGATTCAGATGGAGTGGAC
ATGTATGGACTGGATGGGGAAGAGATGTGGTACGCAGACTTCAACAAGGGGGAGGGAGTG
ATGCCACTGCCTCCGTTTGCAGATCCATTTACCTACCCTGGAGCTTATGAACAGGCTGTA
GGTAACCAGGGGGTATGCAAAGCAAACCTGGCCGTAAACATAAAAGCTTACAAGAACCCA
GAAGAGAAAATAGACCCTCCTCACAGCAGCATCTACCCCCGGGACGATGTGGACCTGGGG
GTGGAGAACACCCTCATCTGCCATGTCAGCGGGTTCTTCCCTGCACCTGTCCGAGTCAGG
TGGACCAGGAACAATCAGAACCTGACAGAGGGAGTACGTCTCAGCACCCCC
>AS1_DAA_s5_2591
CACAAAGTTCTGCATATTGATTTACATATTATTGGATGCAGTGATTCAGATGGAGTGGAC
```

ATGTATGGACTGGATGGGGAAGAGATGTGGTACGCAGACTTCAACAAGGGGGAGGGAGTG
ATGCCACTGCCTCCGTTTGCAGATCCATTTACCTACCCTGGAGCTTATGAACAGGCTGTA
GGTAACCAGGGGGTATGCAAAGCAAACCTGGCCGTAAACATAAAAGCTTACAAGAACCCA
GAAGAGAAAATAGCCCCTCCTCACAGCAGCATCTACCCCCGGGACGATGTGGACCTGGGG
GTGGAGAACACCCTCATCTGCCATGTCAGCGGGTTCTACCCTGCACCTGTCCGAGTCAGG
TGGACCAGGAACAATCAGAACCTGACAGAGGGAGTACGTCTCAGCACCCCC
>AS2_DAA_s1_29496
CACAAAGTTCTGCATATTGATTTAGTTATTAGTGGATGCAGTGATTCAGATGGAGTGGAC
ATGTATGGACTGGATGGGGAAGAGATGTGGTACGCAGACTTCAACAAGGGGGAGGGAGTG
ATGCCACTGCCTCCGTTTGCAGATCCATTTACCTACCCTGGAGCTTATGAACAGGCTGTA
GGTAACCAGGGGGTATGCAAAGCAAACCTGGCCGTAAACATAAAAGCTTACAAGAACCCA
GAAGAGAAAATAGCCCCTCCTCACAGCAGCATCTACCCCCGGGACGATGTGGACCTGGGG
GTGGAGAACACCCTCATCTGCCATGTCAGCGGGTTCTACCCTGCACCTGTCCGAGTCAGG
TGGACCAGGAACAATCAGAACCTGACAGAGGGAGTACGTCTCAGCACCCCC
>AS2_DAA_s2_28281
CACAAAGTTCTGCATATTGATTTAGCTATTACTGGATGCAGTGATTCAGATGGACTGGAC
ATGTATGGACTGGATGGGGAAGAGATGTGGTACGCAGACTTCAACAAGGGGGAGGGAGTG
ATGCCACTGCCTCCGTTTGCAGATCCATTTACCTACCCTGGAGCTTATGAAGGGGCTGTA
GGTAACCAGGGGATATGCAAAGCAAACCTGGCCACATGTATAAAAGCTTACAAGAACCCA
GAAGAGAAAATAGCCCCTCCTCACAGCAGCATCTACCCCCGGGACGATGTGGACCTGGGG
GTGGAGAACACCCTCATCTGCCATGTCAGCGGGTTCCACCCTGCACCTGTCCGAGTCAGG
TGGACCAGGAACAATCAGAACCTGACAGAGGGAGTACGTCTCAGCACCCCC
>AS2_DAA_s3_4098
CACAAAGTTCTGCATATTGATTTAGTTATTAGTGGATGCAGTGATTCAGATGGAGTGGAC
ATGTATGGACTGGATGGGGAAGAGATGTGGTACGCAGACTTCAACAAGGGGGAGGGAGTG
ATGCCACTGCCTCCGTTTGCAGATCCATTTACCTACCCTGGAGCTTATGAACAGGCTGTA
GGTAACCAGGGGGTATGCAAAGCAAACCTGGCCGTAAACATAAAAGCTTACAAGAACCCA
GAAGAGAAAATAGCCCCTCCTCACAGCAGCATCTACCCCCGGGACGATGTGGACCTGGGG
GTGGAGAACACCCTCATCTGCCATGTCAGCGGGTTCCACCCTGCACCTGTCCGAGTCAGG
TGGACCAGGAACAATCAGAACCTGACAGAGGGAGTACGTCTCAGCACCCCC
>AS2_DAA_s4_4026
CACAAAGTTCTGCATATTGATTTAGCTATTACTGGATGCAGTGATTCAGATGGACTGGAC
ATGTATGGACTGGATGGGGAAGAGATGTGGTACGCAGACTTCAACAAGGGGGAGGGAGTG
ATGCCACTGCCTCCGTTTGCAGATCCATTTACCTACCCTGGAGCTTATGAAGGGGCTGTA
GGTAACCAGGGGATATGCAAAGCAAACCTGGCCACATGTATAAAAGCTTACAAGAACCCA
GAAGAGAAAATAGCCCCTCCTCACAGCAGCATCTACCCCCGGGACGATGTGGACCTGGGG
GTGGAGAACACCCTCATCTGCCATGTCAGCGGGTTCTACCCTGCACCTGTCCGAGTCAGG
TGGACCAGGAACAATCAGAACCTGACAGAGGGAGTACGTCTCAGCACCCCC
>AS2_DAA_s5_3783
CACAAAGTTCTGCATATTGATTTAGTTATTAGTGGATGCAGTGATTCAGATGGAGTGGAC
ATGTATGGACTGGATGGGGAAGAGATGTGGTACGCAGACTTCAACAAGGGGGAGGGAGTG
ATGCCACTGCCTCCGTTTGCAGATCCATTTACCTACCCTGGAGCTTATGAAGGGGCTGTA
GGTAACCAGGGGATATGCAAAGCAAACCTGGCCACATGTATAAAAGCTTACAAGAACCCA
GAAGAGAAAATAGCCCCTCCTCACAGCAGCATCTACCCCCGGGACGATGTGGACCTGGGG
GTGGAGAACACCCTCATCTGCCATGTCAGCGGGTTCCACCCTGCACCTGTCCGAGTCAGG
TGGACCAGGAACAATCAGAACCTGACAGAGGGAGTACGTCTCAGCACCCCC
>AS3_DAA_s1_14569
CACAAAGTTCTGCATATTGATTTAGTTATTACTGGATGCAGTGATTCAGATGGACTGGAC
ATGTATGGACTGGATGGGGAAGAGATGTGGTACGCAGACTTCAACAAGCAGGAGGGAGTG
GTGGCCCTGCCTCCGTTTGCAGATCCATTTACCTTCCCTGGATTTTATGAACAGGCTGTA
GGTAACCAGGGGGTATGCAAAGGAAACCTGGCCAAATGTATAAAAGCTTACAAGAACCCA
GAAGAGAAAATAGACCCTCCTCACAGCAGCATCTACCCCCGGGACGATGTGGACCTGGGG
GTGGAGAACACCCTCATCTGCCATGTCAGCGGGTTCTTCCCTGCACCTGTCCGAGTCAGG
TGGACCAGGAACAATCAGAACCTGACAGAGGGAGTACGTCTCAGCACCCCC
>AS3_DAA_s2_12123
CACAAAGTTCTGCATATTGATTTAGTTATTACTGGATGCAGTGATTCAGATGGAGTGGAC
ATGTATGGACTGGATGGGGAAGAGATGTGGTACGCAGACTTCAACAAGGGGGAGGGAGTG
GTGGCCCTGCCTCCGTTTGCAGATCCATTTACCTTCCATGGAGCTTATGAAGGGGCTGTA
GGTAACCAGGGGGTATGCAAAGCAAACCTGGCCGTAAACATAAAAGCTTACAAGAACCCA
GAAGAGAAAATAGACCCTCCTCACAGCAGCATCTACCCCCGGGACGATGTGGACCTGGGG
GTGGAGAACACCCTCATCTGCCATGTCAGCGGGTTCCACCCTGCACCTGTCCGAGTCAGG

TGGACCAGGAACAATCAGAACCTGACAGAGGGAGTACGTCTCAGCACCCCC
>AS3_DAA_s3_2271
CACAAAGTTCTGCATATTGATTTAGTTATTACTGGATGCAGTGATTCAGATGGACTGGAC
ATGTATGGACTGGATGGGGAAGAGATGTGGTACGCAGACTTCAACAAGCAGGAGGGAGTG
GTGGCCCTGCCTCCGTTTGCAGATCCATTTACCTTCCCTGGATTTTATGAACAGGCTGTA
GGTAACCAGGGGGTATGCAAAGGAAACCTGGCCAAATGTATAAAAGCTTACAAGAACCCA
GAAGAGAAAATAGACCCTCCTCACAGCAGCATCTACCCCCGGGACGATGTGGACCTGGGG
GTGGAGAACACCCTCATCTGCCATGTCAGCGGGTTCCACCCTGCACCTGTCCGAGTCAGG
TGGACCAGGAACAATCAGAACCTGACAGAGGGAGTACGTCTCAGCACCCCC
>AS3_DAA_s4_2119
CACAAAGTTCTGCATATTGATTTAGTTATTACTGGATGCAGTGATTCAGATGGAGTGGAC
ATGTATGGACTGGATGGGGAAGAGATGTGGTACGCAGACTTCAACAAGGGGGAGGGAGTG
GTGGCCCTGCCTCCGTTTGCAGATCCATTTACCTTCCATGGAGCTTATGAAGGGGCTGTA
GGTAACCAGGGGGTATGCAAAGCAAACCTGGCCGTAAACATAAAAGCTTACAAGAACCCA
GAAGAGAAAATAGACCCTCCTCACAGCAGCATCTACCCCCGGGACGATGTGGACCTGGGG
GTGGAGAACACCCTCATCTGCCATGTCAGCGGGTTCTTCCCTGCACCTGTCCGAGTCAGG
TGGACCAGGAACAATCAGAACCTGACAGAGGGAGTACGTCTCAGCACCCCC
>AS3_DAA_s5_888
CACAAAGTTCTGCATATTGATTTAGTTATTACTGGATGCAGTGATTCAGATGGACTGGAC
ATGTATGGACTGGATGGGGAAGAGATGTGGTACGCAGACTTCAACAAGGGGGAGGGAGTG
GTGGCCCTGCCTCCGTTTGCAGATCCATTTACCTTCCATGGAGCTTATGAAGGGGCTGTA
GGTAACCAGGGGGTATGCAAAGCAAACCTGGCCGTAAACATAAAAGCTTACAAGAACCCA
GAAGAGAAAATAGACCCTCCTCACAGCAGCATCTACCCCCGGGACGATGTGGACCTGGGG
GTGGAGAACACCCTCATCTGCCATGTCAGCGGGTTCCACCCTGCACCTGTCCGAGTCAGG
TGGACCAGGAACAATCAGAACCTGACAGAGGGAGTACGTCTCAGCACCCCC
>AS5_DAA_s1_38290
CACAAAGTTCTGCATATTGATTTAGTTATTAGTGGATGCAGTGATTCAGATGGAGTGGAC
ATGTATGGACTGGATGGGGAAGAGATGTGGTACGCAGACTTCAACAAGGGGGAGGGAGTG
ATGCCACTGCCTCCGTTTGCAGATCCATTTACCTACCCTGGAGCTTATGAACAGGCTGTA
GGTAACCAGGGGGTATGCAAAGCAAACCTGGCCGTAAACATAAAAGCTTACAAGAACCCA
GAAGAGAAAATAGCCCCTCCTCACAGCAGCATCTACCCCCGGGACGATGTGGACCTGGGG
GTGGAGAACACCCTCATCTGCCATGTCAGCGGGTTCTACCCTGCACCTGTCCGAGTCAGG
TGGACCAGGAACAATCAGAACCTGACAGAGGGAGTACGTCTCAGCACCCCC
>AS5_DAA_s2_181
CACAAAGTTCTGCATATTGATTTAGTTATTAGTGGATGCAGTGATTCAGATGGAGTGGAC
ATGTATGGACTGGATGGGGAAGGGATGTGGTACGCAGACTTCAACAAGGGGGAGGGAGTG
ATGCCACTGCCTCCGTTTGCAGATCCATTTACCTACCCTGGAGCTTATGAACAGGCTGTA
GGTAACCAGGGGGTATGCAAAGCAAACCTGGCCGTAAACATAAAAGCTTACAAGAACCCA
GAAGAGAAAATAGCCCCTCCTCACAGCAGCATCTACCCCCGGGACGATGTGGACCTGGGG
GTGGAGAACACCCTCATCTGCCATGTCAGCGGGTTCTACCCTGCACCTGTCCGAGTCAGG
TGGACCAGGAACAATCAGAACCTGACAGAGGGAGTACGTCTCAGCACCCCC
>AS5_DAA_s3_176
CACAAAGTTCAGCATATTGATTTAGTTATTAGTGGATGCAGTGATTCAGATGGAGTGGAC
ATGTATGGACTGGATGGGGAAGAGATGTGGTACGCAGACTTCAACAAGGGGGAGGGAGTG
ATGCCACTGCCTCCGTTTGCAGATCCATTTACCTACCCTGGAGCTTATGAACAGGCTGTA
GGTAACCAGGGGGTATGCAAAGCAAACCTGGCCGTAAACATAAAAGCTTACAAGAACCCA
GAAGAGAAAATAGCCCCTCCTCACAGCAGCATCTACCCCCGGGACGATGTGGACCTGGGG
GTGGAGAACACCCTCATCTGCCATGTCAGCGGGTTCTACCCTGCACCTGTCCGAGTCAGG
TGGACCAGGAACAATCAGAACCTGACAGAGGGAGTACGTCTCAGCACCCCC
>AS5_DAA_s4_172
CACAAAGTTCTGCATATTGATTTAGTTATTAGTGGATGCAGTGATTCAGATGGAGTGGAC
ATGTATGGACTGGATGGGGAGGAGATGTGGTACGCAGACTTCAACAAGGGGGAGGGAGTG
ATGCCACTGCCTCCGTTTGCAGATCCATTTACCTACCCTGGAGCTTATGAACAGGCTGTA
GGTAACCAGGGGGTATGCAAAGCAAACCTGGCCGTAAACATAAAAGCTTACAAGAACCCA
GAAGAGAAAATAGCCCCTCCTCACAGCAGCATCTACCCCCGGGACGATGTGGACCTGGGG
GTGGAGAACACCCTCATCTGCCATGTCAGCGGGTTCTACCCTGCACCTGTCCGAGTCAGG
TGGACCAGGAACAATCAGAACCTGACAGAGGGAGTACGTCTCAGCACCCCC
>AS5_DAA_s5_162
CACAAAGTTCTGCATATTGATTTAGTTATTAGTGGATGCAGTGATTCAGATGGAGTGGAC
ATGTATGGACTGGATGGGGAAGAGATGTGGTACGCAGACTTCAACAAGGGGGAGGGAGTG
ATGCCACTGCCTCCGTTTGCAGATCCATTTACCTACCCTGGAGCTTATGAACAGGCTGTA

GGTAACCAGGGGGTATGCAAAGCAAACCTGGCCGTAAACATAAAAGCTTACAAGAACCCA
GAAGGGAAAATAGCCCCTCCTCACAGCAGCATCTACCCCCGGGACGATGTGGACCTGGGG
GTGGAGAACACCCTCATCTGCCATGTCAGCGGGTTCTACCCTGCACCTGTCCGAGTCAGG
TGGACCAGGAACAATCAGAACCTGACAGAGGGAGTACGTCTCAGCACCCCC
>AS6_DAA_s1_21935
CACAAAGTTCTGCATATTGATTTATATATTAGTGGATGCAGTGATTCAGATGGACTGGAC
ATGTATGGACTGGATGGGGAAGAGATGTGGTACGCAGACTTCAACAAGGGGGAGGGAGTG
GTGGCCCTGCCTCCGTTTGCAGATCCATTTACCTTCCCTGGATTTTATGAAGGGGCTGTA
GGTAACCAGGGGGTATGCAAAGCAAACCTGGCCGTAAACATAAAAGCTTACAAGAACCCA
GAAGAGAAAATAGACCCTCCTCACAGCAGCATCTACCCCCGGGACGATGTGGACCTGGGG
GTGGAGAACACCCTCATCTGCCATGTCAGCGGGTTCCACCCTGCACCTGTCCGAGTCAGG
TGGACCAGGAACAATCAGAACCTGACAGAGGGAGTACGTCTCAGCACCCCC
>AS6_DAA_s2_19807
CACAAAGTTCTGCATATTGATTTAGTTATTACTGGATGCAGTGATTCAGATGGAGTGGAC
ATGTATGGACTGGATGGGGAAGAGATGTGGTACGCAGACTTCAACAAGGGGGAGGGAGTG
GTGGCCCTGCCTCCGTTTGCAGATCCATTTACCTTCCATGGAGCTTATGAAGGGGCTGTA
GGTAACCAGGGGGTATGCAAAGCAAACCTGGCCGTAAACATAAAAGCTTACAAGAACCCA
GAAGAGAAAATAGACCCTCCTCACAGCAGCATCTACCCCCGGGACGATGTGGACCTGGGG
GTGGAGAACACCCTCATCTGCCATGTCAGCGGGTTCCACCCTGCACCTGTCCGAGTCAGG
TGGACCAGGAACAATCAGAACCTGACAGAGGGAGTACGTCTCAGCACCCCC
>AS6_DAA_s3_2360
CACAAAGTTCTGCATATTGATTTAGTTATTACTGGATGCAGTGATTCAGATGGAGTGGAC
ATGTATGGACTGGATGGGGAAGAGATGTGGTACGCAGACTTCAACAAGGGGGAGGGAGTG
GTGGCCCTGCCTCCGTTTGCAGATCCATTTACCTTCCCTGGATTTTATGAAGGGGCTGTA
GGTAACCAGGGGGTATGCAAAGCAAACCTGGCCGTAAACATAAAAGCTTACAAGAACCCA
GAAGAGAAAATAGACCCTCCTCACAGCAGCATCTACCCCCGGGACGATGTGGACCTGGGG
GTGGAGAACACCCTCATCTGCCATGTCAGCGGGTTCCACCCTGCACCTGTCCGAGTCAGG
TGGACCAGGAACAATCAGAACCTGACAGAGGGAGTACGTCTCAGCACCCCC
>AS6_DAA_s4_2123
CACAAAGTTCTGCATATTGATTTATATATTAGTGGATGCAGTGATTCAGATGGACTGGAC
ATGTATGGACTGGATGGGGAAGAGATGTGGTACGCAGACTTCAACAAGGGGGAGGGAGTG
GTGGCCCTGCCTCCGTTTGCAGATCCATTTACCTTCCATGGAGCTTATGAAGGGGCTGTA
GGTAACCAGGGGGTATGCAAAGCAAACCTGGCCGTAAACATAAAAGCTTACAAGAACCCA
GAAGAGAAAATAGACCCTCCTCACAGCAGCATCTACCCCCGGGACGATGTGGACCTGGGG
GTGGAGAACACCCTCATCTGCCATGTCAGCGGGTTCCACCCTGCACCTGTCCGAGTCAGG
TGGACCAGGAACAATCAGAACCTGACAGAGGGAGTACGTCTCAGCACCCCC
>AS6_DAA_s5_393
CACAAAGTTCTGCATATTGATTTATATATTAGTGGATGCAGTGATTCAGATGGAGTGGAC
ATGTATGGACTGGATGGGGAAGAGATGTGGTACGCAGACTTCAACAAGGGGGAGGGAGTG
GTGGCCCTGCCTCCGTTTGCAGATCCATTTACCTTCCATGGAGCTTATGAAGGGGCTGTA
GGTAACCAGGGGGTATGCAAAGCAAACCTGGCCGTAAACATAAAAGCTTACAAGAACCCA
GAAGAGAAAATAGACCCTCCTCACAGCAGCATCTACCCCCGGGACGATGTGGACCTGGGG
GTGGAGAACACCCTCATCTGCCATGTCAGCGGGTTCCACCCTGCACCTGTCCGAGTCAGG
TGGACCAGGAACAATCAGAACCTGACAGAGGGAGTACGTCTCAGCACCCCC
>AS7_DAA_s1_31665
CACAAAGTTCTGCATATTGATTTAGTTATTAGTGGATGCAGTGATTCAGATGGAGTGGAC
ATGTATGGACTGGATGGGGAAGAGATGTGGTACGCAGACTTCAACAAGGGGGAGGGAGTG
ATGCCACTGCCTCCGTTTGCAGATCCATTTACCTACCCTGGAGCTTATGAACAGGCTGTA
GGTAACCAGGGGGTATGCAAAGCAAACCTGGCCGTAAACATAAAAGCTTACAAGAACCCA
GAAGAGAAAATAGCCCCTCCTCACAGCAGCATCTACCCCCGGGACGATGTGGACCTGGGG
GTGGAGAACACCCTCATCTGCCATGTCAGCGGGTTCTACCCTGCACCTGTCCGAGTCAGG
TGGACCAGGAACAATCAGAACCTGACAGAGGGAGTACGTCTCAGCACCCCC
>AS7_DAA_s2_29058
CACAAAGTTCTGCATATTGATTTATATATTAGTGGATGCAGTGATTCAGATGGACTGGAC
ATGTATGGACTGGATGGGGAAGAGATGTGGTACGCAGACTTCAACAAGGGGGAGGGAGTG
GTGGCCCTGCCTCCGTTTGCAGATCCATTTACCTTCCCTGGATTTTATGAAGGGGCTGTA
GGTAACCAGGGGGTATGCAAAGCAAACCTGGCCGTAAACATAAAAGCTTACAAGAACCCA
GAAGAGAAAATAGACCCTCCTCACAGCAGCATCTACCCCCGGGACGATGTGGACCTGGGG
GTGGAGAACACCCTCATCTGCCATGTCAGCGGGTTCCACCCTGCACCTGTCCGAGTCAGG
TGGACCAGGAACAATCAGAACCTGACAGAGGGAGTACGTCTCAGCACCCCC
>AS7_DAA_s3_2271

CACAAAGTTCTGCATATTGATTTAGTTATTAGTGGATGCAGTGATTCAGATGGAGTGGAC
ATGTATGGACTGGATGGGGAAGAGATGTGGTACGCAGACTTCAACAAGGGGGAGGGAGTG
ATGCCACTGCCTCCGTTTGCAGATCCATTTACCTACCCTGGAGCTTATGAACAGGCTGTA
GGTAACCAGGGGGTATGCAAAGCAAACCTGGCCGTAAACATAAAAGCTTACAAGAACCCA
GAAGAGAAAATAGACCCTCCTCACAGCAGCATCTACCCCCGGGACGATGTGGACCTGGGG
GTGGAGAACACCCTCATCTGCCATGTCAGCGGGTTCCACCCTGCACCTGTCCGAGTCAGG
TGGACCAGGAACAATCAGAACCTGACAGAGGGAGTACGTCTCAGCACCCCC
>AS7_DAA_s4_2177
CACAAAGTTCTGCATATTGATTTAGTTATTAGTGGATGCAGTGATTCAGATGGAGTGGAC
ATGTATGGACTGGATGGGGAAGAGATGTGGTACGCAGACTTCAACAAGGGGGAGGGAGTG
ATGCCACTGCCTCCGTTTGCAGATCCATTTACCTACCCTGGAGCTTATGAACAGGCTGTA
GGTAACCAGGGGGTATGCAAAGCAAACCTGGCCGTAAACATAAAAGCTTACAAGAACCCA
GAAGAGAAAATAGCCCCTCCTCACAGCAGCATCTACCCCCGGGACGATGTGGACCTGGGG
GTGGAGAACACCCTCATCTGCCATGTCAGCGGGTTCCACCCTGCACCTGTCCGAGTCAGG
TGGACCAGGAACAATCAGAACCTGACAGAGGGAGTACGTCTCAGCACCCCC
>AS7_DAA_s5_2085
CACAAAGTTCTGCATATTGATTTATATATTAGTGGATGCAGTGATTCAGATGGACTGGAC
ATGTATGGACTGGATGGGGAAGAGATGTGGTACGCAGACTTCAACAAGGGGGAGGGAGTG
GTGGCCCTGCCTCCGTTTGCAGATCCATTTACCTTCCCTGGATTTTATGAAGGGGCTGTA
GGTAACCAGGGGGTATGCAAAGCAAACCTGGCCGTAAACATAAAAGCTTACAAGAACCCA
GAAGAGAAAATAGACCCTCCTCACAGCAGCATCTACCCCCGGGACGATGTGGACCTGGGG
GTGGAGAACACCCTCATCTGCCATGTCAGCGGGTTCTACCCTGCACCTGTCCGAGTCAGG
TGGACCAGGAACAATCAGAACCTGACAGAGGGAGTACGTCTCAGCACCCCC
>AS8_DAA_s1_25961
CACAAAGTTCTGCATATTGATTTACATATTATTGGATGCAGTGATTCAGATGGAGTGGAC
ATGTATGGACTGGATGGGGAAGAGATGTGGTACGCAGACTTCAACAAGGGGGAGGGAGTG
GTGGCCCTGCCTCCGTTTGCAGATCCATTTACCTTCCCTGGATTTTATGAAGGGGCTGTA
GGTAACCAGGGGGTATGCAAAGCAAACCTGGCCGTAAACATAAAAGCTTACAAGAACCCA
GAAGAGAAAATAGACCCTCCTCACAGCAGCATCTACCCCCGGGACGATGTGGACCTGGGG
GTGGAGAACACCCTCATCTGCCATGTCAGCGGGTTCTTCCCTGCACCTGTCCGAGTCAGG
TGGACCAGGAACAATCAGAACCTGACAGAGGGAGTACGTCTCAGCACCCCC
>AS8_DAA_s2_22389
CACAAAGTTCTGCATATTGATTTAGTTATTACTGGATGCAGTGATTCAGATGGACTGGAC
ATGTATGGACTGGATGGGGAAGAGATGTGGTACGCAGACTTCAACAAGCAGGAGGGAGTG
GTGGCCCTGCCTCCGTTTGCAGATCCATTTACCTTCCCTGGATTTTATGAACAGGCTGTA
GGTAACCAGGGGGTATGCAAAGGAAACCTGGCCAAATGTATAAAAGCTTACAAGAACCCA
GAAGAGAAAATAGACCCTCCTCACAGCAGCATCTACCCCCGGGACGATGTGGACCTGGGG
GTGGAGAACACCCTCATCTGCCATGTCAGCGGGTTCTTCCCTGCACCTGTCCGAGTCAGG
TGGACCAGGAACAATCAGAACCTGACAGAGGGAGTACGTCTCAGCACCCCC
>AS8_DAA_s3_1672
CACAAAGTTCTGCATATTGATTTACATATTATTGGATGCAGTGATTCAGATGGAGTGGAC
ATGTATGGACTGGATGGGGAAGAGATGTGGTACGCAGACTTCAACAAGGGGGAGGGAGTG
GTGGCCCTGCCTCCGTTTGCAGATCCATTTACCTTCCCTGGATTTTATGAACAGGCTGTA
GGTAACCAGGGGGTATGCAAAGGAAACCTGGCCAAATGTATAAAAGCTTACAAGAACCCA
GAAGAGAAAATAGACCCTCCTCACAGCAGCATCTACCCCCGGGACGATGTGGACCTGGGG
GTGGAGAACACCCTCATCTGCCATGTCAGCGGGTTCTTCCCTGCACCTGTCCGAGTCAGG
TGGACCAGGAACAATCAGAACCTGACAGAGGGAGTACGTCTCAGCACCCCC
>AS8_DAA_s4_1497
CACAAAGTTCTGCATATTGATTTAGTTATTACTGGATGCAGTGATTCAGATGGACTGGAC
ATGTATGGACTGGATGGGGAAGAGATGTGGTACGCAGACTTCAACAAGCAGGAGGGAGTG
GTGGCCCTGCCTCCGTTTGCAGATCCATTTACCTTCCCTGGATTTTATGAAGGGGCTGTA
GGTAACCAGGGGGTATGCAAAGCAAACCTGGCCGTAAACATAAAAGCTTACAAGAACCCA
GAAGAGAAAATAGACCCTCCTCACAGCAGCATCTACCCCCGGGACGATGTGGACCTGGGG
GTGGAGAACACCCTCATCTGCCATGTCAGCGGGTTCTTCCCTGCACCTGTCCGAGTCAGG
TGGACCAGGAACAATCAGAACCTGACAGAGGGAGTACGTCTCAGCACCCCC
>AS8_DAA_s5_876
CACAAAGTTCTGCATATTGATTTAGTTATTACTGGATGCAGTGATTCAGATGGACTGGAC
ATGTATGGACTGGATGGGGAAGAGATGTGGTACGCAGACTTCAACAAGGGGGAGGGAGTG
GTGGCCCTGCCTCCGTTTGCAGATCCATTTACCTTCCCTGGATTTTATGAAGGGGCTGTA
GGTAACCAGGGGGTATGCAAAGCAAACCTGGCCGTAAACATAAAAGCTTACAAGAACCCA
GAAGAGAAAATAGACCCTCCTCACAGCAGCATCTACCCCCGGGACGATGTGGACCTGGGG

```
GTGGAGAACACCCTCATCTGCCATGTCAGCGGGTTCTTCCCTGCACCTGTCCGAGTCAGG
TGGACCAGGAACAATCAGAACCTGACAGAGGGAGTACGTCTCAGCACCCCC
>AS9_DAA_s1_27650
CACAAAGTTCTGCATATTGATTTAGCTATTACTGGATGCAGTGATTCAGATGGACTGGAC
ATGTATGGACTGGATGGGGAAGAGATGTGGTACGCAGACTTCAACAAGGGGGAGGGAGTG
ATGCCACTGCCTCCGTTTGCAGATCCATTTACCTACCCTGGAGCTTATGAAGGGGCTGTA
GGTAACCAGGGGATATGCAAAGCAAACCTGGCCACATGTATAAAAGCTTACAAGAACCCA
GAAGAGAAAATAGCCCCTCCTCACAGCAGCATCTACCCCCGGGACGATGTGGACCTGGGG
GTGGAGAACACCCTCATCTGCCATGTCAGCGGGTTCCACCCTGCACCTGTCCGAGTCAGG
TGGACCAGGAACAATCAGAACCTGACAGAGGGAGTACGTCTCAGCACCCCC
>AS9_DAA_s2_22549
CACAAAGTTCTGCATATTGATTTAGTTATTACTGGATGCAGTGATTCAGATGGAGTGGAC
ATGTATGGACTGGATGGGGAAGAGATGTGGTACGCAGACTTCAACAAGGGGGAGGGAGTG
GTGGCCCTGCCTCCGTTTGCAGATCCATTTACCTTCCATGGAGCTTATGAAGGGGCTGTA
GGTAACCAGGGGGTATGCAAAGCAAACCTGGCCGTAAACATAAAAGCTTACAAGAACCCA
GAAGAGAAAATAGACCCTCCTCACAGCAGCATCTACCCCCGGGACGATGTGGACCTGGGG
GTGGAGAACACCCTCATCTGCCATGTCAGCGGGTTCCACCCTGCACCTGTCCGAGTCAGG
TGGACCAGGAACAATCAGAACCTGACAGAGGGAGTACGTCTCAGCACCCCC
>AS9_DAA_s3_2292
CACAAAGTTCTGCATATTGATTTAGCTATTACTGGATGCAGTGATTCAGATGGACTGGAC
ATGTATGGACTGGATGGGGAAGAGATGTGGTACGCAGACTTCAACAAGGGGGAGGGAGTG
GTGGCCCTGCCTCCGTTTGCAGATCCATTTACCTTCCATGGAGCTTATGAAGGGGCTGTA
GGTAACCAGGGGGTATGCAAAGCAAACCTGGCCGTAAACATAAAAGCTTACAAGAACCCA
GAAGAGAAAATAGACCCTCCTCACAGCAGCATCTACCCCCGGGACGATGTGGACCTGGGG
GTGGAGAACACCCTCATCTGCCATGTCAGCGGGTTCCACCCTGCACCTGTCCGAGTCAGG
TGGACCAGGAACAATCAGAACCTGACAGAGGGAGTACGTCTCAGCACCCCC
>AS9_DAA_s4_2277
CACAAAGTTCTGCATATTGATTTAGTTATTACTGGATGCAGTGATTCAGATGGAGTGGAC
ATGTATGGACTGGATGGGGAAGAGATGTGGTACGCAGACTTCAACAAGGGGGAGGGAGTG
ATGCCACTGCCTCCGTTTGCAGATCCATTTACCTACCCTGGAGCTTATGAAGGGGCTGTA
GGTAACCAGGGGATATGCAAAGCAAACCTGGCCACATGTATAAAAGCTTACAAGAACCCA
GAAGAGAAAATAGCCCCTCCTCACAGCAGCATCTACCCCCGGGACGATGTGGACCTGGGG
GTGGAGAACACCCTCATCTGCCATGTCAGCGGGTTCCACCCTGCACCTGTCCGAGTCAGG
TGGACCAGGAACAATCAGAACCTGACAGAGGGAGTACGTCTCAGCACCCCC
>AS9_DAA_s5_1054
CACAAAGTTCTGCATATTGATTTAGCTATTACTGGATGCAGTGATTCAGATGGACTGGAC
ATGTATGGACTGGATGGGGAAGAGATGTGGTACGCAGACTTCAACAAGGGGGAGGGAGTG
ATGCCACTGCCTCCGTTTGCAGATCCATTTACCTACCCTGGAGCTTATGAAGGGGCTGTA
GGTAACCAGGGGATATGCAAAGCAAACCTGGCCACATGTATAAAAGCTTACAAGAACCCA
GAAGAGAAAATAGACCCTCCTCACAGCAGCATCTACCCCCGGGACGATGTGGACCTGGGG
GTGGAGAACACCCTCATCTGCCATGTCAGCGGGTTCCACCCTGCACCTGTCCGAGTCAGG
TGGACCAGGAACAATCAGAACCTGACAGAGGGAGTACGTCTCAGCACCCCC
>AS10_DAA_s1_90816
CACAAAGTTCTGCATATTGATTTACATATTATTGGATGCAGTGATTCAGATGGAGTGGAC
ATGTATGGACTGGATGGGGAAGAGATGTGGTACGCAGACTTCAACAAGGGGGAGGGAGTG
GTGGCCCTGCCTCCGTTTGCAGATCCATTTACCTTCCCTGGATTTTATGAAGGGGCTGTA
GGTAACCAGGGGGTATGCAAAGCAAACCTGGCCGTAAACATAAAAGCTTACAAGAACCCA
GAAGAGAAAATAGACCCTCCTCACAGCAGCATCTACCCCCGGGACGATGTGGACCTGGGG
GTGGAGAACACCCTCATCTGCCATGTCAGCGGGTTCTTCCCTGCACCTGTCCGAGTCAGG
TGGACCAGGAACAATCAGAACCTGACAGAGGGAGTACGTCTCAGCACCCCC
>AS10_DAA_s2_422
CACAAAGTTCCGCATATTGATTTACATATTATTGGATGCAGTGATTCAGATGGAGTGGAC
ATGTATGGACTGGATGGGGAAGAGATGTGGTACGCAGACTTCAACAAGGGGGAGGGAGTG
GTGGCCCTGCCTCCGTTTGCAGATCCATTTACCTTCCCTGGATTTTATGAAGGGGCTGTA
GGTAACCAGGGGGTATGCAAAGCAAACCTGGCCGTAAACATAAAAGCTTACAAGAACCCA
GAAGAGAAAATAGACCCTCCTCACAGCAGCATCTACCCCCGGGACGATGTGGACCTGGGG
GTGGAGAACACCCTCATCTGCCATGTCAGCGGGTTCTTCCCTGCACCTGTCCGAGTCAGG
TGGACCAGGAACAATCAGAACCTGACAGAGGGAGTACGTCTCAGCACCCCC
>AS10_DAA_s3_419
CACAAAGTTCTGCATATTGATTTACATATTATTGGATGCAGTGATTCAGATGGAGTGGAC
ATGTATGGACTGGATGGGGAAGAGATGTGGTACGCAGACTTCAACAAGGGGGAGGGAGTG
```

GTGGCCCTGCCTCCGTTTGCAGATCCATTTACCTTCCCTGGATTTTATGAAGGGGCTGTA
GGTAACCAGGGGGTATGCAAAGCAAACCTGGCCGTAAACATAAAAGCTTACAAGAACCCA
GAAGAGAAAATAGACCCTCCTCACAGCAGCATCTACCCCCGGGACGATGTGGACCTGGGG
GTGGAGAACACCCTCATCTGCCATGTCAGCGGGTTCTTCCCTGCACCTGTCCGAGTCAGG
TGGACCAGGAACAATCAGAACCTGACAGAGGGGGTACGTCTCAGCACCCCC
>AS10_DAA_s4_410
CACAAAGTTCTGCATATTGATTTACATATTATTGGATGCAGTGATTCAGATGGAGTGGAC
ATGTATGGACTGGATGGGGAAGAGATGTGGTACGCAGACTTCAACAAGGGGGGGGGAGTG
GTGGCCCTGCCTCCGTTTGCAGATCCATTTACCTTCCCTGGATTTTATGAAGGGGCTGTA
GGTAACCAGGGGGTATGCAAAGCAAACCTGGCCGTAAACATAAAAGCTTACAAGAACCCA
GAAGAGAAAATAGACCCTCCTCACAGCAGCATCTACCCCCGGGACGATGTGGACCTGGGG
GTGGAGAACACCCTCATCTGCCATGTCAGCGGGTTCTTCCCTGCACCTGTCCGAGTCAGG
TGGACCAGGAACAATCAGAACCTGACAGAGGGAGTACGTCTCAGCACCCCC
>AS10_DAA_s5_407
CACAAAGTTCTGCATATTGATTTACATATTATTGGATGCAGTGATTCAGATGGAGTGGAC
ATGTATGGACTGGATGGGGAAGAGATGTGGTACGCAGACTTCAACAAGGGGGAGGGAGTG
GTGGCCCTGCCTCCGTTTGCGGATCCATTTACCTTCCCTGGATTTTATGAAGGGGCTGTA
GGTAACCAGGGGGTATGCAAAGCAAACCTGGCCGTAAACATAAAAGCTTACAAGAACCCA
GAAGAGAAAATAGACCCTCCTCACAGCAGCATCTACCCCCGGGACGATGTGGACCTGGGG
GTGGAGAACACCCTCATCTGCCATGTCAGCGGGTTCTTCCCTGCACCTGTCCGAGTCAGG
TGGACCAGGAACAATCAGAACCTGACAGAGGGAGTACGTCTCAGCACCCCC

MHC class II beta (DAB) nucleotide sequences:

>AS1_DAB2_s1_63791
GTTTTGTCCATATTCTCTGGAACAGATGGATATTTTGAACAGGTTGTGAGACAGTGCCGA
TACTCCTCAAAGGACCTGCAGGGTATAGAGTTTATAGACTCTTATGTTTTCAATAAGGCT
GAATATGTCAGATTCAACAGCACTGTGGGGAAGTATGTTGGATACACTGAGCTGGGAGTG
AAGAATGCAGAAGCCTGGAACAAAGGTCCTGAGCTGGCTGTAGAGCTAGGGGAGCTGGAG
CGTTACTGTAAGCTTAACGCTCCTATCGACTACAGCGCCATACTGGACAAGACAGTTGAG
CCCCATGTCAGACTGAGCTCAGTGGCTCCCCCCAGTGGCAGACACCCTGCCATGCTGATG
TGCAGCGCCTACGACTTCTACCCCAAACCAATCAGAGTGACCTGGCTGAGGGACGGACGT
GAGGTGAAGTCTGATGTGACCTCCACTGAGGAG
>AS1_DAB2_s2_20621
GTTTTGTCCATATTCTCTGGAACAGATGGATATTTTTATCATATGATGACACAGTGCCGA
TACTCCTCAAAGGACCTGCAGGGTATAGAGTTGATTACCTCGTATGTTTTCAATCAGGCT
GAAAATATCAGATTCAACAGCACTGTGGGGAAGTTTGTTGGATACACTGAGCATGGAGTG
AAGAATGCAGAAGCCTGGAACAAAGGTCCTGAGCTGGCTGGAGAGCTAGGGGTGCTGGAG
CGTTACTGTAAGTTTAACGCTCCTATCGACTACAGCGCCATACTGGACAAGACAGTTGAG
CCCCATGTCAGACTGAGCTCAGTGGCTCCCCCCAGTGGCAGACACCCTGCCATGCTGATG
TGCAGCGCCTACGACTTCTTCCCCAAACCAATCAGAGTGACCTGGCTGAGGGACGGACGT
GAGGTGAAGTCTGATGTGACCTCCACTGAGGAG
>AS1_DAB2_s3_5382
GTTTTGTCCATATTCTCTGGAACAGATGGATATTTTTATCATATGATGACACAGTGCCGA
TACTCCTCAAAGGACCTGCAGGGTATAGAGTTGATTACCTCGTATGTTTTCAATCAGGCT
GAAAATATCAGATTCAACAGCACTGTGGGGAAGTTTGTTGGATACACTGAGCATGGAGTG
AAGAATGCAGAAGCCTGGAACAAAGGTCCTGAGCTGGCTGGAGAGCTAGGGGTGCTGGAG
CGTTACTGTAAGTTTAACGCTCCTATCGACTACAGCGCCATACTGGACAAGACAGTTGAG
CCCCATGTCAGACTGAGCTCAGTGGCTCCCCCCAGTGGCAGACACCCTGCCATGCTGATG
TGCAGCGCCTACGACTTCTACCCCAAACCAATCAGAGTGACCTGGCTGAGGGACGGACGT
GAGGTGAAGTCTGATGTGACCTCCACTGAGGAG
>AS1_DAB2_s4_4227
GTTTTGTCCATATTCTCTGGAACAGATGGATATTTTGAACAGGTTGTGAGACAGTGCCGA
TACTCCTCAAAGGACCTGCAGGGTATAGAGTTTATAGACTCTTATGTTTTCAATAAGGCT
GAATATGTCAGATTCAACAGCACTGTGGGGAAGTATGTTGGATACACTGAGCTGGGAGTG
AAGAATGCAGAAGCCTGGAACAAAGGTCCTGAGCTGGCTGTAGAGCTAGGGGAGCTGGAG
CGTTACTGTAAGCTTAACGCTCCTATCGACTACAGCGCCATACTGGACAAGACAGTTGAG
CCCCATGTCAGACTGAGCTCAGTGGCTCCCCCCAGTGGCAGACACCCTGCCATGCTGATG
TGCAGCGCCTACGACTTCTTCCCCAAACCAATCAGAGTGACCTGGCTGAGGGACGGACGT
GAGGTGAAGTCTGATGTGACCTCCACTGAGGAG
>AS1_DAB2_s5_3463

GTTTTGTCCATATTCTCTGGAACAGATGGATATTTTGAACAGGTTGTGAGACAGTGCCGA
TACTCCTCAAAGGACCTGCAGGGTATAGAGTTTATAGACTCTTATGTTTTCAATAAGGCT
GAATATGTCAGATTCAACAGCACTGTGGGGAAGTATGTTGGATACACTGAGCTGGGAGTG
AAGAATGCAGAAGCCTGGAACAAAGGTCCTGAGCTGGCTGTAGAGCTAGGGGAGCTGGAG
CGTTTCTGTAAGCATAACGCTGCTATCTACTACAGTGCCATACTGGACAAGACAGTTGAG
CCCCATGTCAGACTGAGCTCAGTGGCTCCCCCCAGTGGCAGACACCCTGCCATGCTGATG
TGCAGCGCCTACGACTTCTACCCCAAACCAATCAGAGTGACCTGGCTGAGGGACGGACGT
GAGGTGAAGTCTGATGTGACCTCCACTGAGGAG
>AS2_DAB1_s1_30852
GTTTCCCTGACCCTGGTTTTGTCCATATTCTCTGGAACAGATGGATATTTTTTTCAGGTT
GTGAGACAGTGCCGATACTCCTCAAAGGACCTGCAGGGTATAGAGTTTATACACTCTTAT
GTTTTCAATCAGGCTGAAAATATCAGATTCAACAGCACTGTGGGGAAGTATGTTGGATAC
ACTGAGCTGGGAGTGAAGAATGCAGAAGCCTGGAACAAAGGTCCTGAGCTGGCTGTAGAG
CTAGGGGAGCTGGAGCGTTACTGTAAGCATAACGCTGATATCGACTACAGCGCCATACTG
GACAAGACAGTTGAGCCCCATGTCAGACTGAGCTCAGTGGCTCCCCCCAGTGGCAGACAC
CCTGCCATGCTGATGTGCAGCGCCTACGACTTCTACCCCAAACCAATCAGAGTGACCTGG
CTGAGGGACGGACGTGAGGTGAAGTCTGATGTGACCTCCACTGAGGAG
>AS2_DAB1_s2_17876
GTTTCCCTGACCCTGGTTTTGTCCATATTCTCTGGAACAGATGGATATTTTTATCATATG
ATGACACAGTGCCGATACTCCTCAAAGGACCTGCAGGGTATAGAGTTGATTACCTCGTAT
GTTTTCAATCAGGCTGAAAATATCAGATTCAACAGCACTGTGGGGAAGTTTGTTGGATAC
ACTGAGCATGGAGTGAAGAATGCAGAAGCCTGGAACAAAGGTCCTGAGCTGGCTGGAGAG
CTAGGGGTGCTGGAGCGTTACTGTAAGTTTAACGCTCCTATCGACTACAGCGCCATACTG
GACAAGACAGTTGAGCCCCATGTCAGACTGAGCTCAGTGGCTCCCCCCAGTGGCAGACAC
CCTGCCATGCTGATGTGCAGCGCCTACGACTTCTTCCCCAAACCAATCAGAGTGACCTGG
CTGAGGGACGGACGTGAGGTGAAGTCTGATGTGACCTCCACTGAGGAG
>AS2_DAB1_s3_3147
GTTTCCCTGACCCTGGTTTTGTCCATATTCTCTGGAACAGATGGATATTTTTATCATATG
ATGACACAGTGCCGATACTCCTCAAAGGACCTGCAGGGTATAGAGTTGATTACCTCGTAT
GTTTTCAATCAGGCTGAAAATATCAGATTCAACAGCACTGTGGGGAAGTTTGTTGGATAC
ACTGAGCATGGAGTGAAGAATGCAGAAGCCTGGAACAAAGGTCCTGAGCTGGCTGGAGAG
CTAGGGGTGCTGGAGCGTTACTGTAAGTTTAACGCTCCTATCGACTACAGCGCCATACTG
GACAAGACAGTTGAGCCCCATGTCAGACTGAGCTCAGTGGCTCCCCCCAGTGGCAGACAC
CCTGCCATGCTGATGTGCAGCGCCTACGACTTCTACCCCAAACCAATCAGAGTGACCTGG
CTGAGGGACGGACGTGAGGTGAAGTCTGATGTGACCTCCACTGAGGAG
>AS2_DAB1_s4_2822
GTTTCCCTGACCCTGGTTTTGTCCATATTCTCTGGAACAGATGGATATTTTTTTCAGGTT
GTGAGACAGTGCCGATACTCCTCAAAGGACCTGCAGGGTATAGAGTTTATACACTCTTAT
GTTTTCAATCAGGCTGAAAATATCAGATTCAACAGCACTGTGGGGAAGTATGTTGGATAC
ACTGAGCTGGGAGTGAAGAATGCAGAAGCCTGGAACAAAGGTCCTGAGCTGGCTGTAGAG
CTAGGGGAGCTGGAGCGTTACTGTAAGCATAACGCTGATATCGACTACAGCGCCATACTG
GACAAGACAGTTGAGCCCCATGTCAGACTGAGCTCAGTGGCTCCCCCCAGTGGCAGACAC
CCTGCCATGCTGATGTGCAGCGCCTACGACTTCTTCCCCAAACCAATCAGAGTGACCTGG
CTGAGGGACGGACGTGAGGTGAAGTCTGATGTGACCTCCACTGAGGAG
>AS2_DAB1_s5_1143
GTTTCCCTGACCCTGGTTTTGTCCATATTCTCTGGAACAGATGGATATTTTTATCATATG
ATGACACAGTGCCGATACTCCTCAAAGGACCTGCAGGGTATAGAGTTGATTACCTCGTAT
GTTTTCAATCAGGCTGAAAATATCAGATTCAACAGCACTGTGGGGAAGTTTGTTGGATAC
ACTGAGCATGGAGTGAAGAATGCAGAAGCCTGGAACAAAGGTCCTGAGCTGGCTGTAGAG
CTAGGGGAGCTGGAGCGTTACTGTAAGCATAACGCTGATATCGACTACAGCGCCATACTG
GACAAGACAGTTGAGCCCCATGTCAGACTGAGCTCAGTGGCTCCCCCCAGTGGCAGACAC
CCTGCCATGCTGATGTGCAGCGCCTACGACTTCTACCCCAAACCAATCAGAGTGACCTGG
CTGAGGGACGGACGTGAGGTGAAGTCTGATGTGACCTCCACTGAGGAG
>AS3_DAB1_s1_10179
GTTTCCCTGACCCTGGTTTTGTCCATATTCTCTGGAACAGATGGATATTTTGAACAGGTT
GTGAGACAGTGCCGATACTCCTCAAAGGACCTGCAGGGTATAGAGTTTATAGACTCTTAT
GTTTTCAATAAGGCTGAATATGTCAGATTCAACAGCACTGTGGGGAAGTATGTTGGATAC
ACTGAGCTGGGAGTGAAGAATGCAGAAGCCTGGAACAAAGGTCCTGAGCTGGCTGTAGAG
CTAGGGGAGCTGGAGCGTTTCTGTAAGCATAACGCTGATCTCCACTACCGCGCCATACTG
GACAAGACAGTTGAGCCCCATGTCAGACTGAGCTCAGTGGCTCCCCCCAGTGGCAGACAC
CCTGCCATGCTGATGTGCAGCGCCTACGACTTCTACCCCAAACCAATCAGAGTGACCTGG

CTGAGGGATGGACGTGAGGTGAAGTCTGATGTGACCTCCACTGAGGAG
>AS3_DAB1_s2_7127
GTTTCCCTGACCCTGGTTTTGTCCATATTCTCTGGAACAGATGGATATTTTTATCAGAGG
GTGTCAGAGTGCCGATACTCCTCAAAGGACCTGCAGGGTATAGAGTTTATAGACTCTTAT
GTTTTCAATAAGGCTGAATATGTCAGATTCAACAGCACTGTGGGGAAGTATGTTGGATAC
ACTGAGTATGGAGTGAAGAATGCAGAAGCCTGGAACAAAGGTCCTGAGCTGGCTGGAGAG
CTAGGGGAGCTGGAGCGTGTCTGTAAGCATAACGCTCCTATCGACTACAGCGCCATACTG
GACAAGACAGTTGAGCCCCATGTCAGACTGAGCTCAGTGGCTCCCCCCAGTGGCAGACAC
CCTGCCATGCTGATGTGCAGCGCCTACGACTTCTTCCCCAAACCAATCAGAGTGACCTGG
CTGAGGGACGGACGTGAGGTGAAGTCTGATGTGACCTCCACTGAGGAG
>AS3_DAB1_s3_1585
GTTTCCCTGACCCTGGTTTTGTCCATATTCTCTGGAACAGATGGATATTTTTATCAGAGG
GTGTCAGAGTGCCGATACTCCTCAAAGGACCTGCAGGGTATAGAGTTTATAGACTCTTAT
GTTTTCAATAAGGCTGAATATGTCAGATTCAACAGCACTGTGGGGAAGTATGTTGGATAC
ACTGAGTATGGAGTGAAGAATGCAGAAGCCTGGAACAAAGGTCCTGAGCTGGCTGGAGAG
CTAGGGGAGCTGGAGCGTGTCTGTAAGCATAACGCTCCTATCGACTACAGCGCCATACTG
GACAAGACAGTTGAGCCCCATGTCAGACTGAGCTCAGTGGCTCCCCCCAGTGGCAGACAC
CCTGCCATGCTGATGTGCAGCGCCTACGACTTCTACCCCAAACCAATCAGAGTGACCTGG
CTGAGGGATGGACGTGAGGTGAAGTCTGATGTGACCTCCACTGAGGAG
>AS3_DAB1_s4_1553
GTTTCCCTGACCCTGGTTTTGTCCATATTCTCTGGAACAGATGGATATTTTGAACAGGTT
GTGAGACAGTGCCGATACTCCTCAAAGGACCTGCAGGGTATAGAGTTTATAGACTCTTAT
GTTTTCAATAAGGCTGAATATGTCAGATTCAACAGCACTGTGGGGAAGTATGTTGGATAC
ACTGAGTATGGAGTGAAGAATGCAGAAGCCTGGAACAAAGGTCCTGAGCTGGCTGGAGAG
CTAGGGGAGCTGGAGCGTGTCTGTAAGCATAACGCTCCTATCGACTACAGCGCCATACTG
GACAAGACAGTTGAGCCCCATGTCAGACTGAGCTCAGTGGCTCCCCCCAGTGGCAGACAC
CCTGCCATGCTGATGTGCAGCGCCTACGACTTCTTCCCCAAACCAATCAGAGTGACCTGG
CTGAGGGACGGACGTGAGGTGAAGTCTGATGTGACCTCCACTGAGGAG
>AS3_DAB1_s5_1509
GTTTCCCTGACCCTGGTTTTGTCCATATTCTCTGGAACAGATGGATATTTTTATCAGAGG
GTGTCAGAGTGCCGATACTCCTCAAAGGACCTGCAGGGTATAGAGTTTATAGACTCTTAT
GTTTTCAATAAGGCTGAATATGTCAGATTCAACAGCACTGTGGGGAAGTATGTTGGATAC
ACTGAGCTGGGAGTGAAGAATGCAGAAGCCTGGAACAAAGGTCCTGAGCTGGCTGTAGAG
CTAGGGGAGCTGGAGCGTTTCTGTAAGCATAACGCTGATCTCCACTACCGCGCCATACTG
GACAAGACAGTTGAGCCCCATGTCAGACTGAGCTCAGTGGCTCCCCCCAGTGGCAGACAC
CCTGCCATGCTGATGTGCAGCGCCTACGACTTCTACCCCAAACCAATCAGAGTGACCTGG
CTGAGGGATGGACGTGAGGTGAAGTCTGATGTGACCTCCACTGAGGAG
>AS5_DAB2_s1_11545
GTTTTGTCCATATTCTCTGGAACAGATGGATATTTTTATCATATGATGACACAGTGCCGA
TACTCCTCAAAGGACCTGCAGGGTATAGAGTTGATTACCTCGTATGTTTTCAATCAGGCT
GAAAATATCAGATTCAACAGCACTGTGGGGAAGTTTGTTGGATACACTGAGCATGGAGTG
AAGAATGCAGAAGCCTGGAACAAAGGTCCTGAGCTGGCTGGAGAGCTAGGGGTGCTGGAG
CGTTACTGTAAGTTTAACGCTCCTATCGACTACAGCGCCATACTGGACAAGACAGTTGAG
CCCCATGTCAGACTGAGCTCAGTGGCTCCCCCCAGTGGCAGACACCCTGCCATGCTGATG
TGCAGCGCCTACGACTTCTTCCCCAAACCAATCAGAGTGACCTGGCTGAGGGACGGACGT
GAGGTGAAGTCTGATGTGACCTCCACTGAGGAG
>AS5_DAB2_s2_9933
GTTTTGTCCATATTCTCTGGAACAGATGGATATTTTTATCATATGATGAGACAGTGCCGA
TACTCCTCAAAGGACCTGCAGGGTATAGAGTTGATTACCTCGTATGTTTTCAATCAGGCT
GAAAATATCAGATTCAACAGCACTGTGGGGAAGTTTGTTGGATACACTGAGCATGGAGTG
AAGAATGCAGAAGCCTGGAACAAAGGTCCTGAGCTGGCTGGAGAGCTAGGGGAGCTGGAG
CGTGTCTGTAAGCATAACGCTCCTATCTACTACAGCGCCATACTGGACAAGACAGTTGAG
CCCCATGTCAGACTGAGCTCAGTGGCTCCCCCCAGTGGCAGACACCCTGCCATGCTGATG
TGCAGCGCCTACGACTTCTTCCCCAAACCAATCAGAGTGACCTGGCTGAGGGACGGACGT
GAGGTGAAGTCTGATGTGACCTCCACTGAGGAG
>AS5_DAB2_s3_1500
GTTTTGTCCATATTCTCTGGAACAGATGGATATTTTTATCATATGATGAGACAGTGCCGA
TACTCCTCAAAGGACCTGCAGGGTATAGAGTTGATTACCTCGTATGTTTTCAATCAGGCT
GAAAATATCAGATTCAACAGCACTGTGGGGAAGTTTGTTGGATACACTGAGCATGGAGTG
AAGAATGCAGAAGCCTGGAACAAAGGTCCTGAGCTGGCTGGAGAGCTAGGGGTGCTGGAG
CGTTACTGTAAGTTTAACGCTCCTATCGACTACAGCGCCATACTGGACAAGACAGTTGAG

CCCCATGTCAGACTGAGCTCAGTGGCTCCCCCCAGTGGCAGACACCCTGCCATGCTGATG
TGCAGCGCCTACGACTTCTTCCCCAAACCAATCAGAGTGACCTGGCTGAGGGACGGACGT
GAGGTGAAGTCTGATGTGACCTCCACTGAGGAG
>AS5_DAB2_s4_1433
GTTTTGTCCATATTCTCTGGAACAGATGGATATTTTTATCATATGATGACACAGTGCCGA
TACTCCTCAAAGGACCTGCAGGGTATAGAGTTGATTACCTCGTATGTTTTCAATCAGGCT
GAAAATATCAGATTCAACAGCACTGTGGGGAAGTTTGTTGGATACACTGAGCATGGAGTG
AAGAATGCAGAAGCCTGGAACAAAGGTCCTGAGCTGGCTGGAGAGCTAGGGGAGCTGGAG
CGTGTCTGTAAGCATAACGCTCCTATCTACTACAGCGCCATACTGGACAAGACAGTTGAG
CCCCATGTCAGACTGAGCTCAGTGGCTCCCCCCAGTGGCAGACACCCTGCCATGCTGATG
TGCAGCGCCTACGACTTCTTCCCCAAACCAATCAGAGTGACCTGGCTGAGGGACGGACGT
GAGGTGAAGTCTGATGTGACCTCCACTGAGGAG
>AS5_DAB2_s5_994
GTTTTGTCCATATTCTCTGGAACAGATGGATATTTTGAACAGGTTGTGAGACAGTGCCGA
TACTCCTCAAAGGACCTGCAGGGTATAGAGTTTATAGACTCTTATGTTTTCAATAAGGCT
GAATATGTCAGATTCAACAGCACTGTGGGGAAGTATGTTGGATACACTGAGCTGGGAGTG
AAGAATGCAGAAGCCTGGAACAAAGGTCCTGAGCTGGCTGTAGAGCTAGGGGAGCTGGAG
CGTTTCTGTAAGCATAACGCTGCTATCTACTACAGTGCCATACTGGACAAGACAGTTGAG
CCCCATGTCAGACTGAGCTCAGTGGCTCCCCCCAGTGGCAGACACCCTGCCATGCTGATG
TGCAGCGCCTACGACTTCTACCCCAAACCAATCAGAGTGACCTGGCTGAGGGACGGACGT
GAGGTGAAGTCTGATGTGACCTCCACTGAGGAG
>AS6_DAB1_s1_10396
GTTTCCCTGACCCTGGTTTTGTCCATATTCTCTGGAACAGATGGATATTTTGAACAGGTT
GTGAGACAGTGCCGATACTCCTCAAAGGACCTGCAGGGTATAGAGTTTATAGACTCTTAT
GTTTTCAATAAGGCTGAATATGTCAGATTCAACAGCACTGTGGGGAAGTATGTTGGATAC
ACTGAGCTGGGAGTGAAGAATGCAGAAGCCTGGAACAAAGGTCCTGAGCTGGCTGTAGAG
CTAGGGGAGCTGGAGCGTTACTGTAAGCTTAACGCTCCTATCGACTACAGCGCCATACTG
GACAAGACAGTTGAGCCCCATGTCAGACTGAGCTCAGTGGCTCCCCCCAGTGGCAGACAC
CCTGCCATGCTGATGTGCAGCGCCTACGACTTCTACCCCAAACCAATCAGAGTGACCTGG
CTGAGGGATGGACGTGAGGTGAAGTCTGATGTGACCTCCACTGAGGAG
>AS6_DAB1_s2_9420
GTTTCCCTGACCCTGGTTTTGTCCATATTCTCTGGAACAGATGGATATTTTGAACAGGTT
GTGAGACAGTGCCGATACTCCTCAAAGGACCTGCAGGGTATAGAGTTTATAGACTCTTAT
GTTTTCAATAAGGCTGAATATGTCAGATTCAACAGCACTGTGGGGAAGTATGTTGGATAC
ACTGAGCTGGGAGTGAAGAATGCAGAAGCCTGGAACAAAGGTCCTGAGCTGGCTGTAGAG
CTAGGGGAGCTGGAGCGTTTCTGTAAGCATAACGCTGCTATCTACTACAGTGCCATACTG
GACAAGACAGTTGAGCCCCATGTCAGACTGAGCTCAGTGGCTCCCCCCAGTGGCAGACAC
CCTGCCATGCTGATGTGCAGCGCCTACGACTTCTACCCCAAACCAATCAGAGTGACCTGG
CTGAGGGACGGACGTGAGGTGAAGTCTGATGTGACCTCCACTGAGGAG
>AS6_DAB1_s3_1464
GTTTCCCTGACCCTGGTTTTGTCCATATTCTCTGGAACAGATGGATATTTTGAACAGGTT
GTGAGACAGTGCCGATACTCCTCAAAGGACCTGCAGGGTATAGAGTTTATAGACTCTTAT
GTTTTCAATAAGGCTGAATATGTCAGATTCAACAGCACTGTGGGGAAGTATGTTGGATAC
ACTGAGCTGGGAGTGAAGAATGCAGAAGCCTGGAACAAAGGTCCTGAGCTGGCTGTAGAG
CTAGGGGAGCTGGAGCGTTACTGTAAGCTTAACGCTCCTATCGACTACAGCGCCATACTG
GACAAGACAGTTGAGCCCCATGTCAGACTGAGCTCAGTGGCTCCCCCCAGTGGCAGACAC
CCTGCCATGCTGATGTGCAGCGCCTACGACTTCTACCCCAAACCAATCAGAGTGACCTGG
CTGAGGGACGGACGTGAGGTGAAGTCTGATGTGACCTCCACTGAGGAG
>AS6_DAB1_s4_1282
GTTTCCCTGACCCTGGTTTTGTCCATATTCTCTGGAACAGATGGATATTTTGAACAGGTT
GTGAGACAGTGCCGATACTCCTCAAAGGACCTGCAGGGTATAGAGTTTATAGACTCTTAT
GTTTTCAATAAGGCTGAATATGTCAGATTCAACAGCACTGTGGGGAAGTATGTTGGATAC
ACTGAGCTGGGAGTGAAGAATGCAGAAGCCTGGAACAAAGGTCCTGAGCTGGCTGTAGAG
CTAGGGGAGCTGGAGCGTTTCTGTAAGCATAACGCTGCTATCTACTACAGTGCCATACTG
GACAAGACAGTTGAGCCCCATGTCAGACTGAGCTCAGTGGCTCCCCCCAGTGGCAGACAC
CCTGCCATGCTGATGTGCAGCGCCTACGACTTCTACCCCAAACCAATCAGAGTGACCTGG
CTGAGGGATGGACGTGAGGTGAAGTCTGATGTGACCTCCACTGAGGAG
>AS6_DAB1_s5_82
GTTTCCCTGACCCTGGTTTTGTCCATATTCTCTGGAACAGATGGATATTTTGAACAGGTT
GTGAGACAGTGCCGATACTCCTCAAAGGACCTGCAGGGTATAGAGTTTATAGACTCTTAT
GTTTTCAATAAGGCTGAATATGTCAGATTCAACAGCACTGTGGGGAAGTATGTTGGATAC

ACTGAGCTGGGAGTGAAGAATGCAGAAGCCTGGAACAAAGGTCCTGAGCTGGCTGTAGAG
CTAGGGGAGCTGGAGCGTTTCTGTAAGCATAACGCTGCTATCTACTACAGGGCCATACTG
GACAAGACAGTTGAGCCCCATGTCAGACTGAGCTCAGTGGCTCCCCCCAGTGGCAGACAC
CCTGCCATGCTGATGTGCAGCGCCTACGACTTCTACCCCAAACCAATCAGAGTGACCTGG
CTGAGGGACGGACGTGAGGTGAAGTCTGATGTGACCTCCACTGAGGAG
>AS7_DAB2_s1_71872
GTTTTGTCCATATTCTCTGGAACAGATGGATATTTTGAACAGGTTGTGAGACAGTGCCGA
TACTCCTCAAAGGACCTGCAGGGTATAGAGTTTATAGACTCTTATGTTTTCAATAAGGCT
GAATATGTCAGATTCAACAGCACTGTGGGGAAGTATGTTGGATACACTGAGCTGGGAGTG
AAGAATGCAGAAGCCTGGAACAAAGGTCCTGAGCTGGCTGTAGAGCTAGGGGAGCTGGAG
CGTTTCTGTAAGCATAACGCTGCTATCTACTACAGTGCCATACTGGACAAGACAGTTGAG
CCCCATGTCAGACTGAGCTCAGTGGCTCCCCCCAGTGGCAGACACCCTGCCATGCTGATG
TGCAGCGCCTACGACTTCTACCCCAAACCAATCAGAGTGACCTGGCTGAGGGACGGACGT
GAGGTGAAGTCTGATGTGACCTCCACTGAGGAG
>AS7_DAB2_s2_24758
GTTTTGTCCATATTCTCTGGAACAGATGGATATTTTTATCATATGATGACACAGTGCCGA
TACTCCTCAAAGGACCTGCAGGGTATAGAGTTGATTACCTCGTATGTTTTCAATCAGGCT
GAAAATATCAGATTCAACAGCACTGTGGGGAAGTTTGTTGGATACACTGAGCATGGAGTG
AAGAATGCAGAAGCCTGGAACAAAGGTCCTGAGCTGGCTGGAGAGCTAGGGGTGCTGGAG
CGTTACTGTAAGTTTAACGCTCCTATCGACTACAGCGCCATACTGGACAAGACAGTTGAG
CCCCATGTCAGACTGAGCTCAGTGGCTCCCCCCAGTGGCAGACACCCTGCCATGCTGATG
TGCAGCGCCTACGACTTCTTCCCCAAACCAATCAGAGTGACCTGGCTGAGGGACGGACGT
GAGGTGAAGTCTGATGTGACCTCCACTGAGGAG
>AS7_DAB2_s3_7352
GTTTTGTCCATATTCTCTGGAACAGATGGATATTTTTATCATATGATGACACAGTGCCGA
TACTCCTCAAAGGACCTGCAGGGTATAGAGTTGATTACCTCGTATGTTTTCAATCAGGCT
GAAAATATCAGATTCAACAGCACTGTGGGGAAGTTTGTTGGATACACTGAGCATGGAGTG
AAGAATGCAGAAGCCTGGAACAAAGGTCCTGAGCTGGCTGGAGAGCTAGGGGTGCTGGAG
CGTTACTGTAAGTTTAACGCTCCTATCGACTACAGCGCCATACTGGACAAGACAGTTGAG
CCCCATGTCAGACTGAGCTCAGTGGCTCCCCCCAGTGGCAGACACCCTGCCATGCTGATG
TGCAGCGCCTACGACTTCTACCCCAAACCAATCAGAGTGACCTGGCTGAGGGACGGACGT
GAGGTGAAGTCTGATGTGACCTCCACTGAGGAG
>AS7_DAB2_s4_5908
GTTTTGTCCATATTCTCTGGAACAGATGGATATTTTGAACAGGTTGTGAGACAGTGCCGA
TACTCCTCAAAGGACCTGCAGGGTATAGAGTTTATAGACTCTTATGTTTTCAATAAGGCT
GAATATGTCAGATTCAACAGCACTGTGGGGAAGTATGTTGGATACACTGAGCTGGGAGTG
AAGAATGCAGAAGCCTGGAACAAAGGTCCTGAGCTGGCTGTAGAGCTAGGGGAGCTGGAG
CGTTTCTGTAAGCATAACGCTGCTATCTACTACAGTGCCATACTGGACAAGACAGTTGAG
CCCCATGTCAGACTGAGCTCAGTGGCTCCCCCCAGTGGCAGACACCCTGCCATGCTGATG
TGCAGCGCCTACGACTTCTTCCCCAAACCAATCAGAGTGACCTGGCTGAGGGACGGACGT
GAGGTGAAGTCTGATGTGACCTCCACTGAGGAG
>AS7_DAB2_s5_1869
GTTTTGTCCATATTCTCTGGAACAGATGGATATTTTGAACAGGTTGTGAGACAGTGCCGA
TACTCCTCAAAGGACCTGCAGGGTATAGAGTTTATAGACTCTTATGTTTTCAATAAGGCT
GAATATGTCAGATTCAACAGCACTGTGGGGAAGTATGTTGGATACACTGAGCTGGGAGTG
AAGAATGCAGAAGCCTGGAACAAAGGTCCTGAGCTGGCTGGAGAGCTAGGGGTGCTGGAG
CGTTACTGTAAGTTTAACGCTCCTATCGACTACAGCGCCATACTGGACAAGACAGTTGAG
CCCCATGTCAGACTGAGCTCAGTGGCTCCCCCCAGTGGCAGACACCCTGCCATGCTGATG
TGCAGCGCCTACGACTTCTTCCCCAAACCAATCAGAGTGACCTGGCTGAGGGACGGACGT
GAGGTGAAGTCTGATGTGACCTCCACTGAGGAG
>AS8_DAB2_s1_41329
GTTTTGTCCATATTCTCTGGAACAGATGGATATTTTGAACAGGTTGTGAGACAGTGCCGA
TACTCCTCAAAGGACCTGCAGGGTATAGAGTTTATAGACTCTTATGTTTTCAATAAGGCT
GAATATGTCAGATTCAACAGCACTGTGGGGAAGTTTGTTGGATACACTGAGCTGGGAGTG
AAGAATGCAGAAGCCTGGAACAAAGGTCCTGAGCTGGCTGTAGAGCTAGGGGAGCTGGAG
CGTTACTGTAAGCTTAACGCTCCTATCGACTACAGCGCCATACTGGACAAGACAGTTGAG
CCCCATGTCAGACTGAGCTCAGTGGCTCCCCCCAGTGGCAGACACCCTGCCATGCTGATG
TGCAGCGCCTACGACTTCTACCCCAAACCAATCAGAGTGACCTGGCTGAGGGACGGACGT
GAGGTGAAGTCTGATGTGACCTCCACTGAGGAG
>AS8_DAB2_s2_29683
GTTTTGTCCATATTCTCTGGAACAGATGGATATTTTTATCAGAGGGTGTCAGAGTGCCGA

TACTCCTCAAAGGACCTGCAGGGTATAGAGTTTATAGACTCTTATGTTTTCAATAAGGCT
GAATATGTCAGATTCAACAGCACTGTGGGGAAGTATGTTGGATACACTGAGTATGGAGTG
AAGAATGCAGAAGCCTGGAACAAAGGTCCTGAGCTGGCTGGAGAGCTAGGGGAGCTGGAG
CGTGTCTGTAAGCATAACGCTCCTATCGACTACAGCGCCATACTGGACAAGACAGTTGAG
CCCCATGTCAGACTGAGCTCAGTGGCTCCCCCCAGTGGCAGACACCCTGCCATGCTGATG
TGCAGCGCCTACGACTTCTTCCCCAAACCAATCAGAGTGACCTGGCTGAGGGACGGACGT
GAGGTGAAGTCTGATGTGACCTCCACTGAGGAG
>AS8_DAB2_s3_7380
GTTTTGTCCATATTCTCTGGAACAGATGGATATTTTGAACAGGTTGTGAGACAGTGCCGA
TACTCCTCAAAGGACCTGCAGGGTATAGAGTTTATAGACTCTTATGTTTTCAATAAGGCT
GAATATGTCAGATTCAACAGCACTGTGGGGAAGTTTGTTGGATACACTGAGCTGGGAGTG
AAGAATGCAGAAGCCTGGAACAAAGGTCCTGAGCTGGCTGTAGAGCTAGGGGAGCTGGAG
CGTTACTGTAAGCTTAACGCTCCTATCGACTACAGCGCCATACTGGACAAGACAGTTGAG
CCCCATGTCAGACTGAGCTCAGTGGCTCCCCCCAGTGGCAGACACCCTGCCATGCTGATG
TGCAGCGCCTACGACTTCTTCCCCAAACCAATCAGAGTGACCTGGCTGAGGGACGGACGT
GAGGTGAAGTCTGATGTGACCTCCACTGAGGAG
>AS8_DAB2_s4_7176
GTTTTGTCCATATTCTCTGGAACAGATGGATATTTTTATCAGAGGGTGTCAGAGTGCCGA
TACTCCTCAAAGGACCTGCAGGGTATAGAGTTTATAGACTCTTATGTTTTCAATAAGGCT
GAATATGTCAGATTCAACAGCACTGTGGGGAAGTATGTTGGATACACTGAGTATGGAGTG
AAGAATGCAGAAGCCTGGAACAAAGGTCCTGAGCTGGCTGGAGAGCTAGGGGAGCTGGAG
CGTGTCTGTAAGCATAACGCTCCTATCGACTACAGCGCCATACTGGACAAGACAGTTGAG
CCCCATGTCAGACTGAGCTCAGTGGCTCCCCCCAGTGGCAGACACCCTGCCATGCTGATG
TGCAGCGCCTACGACTTCTACCCCAAACCAATCAGAGTGACCTGGCTGAGGGACGGACGT
GAGGTGAAGTCTGATGTGACCTCCACTGAGGAG
>AS8_DAB2_s5_5682
GTTTTGTCCATATTCTCTGGAACAGATGGATATTTTTATCAGAGGGTGTCAGAGTGCCGA
TACTCCTCAAAGGACCTGCAGGGTATAGAGTTTATAGACTCTTATGTTTTCAATAAGGCT
GAATATGTCAGATTCAACAGCACTGTGGGGAAGTTTGTTGGATACACTGAGCTGGGAGTG
AAGAATGCAGAAGCCTGGAACAAAGGTCCTGAGCTGGCTGTAGAGCTAGGGGAGCTGGAG
CGTTACTGTAAGCTTAACGCTCCTATCGACTACAGCGCCATACTGGACAAGACAGTTGAG
CCCCATGTCAGACTGAGCTCAGTGGCTCCCCCCAGTGGCAGACACCCTGCCATGCTGATG
TGCAGCGCCTACGACTTCTACCCCAAACCAATCAGAGTGACCTGGCTGAGGGACGGACGT
GAGGTGAAGTCTGATGTGACCTCCACTGAGGAG
>AS9_DAB1_s1_16301
GTTTCCCTGACCCTGGTTTTGTCCATATTCTCTGGAACAGATGGATATTTTGAACAGGTT
GTGAGACAGTGCCGATACTCCTCAAAGGACCTGCAGGGTATAGAGTTTATAGACTCTTAT
GTTTTCAATAAGGCTGAATATGTCAGATTCAACAGCACTGTGGGGAAGTATGTTGGATAC
ACTGAGCTGGGAGTGAAGAATGCAGAAGCCTGGAACAAAGGTCCTGAGCTGGCTGTAGAG
CTAGGGGAGCTGGAGCGTTTCTGTAAGCATAACGCTGATCTCCACTACCGCGCCATACTG
GACAAGACAGTTGAGCCCCATGTCAGACTGAGCTCAGTGGCTCCCCCCAGTGGCAGACAC
CCTGCCATGCTGATGTGCAGCGCCTACGACTTCTACCCCAAACCAATCAGAGTGACCTGG
CTGAGGGATGGACGTGAGGTGAAGTCTGATGTGACCTCCACTGAGGAG
>AS9_DAB1_s2_10525
GTTTCCCTGACCCTGGTTTTGTCCATATTCTCTGGAACAGATGGATATTTTTTTCAGGTT
GTGAGACAGTGCCGATACTCCTCAAAGGACCTGCAGGGTATAGAGTTTATACACTCTTAT
GTTTTCAATCAGGCTGAAAATATCAGATTCAACAGCACTGTGGGGAAGTATGTTGGATAC
ACTGAGCTGGGAGTGAAGAATGCAGAAGCCTGGAACAAAGGTCCTGAGCTGGCTGTAGAG
CTAGGGGAGCTGGAGCGTTACTGTAAGCATAACGCTGATATCGACTACAGCGCCATACTG
GACAAGACAGTTGAGCCCCATGTCAGACTGAGCTCAGTGGCTCCCCCCAGTGGCAGACAC
CCTGCCATGCTGATGTGCAGCGCCTACGACTTCTACCCCAAACCAATCAGAGTGACCTGG
CTGAGGGACGGACGTGAGGTGAAGTCTGATGTGACCTCCACTGAGGAG
>AS9_DAB1_s3_2669
GTTTCCCTGACCCTGGTTTTGTCCATATTCTCTGGAACAGATGGATATTTTGAACAGGTT
GTGAGACAGTGCCGATACTCCTCAAAGGACCTGCAGGGTATAGAGTTTATAGACTCTTAT
GTTTTCAATAAGGCTGAATATGTCAGATTCAACAGCACTGTGGGGAAGTATGTTGGATAC
ACTGAGCTGGGAGTGAAGAATGCAGAAGCCTGGAACAAAGGTCCTGAGCTGGCTGTAGAG
CTAGGGGAGCTGGAGCGTTTCTGTAAGCATAACGCTGATCTCCACTACCGCGCCATACTG
GACAAGACAGTTGAGCCCCATGTCAGACTGAGCTCAGTGGCTCCCCCCAGTGGCAGACAC
CCTGCCATGCTGATGTGCAGCGCCTACGACTTCTACCCCAAACCAATCAGAGTGACCTGG
CTGAGGGACGGACGTGAGGTGAAGTCTGATGTGACCTCCACTGAGGAG

```
>AS9_DAB1_s4_2356
GTTTCCCTGACCCTGGTTTTGTCCATATTCTCTGGAACAGATGGATATTTTTTTCAGGTT
GTGAGACAGTGCCGATACTCCTCAAAGGACCTGCAGGGTATAGAGTTTATACACTCTTAT
GTTTTCAATCAGGCTGAAAATATCAGATTCAACAGCACTGTGGGGAAGTATGTTGGATAC
ACTGAGCTGGGAGTGAAGAATGCAGAAGCCTGGAACAAAGGTCCTGAGCTGGCTGTAGAG
CTAGGGGAGCTGGAGCGTTACTGTAAGCATAACGCTGATATCGACTACAGCGCCATACTG
GACAAGACAGTTGAGCCCCATGTCAGACTGAGCTCAGTGGCTCCCCCCAGTGGCAGACAC
CCTGCCATGCTGATGTGCAGCGCCTACGACTTCTACCCCAAACCAATCAGAGTGACCTGG
CTGAGGGATGGACGTGAGGTGAAGTCTGATGTGACCTCCACTGAGGAG
>AS9_DAB1_s5_2051
GTTTCCCTGACCCTGGTTTTGTCCATATTCTCTGGAACAGATGGATATTTTGAACAGGTT
GTGAGACAGTGCCGATACTCCTCAAAGGACCTGCAGGGTATAGAGTTTATAGACTCTTAT
GTTTTCAATAAGGCTGAATATGTCAGATTCAACAGCACTGTGGGGAAGTATGTTGGATAC
ACTGAGCTGGGAGTGAAGAATGCAGAAGCCTGGAACAAAGGTCCTGAGCTGGCTGTAGAG
CTAGGGGAGCTGGAGCGTTACTGTAAGCATAACGCTGATATCGACTACAGCGCCATACTG
GACAAGACAGTTGAGCCCCATGTCAGACTGAGCTCAGTGGCTCCCCCCAGTGGCAGACAC
CCTGCCATGCTGATGTGCAGCGCCTACGACTTCTACCCCAAACCAATCAGAGTGACCTGG
CTGAGGGACGGACGTGAGGTGAAGTCTGATGTGACCTCCACTGAGGAG
>AS10_DAB1_s1_74482
GTTTCCCTGACCCTGGTTTTGTCCATATTCTCTGGAACAGATGGATATTTTGAACAGGTT
GTGAGACAGTGCCGATACTCCTCAAAGGACCTGCAGGGTATAGAGTTTATAGACTCTTAT
GTTTTCAATAAGGCTGAATATGTCAGATTCAACAGCACTGTGGGGAAGTATGTTGGATAC
ACTGAGCTGGGAGTGAAGAATGCAGAAGCCTGGAACAAAGGTCCTGAGCTGGCTGTAGAG
CTAGGGGAGCTGGAGCGTTACTGTAAGCTTAACGCTCCTATCGACTACAGCGCCATACTG
GACAAGACAGTTGAGCCCCATGTCAGACTGAGCTCAGTGGCTCCCCCCAGTGGCAGACAC
CCTGCCATGCTGATGTGCAGCGCCTACGACTTCTACCCCAAACCAATCAGAGTGACCTGG
CTGAGGGACGGACGTGAGGTGAAGTCTGATGTGACCTCCACTGAGGAG
>AS10_DAB1_s2_380
GTTTCCCTGACCCTGGTTTTGTCCATATTCTCTGGAACAGATGGATATTTTGAACAGGTT
GTGAGACAGTGCCGATACTCCTCAAAGGACCTGCAGGGTATAGAGTTTATAGACTCTTAT
GTTTTCAATAAGGCTGAATATGTCAGATTCAACAGCACTGTGGGGAAGTATGTTGGATAC
ACTGAGCTGGGAGTGAAGAATGCAGAAGCCTGGAACAAAGGTCCTGAGCTGGCTGTAGAG
CTAGGGGAGCTGGAGCGTTACTGTAAGCTTAACGCTCCTATCGACTACAGCGCCATACTG
GACAAGACAGTTGAGCCCCATGTCAGACTGAGCTCAGTGGCCCCCCCCAGTGGCAGACAC
CCTGCCATGCTGATGTGCAGCGCCTACGACTTCTACCCCAAACCAATCAGAGTGACCTGG
CTGAGGGACGGACGTGAGGTGAAGTCTGATGTGACCTCCACTGAGGAG
>AS10_DAB1_s3_353
GTTTCCCTGACCCTGGTTTTGTCCATATTCTCTGGAACAGATGGATATTTTGAACAGGTT
GTGAGACAGTGCCGATACTCCTCAAAGGACCTGCAGGGTATAGAGTTTATAGACTCTTAT
GTTTTCAATAAGGCTGAATATGTCAGATTCAACAGCACTGTGGGGAAGTATGTTGGATAC
ACTGAGCTGGGAGTGAAGAATGCAGAAGCCTGGAACAAAGGTCCTGAGCTGGCTGTAGAG
CTAGGGGAGCTGGAGCGTTACTGTAAGCTTAACGCTCCTATCGACTACAGCGCCATACTG
GACAAGACAGTTGAGCCCCATGTCAGACTGGGCTCAGTGGCTCCCCCCAGTGGCAGACAC
CCTGCCATGCTGATGTGCAGCGCCTACGACTTCTACCCCAAACCAATCAGAGTGACCTGG
CTGAGGGACGGACGTGAGGTGAAGTCTGATGTGACCTCCACTGAGGAG
>AS10_DAB1_s4_342
GTTTCCCTGACCCTGGTTTTGTCCATATTCTCTGGAACAGATGGATATTTTGAACAGGTT
GTGAGACAGTGCCGATACTCCTCAAAGGACCTGCAGGGTATAGAGTTTATAGACTCTTAT
GTTTTCAATAAGGCTGAATATGTCAGATTCAACAGCACTGTGGGGAAGTATGTTGGATAC
ACTGAGCTGGGAGTGAAGAATGCAGAAGCCTGGAACAAAGGTCCTGAGCTGGCTGTAGAG
CTAGGGGAGCTGGAGCGTTACTGTAAGCTTAACGCTCCTATCGACTACAGCGCCATACTG
GACAAGACAGTTGAGCCCCATGTCAGACTGAGCTCAGTGGCTCCCCCCAGTGGCAGACAC
CCTGCCATGCTGATGTGCAGCGCCTACGACTTCTACCCCAAACCAATCAGAGTGACCTGG
CTGAGGGACGGACGTGGGGTGAAGTCTGATGTGACCTCCACTGAGGAG
>AS10_DAB1_s5_337
GTTTCCCTGACCCTGGTTTTGTCCATATTCTCTGGAACAGATGGATATTTTGAACAGGTT
GTGAGACAGTGCCGATACTCCTCAAAGGACCTGCAGGGTATAGAGTTTATAGACTCTTAT
GTTTTCAATAAGGCTGAATATGTCAGATTCAACAGCACTGTGGGGAAGTATGTTGGATAC
ACTGAGCTGGGAGTGAAGAATGCAGAAGCCTGGAACAAAGGTCCTGAGCTGGCTGTAGAG
CTAGGGGAGCTGGAGCGTTACTGTAAGCTTAACGCTCCTATCGACTACAGCGCCATACTG
GACAAGACAGTTGAGCCCCATGTCAGACTGAGCTCAGTGGCTCCCCCCAGTGGCAGACAC
```

CCTGCCATGCTGATGTGCAGCGCCTACGACTTCTACCCCAAACCAATCAGAGTGGCCTGG
CTGAGGGACGGACGTGAGGTGAAGTCTGATGTGACCTCCACTGAGGAG

MHC class I alpha (UBA) nucleotide sequences:

>AS1_UBA1_s1_30927
ACAGCATCTGCTGCCACCCACTCTCTGAAATACTTCTACACAGCAGTCTCAGGTGATATT
GACTTCCCAGAGTTCACCATCGTTGGTCTGGTCAACAATGGCCAGTTTGTGTACTATGAC
AGCAACATAAAGAGGATGGTCCCCAAGACTGAGTGGATGAAGCAGAGCGCAGGAGCAGAT
TACTGGGACACGGAGAGTGAGAAGCAAGTTGGTCAAAATCAAGGCTTCAAAAACAACATT
CAAGTGCTAAAGGATCGCTTCAACCAGTCCATGTCCACAGGTGTGCACGTTTTCCAGGTG
ATGTATGGATGTGAGTGGGATGATGAGGCTGGAGCCACAGAGGGGTTTGATCAGTATGGA
TATGATGGAGAGGATTTCTTAGCATTTGACCTGAAGACATTGAAATGGATCGCCCCAACG
CCACAGGCAGTCATCACCAAACTCAAGTGGGACAGTGACACAGCTCAGAATGAGTACCGA
AAAAACTACTACACCCAGACCTGCATTGAG
>AS1_UBA1_s2_203
ACAGCATCTGCTGCCACCCACTCTCTGAAATACTTCTACACAGCAGTCTCAGGTGTGCAC
GTTTTCCAGGTGATGTATGGATGTGAGTGGGATGATGAGGCTGGAGCCACAGAGGGGTTT
GATCAGTATGGATATGATGGAGAGGATTTCTTAGCATTTGACCTGAAGACATTGAAATGG
ATCGCCCCAACGCCACAGGCAGTCATCACCAAACTCAAGTGGGACAGTGACACAGCTCAG
AATGAGTACCGAAAAAACTACTACACCCAGACCTGCATTGAG
>AS1_UBA1_s3_169
ACAGCATCTGCTGCCACCCACTCTCTGAAATACTTCTACACAGCAGTCTCAGGTGATATT
GACTTCCCAGAGTTCACCATCGTTGGTCTGGTCAACAATGGCCAGTTTGTGTACTATGAC
AGCAACATAAAGAGGATGGTCCCCAAGACTGAGTGGATGAAGCAGAGCGCAGGAGCAGAT
TACTGGGACACGGAGAGTGAGAAGCAAGTTGGTCAAAATCAAGGCTTCAAAAACAACATT
CAAGTGCTAAAGGATCGCTTCAACCAGTCCATGTCCACAGGTGTGCACGTTTGCCAGGTG
ATGTATGGATGTGAGTGGGATGATGAGGCTGGAGCCACAGAGGGGTTTGATCAGTATGGA
TATGATGGAGAGGATTTCTTAGCATTTGACCTGAAGACATTGAAATGGATCGCCCCAACG
CCACAGGCAGTCATCACCAAACTCAAGTGGGACAGTGACACAGCTCAGAATGAGTACCGA
AAAAACTACTACACCCAGACCTGCATTGAG
>AS1_UBA1_s4_161
ACAGCATCTGCTGCCACCCACTCTCTGAAATACTTCTACACAGCAGTCTCAGGTGATATT
GACTTCCCAGAGTTCACCATCGTTGGTCTGGTCAACAATGGCCAGTTTGTGTACTATGAC
AGCAACATAAAGAGGATGGTCCCCAAGACTGAGTGGATGAAGCAGAGCGCAGGAGCAGAT
TACTGGGACACGGAGAGTGAGAAGCAAGTTGGTCAAAATCAAGGCTTCAAAAACAACATT
CAAGTGCTAAAGGATCGCTTCAACCAGTCCATGTCCACAGGTGTGCACGTTTTCCAGGTG
ATGTATGGATGTGAGTGGGATGATGAGGCTGGAGCCACAGAGGGGTTTGATCAGTATGGA
TATGATGGAGAGGATTTCTTAGCATTTGACCTGAAGACATTGAAATGGATCGCCCCAACG
CCACAGGCAGTCATCACCAAACTCAAGTGGGACAGTGGCACAGCTCAGAATGAGTACCGA
AAAAACTACTACACCCAGACCTGCATTGAG
>AS1_UBA1_s5_153
ACAGCATCTGCTGCCACCCACTCTCTGAAATACTTCTACACAGCAGTCTCAGGTGATATT
GACTTCCCAGAGTTCACCATCGTTGGTCTGGTCAACAATGGCCAGTTTGTGTACTATGAC
AGCAACATAAAGAGGATGGTCCCCAAGACTGAGTGGATGAAGCAGAGCGCAGGAGCAGAT
TACTGGGACACGGAGAGTGGGAAGCAAGTTGGTCAAAATCAAGGCTTCAAAAACAACATT
CAAGTGCTAAAGGATCGCTTCAACCAGTCCATGTCCACAGGTGTGCACGTTTTCCAGGTG
ATGTATGGATGTGAGTGGGATGATGAGGCTGGAGCCACAGAGGGGTTTGATCAGTATGGA
TATGATGGAGAGGATTTCTTAGCATTTGACCTGAAGACATTGAAATGGATCGCCCCAACG
CCACAGGCAGTCATCACCAAACTCAAGTGGGACAGTGACACAGCTCAGAATGAGTACCGA
AAAAACTACTACACCCAGACCTGCATTGAG
>AS2_UBA1_s1_46437
ACAGCATCTGCTGTGACTCACGCCCTGAAGTATTTCTACACCGCATCTTCTGAAGTTCCC
AACTTCCCAGAGTTTGTAGTTGTGGCGATGGTGGATGGTGTTCAGATGGTTCACTATGAC
AGCAACAGCCAGAGAGCGGTGCCCAAACAGGACTGGGTAAACAAGGCAGCAGACCCACAG
TACTGGGAGAGGAACACTGGGATTTTCAAGGGTTCCCAGCAGACTTTCAAAGCCAACATC
GATATTGTAAAGCAGCGTTTTAACCAAAGTGGAGGTGTGCACATTTACCAGAATATGTAT
GGATGTGAGTGGGATGATGAGGCTGGAGTCACAGAGGGGTTTGATCAGTATGGATATGAT
GGAGAGGATTTCTTAGCATTTGACCTGAAGACATTGAAATGGATCGCCCCAACGCCACAG
TCACTCATCACCAAACTCAAGTGGGATAATAACATGGCTCAGATACAGCAGGATAAACAT
TACCTCACCCAGACCTGCATTGAG

>AS2_UBA1_s2_33647
ACAGCATCTGCTGTGACTCACGCCCTGAAGTATTTCTACACCGCATCTTCTGAAGTTCCC
AACTTCCCAGAGTTTGTAGTTGTGGGGGTGGTGGATGGTGTTCAGATGGTTCACTATGAC
AGCAACAGCCAGAGAGCGGTGCCCAAACAGGACTGGGTAAACAAGGCAGCAGACCCACAG
TACTGGGAGAGGAACACTGGGAATTGCAAGGGTTCCCAGCAGATTTTCAAAGCCAACATC
GATATTGTAAAGCAGCGTTTTAACCAAAGTGGAGGTGTGCACGTTAACCAGAATATGTAT
GGATGTGAGTGGGATGATGAGGCTGGAGTCACAGAGGGGTTTGATCAGTATGGATATGAT
GGAGAGGATTTCTTAGCATTTGACCTGAAGACATTGAAATGGATCGCCCCAACGCCACAG
TCACTCATCACCAAACTCAAGTGGGATAATAACATGGCTCAGATACAGCAGGATAAACAT
TACCTCACCCAGACCTGCATTGAG
>AS2_UBA1_s3_8559
ACAGCATCTGCTGTGACTCACGCCCTGAAGTATTTCTACACCGCATCTTCTGAAGTTCCC
AACTTCCCAGAGTTTGTAGTTGTGGCGATGGTGGATGGTGTTCAGATGGTTCACTATGAC
AGCAACAGCCAGAGAGCGGTGCCCAAACAGGACTGGGTAAACAAGGCAGCAGACCCACAG
TACTGGGAGAGGAACACTGGGAATTGCAAGGGTTCCCAGCAGATTTTCAAAGCCAACATC
GATATTGTAAAGCAGCGTTTTAACCAAAGTGGAGGTGTGCACGTTAACCAGAATATGTAT
GGATGTGAGTGGGATGATGAGGCTGGAGTCACAGAGGGGTTTGATCAGTATGGATATGAT
GGAGAGGATTTCTTAGCATTTGACCTGAAGACATTGAAATGGATCGCCCCAACGCCACAG
TCACTCATCACCAAACTCAAGTGGGATAATAACATGGCTCAGATACAGCAGGATAAACAT
TACCTCACCCAGACCTGCATTGAG
>AS2_UBA1_s4_7940
ACAGCATCTGCTGTGACTCACGCCCTGAAGTATTTCTACACCGCATCTTCTGAAGTTCCC
AACTTCCCAGAGTTTGTAGTTGTGGGGGTGGTGGATGGTGTTCAGATGGTTCACTATGAC
AGCAACAGCCAGAGAGCGGTGCCCAAACAGGACTGGGTAAACAAGGCAGCAGACCCACAG
TACTGGGAGAGGAACACTGGGATTTTCAAGGGTTCCCAGCAGACTTTCAAAGCCAACATC
GATATTGTAAAGCAGCGTTTTAACCAAAGTGGAGGTGTGCACATTTACCAGAATATGTAT
GGATGTGAGTGGGATGATGAGGCTGGAGTCACAGAGGGGTTTGATCAGTATGGATATGAT
GGAGAGGATTTCTTAGCATTTGACCTGAAGACATTGAAATGGATCGCCCCAACGCCACAG
TCACTCATCACCAAACTCAAGTGGGATAATAACATGGCTCAGATACAGCAGGATAAACAT
TACCTCACCCAGACCTGCATTGAG
>AS2_UBA1_s5_4195
ACAGCATCTGCTGTGACTCACGCCCTGAAGTATTTCTACACCGCATCTTCTGAAGTTCCC
AACTTCCCAGAGTTTGTAGTTGTGGCGATGGTGGATGGTGTTCAGATGGTTCACTATGAC
AGCAACAGCCAGAGAGCGGTGCCCAAACAGGACTGGGTAAACAAGGCAGCAGACCCACAG
TACTGGGAGAGGAACACTGGGATTTTCAAGGGTTCCCAGCAGACTTTCAAAGCCAACATC
GATATTGTAAAGCAGCGTTTTAACCAAAGTGGAGGTGTGCACGTTAACCAGAATATGTAT
GGATGTGAGTGGGATGATGAGGCTGGAGTCACAGAGGGGTTTGATCAGTATGGATATGAT
GGAGAGGATTTCTTAGCATTTGACCTGAAGACATTGAAATGGATCGCCCCAACGCCACAG
TCACTCATCACCAAACTCAAGTGGGATAATAACATGGCTCAGATACAGCAGGATAAACAT
TACCTCACCCAGACCTGCATTGAG
>AS3_UBA1_s1_13465
ACAGCATCTGCTGCCACCCACTCTCTGAAATACTTCTACACAGCAGTCTCAGGTGATATT
GACTTCCCAGAGTTCACCATCGTTGGTCTGGTCAACAATGGCCAGTTTGTGTACTATGAC
AGCAACATAAAGAGGATGGTCCCCAAGACTGAGTGGATGAAGCAGAGCGCAGGAGCAGAT
TACTGGGACACGGAGAGTGAGAAGCAAGTTGGTCAAAATCAAGGCTTCAAAAACAACATT
CAAGTGCTAAAGGATCGCTTCAACCAGTCCATGTCCACAGGTGTGCACGTTTTCCAGGTG
ATGTATGGATGTGAGTGGGATGATGAGGCTGGAGCCACAGAGGGGTTTGATCAGTATGGA
TATGATGGAGAGGATTTCTTAGCATTTGACCTGAAGACATTGAAATGGATCGCCCCAACG
CCACAGGCAGTCATCACCAAACTCAAGTGGGACAGTGACACAGCTCAGAATGAGTACCGA
AAAAACTACTACACCCAGACCTGCATTGAG
>AS3_UBA1_s2_12935
ACAGCATCTGCTGTGACTCACTCCCTGAAGTATTTCTACACCGCATCTTCTGAAGTTCCC
AACTTCCCAGAGTTTGTAGTTGTGTCAATGGTGGATGGTGTTCAGATGGTTCACTATGAC
AGCAACAGCCAGAGAGCGGTGCCAAAACAGGACTGGATGAACAAGGCAGCAGAAGCACTG
CCACAGTACTGGGATATCGAGACAGGGAAATTTTTGGGTTCCCATCAGAGTTTCAAAGCC
AACATCGATATTTGTAAGCAGCGTTTTAACCAAAGTGGAGGTGTGCACATTGTCCAGAAG
ATGTATGGATGTGAGTGGGATGATGAGACTGGAGTCACAGAGGGGTTTAATCAGTATGGA
TATGATGGAGAGGATTTCATAGCATTTGACCTGAAGACATTGAAATGGATCGCCCCAACG
CCACAGGCAGTCATCACCAAACTCAAGTGGGACAGTGACACAGCTCAGAATGAGTACCGA
AAAAACTACTACACCCAGACCTGCATTGAG
>AS3_UBA1_s3_609

```
ACAGCATCTGCTGTGACTCACTCCCTGAAGTATTTCTACACCGCATCTTCTGAAGTTCCC
AACTTCCCAGAGTTTGTAGTTGTGTCAATGGTGGATGGTGTTCAGATGGTTCACTATGAC
AGCAACAGCCAGAGAGCGGTGCCAAAACAGGACTGGATGAACAAGGCAGCAGAAGCACTG
CCACAGTACTGGGATATCGAGACAGGGAAATTTTTGGGTTCCCATCAGAGTTTCAAAGCC
AACATCGATATTTGTAAGCAGCGTTTTAACCAAAGTGGAGGTGTGCACATTGTCCAGAAG
ATGTATGGATGTGAGTGGGATGATGAGACTGGAGTCACAGAGGGGTTTAATCAGTATGGA
TATGATGGAGAGGATTTCTTAGCATTTGACCTGAAGACATTGAAATGGATCGCCCCAACG
CCACAGGCAGTCATCACCAAACTCAAGTGGGACAGTGACACAGCTCAGAATGAGTACCGA
AAAAACTACTACACCCAGACCTGCATTGAG
>AS3_UBA1_s4_609
ACAGCATCTGCTGCCACCCACTCTCTGAAATACTTCTACACAGCAGTCTCAGGTGATATT
GACTTCCCAGAGTTCACCATCGTTGGTCTGGTCAACAATGGCCAGTTTGTGTACTATGAC
AGCAACATAAAGAGGATGGTCCCCAAGACTGAGTGGATGAAGCAGAGCGCAGGAGCAGAT
TACTGGGACACGGAGAGTGAGAAGCAAGTTGGTCAAAATCAAGGCTTCAAAAACAACATT
CAAGTGCTAAAGGATCGCTTCAACCAGTCCATGTCCACAGGTGTGCACGTTTTCCAGGTG
ATGTATGGATGTGAGTGGGATGATGAGGCTGGAGCCACAGAGGGGTTTGATCAGTATGGA
TATGATGGAGAGGATTTCATAGCATTTGACCTGAAGACATTGAAATGGATCGCCCCAACG
CCACAGGCAGTCATCACCAAACTCAAGTGGGACAGTGACACAGCTCAGAATGAGTACCGA
AAAAACTACTACACCCAGACCTGCATTGAG
>AS3_UBA1_s5_288
ACAGCATCTGCTGTGACTCACTCCCTGAAGTATTTCTACACCGCATCTTCTGAAGTTCCC
AACTTCCCAGAGTTTGTAGTTGTGTCAATGGTGGATGGTGTTCAGATGGTTCACTATGAC
AGCAACAGCCAGAGAGCGGTGCCAAAACAGGACTGGATGAACAAGGCAGCAGAAGCACTG
CCACAGTACTGGGATATCGAGACAGGGAAATTTTTGGGTTCCCATCAGAGTTTCAAAGCC
AACATCGATATTTGTAAGCAGCGTTTTAACCAAAGTGGAGGTGTGCACATTGTCCAGAAG
ATGTATGGATGTGAGTGGGATGATGAGGCTGGAGCCACAGAGGGGTTTGATCAGTATGGA
TATGATGGAGAGGATTTCTTAGCATTTGACCTGAAGACATTGAAATGGATCGCCCCAACG
CCACAGGCAGTCATCACCAAACTCAAGTGGGACAGTGACACAGCTCAGAATGAGTACCGA
AAAAACTACTACACCCAGACCTGCATTGAG
>AS5_UBA2_s1_22247
CTGAAGTATTTCTACACTGGAAGTACTGGCATTGAGGGGTTCCCACAGTTTGTTGCAGTG
GGCATCGTAGATGGAATGCACATTGACTACTTTGATAGTGTGTCTGAGAAGAATGTTCTG
AAGCAGTCTTGGATGGAGGGGGCACGTGATGAAAAAAGCATCACAAACATAAGAAAGGGC
AATCAGCAAAGCTTTAAGGCCAATGTCGAAATCGTAATGCAACGTTTCAACCAAACAACA
GGTGTGCACGTTTTCCAGAATATGTATGGATGTGAGTGGGATGATGAGACTGGAGTCACA
GAGGGGTTTGATCAGGATGGATATGATGGAGAGGATTTCTTAGCATTTGACCTGAAGACA
TTGACATGGATCGCCCCAACGCCACAGGCAGTCAACACCAAACACAAGTGGGACAGTAAC
ACAGCTTACAATGAGCAGGAAAAAAACTACCTCACCCAGATCTGCATTGAG
>AS5_UBA2_s2_20661
CTGAAGTATTTCTACACTGGAAGTACTGGCATTGAGGGGTTCCCACAGTTTGTTGCAGTG
GGCATCGTAGATGGAATGCACATTGACTACTTTGATAGTGTGTCTGAGAAGAATGTTCTG
AAGCAGTCTTGGATGGAGGGGGCACGTGATGAAAAAAGCATCACAAACATAAGAAAGGGC
AATCAGCAAAGCTTTAAGGCCAATGTCGAAATCGTAATGCAACGTTTCAACCAAACAACA
GGTGTGCACGTTAACCAGTGGATGTATGGATGTGAGTGGGATGATGAGGCTGGAGTCACA
GAGGGGTTTGAACAGTGGGGATATGATGGAGAGGACTTCATAGCATTTGACCTGAAGACA
AAGTCATGGATCGCCCCAACGCCACAGGCAGTCATCACCAAACTCAAGTGGGACAGTGAC
ACAGCTCAGAATGAGCACCGAAAAAACTACTACACCCAGATCTGCATTGAG
>AS5_UBA2_s3_576
CTGAAGTATTTCTACACTGGAAGTACTGGCATTGAGGGGTTCCCACAGTTTGTTGCAGTG
GGCATCGTAGATGGAATGCACATTGACTACTTTGATAGTGTGTCTGAGAAGAATGTTCTG
AAGCAGTCTTGGATGGAGGGGGCACGTGATGAAAAAAGCATCACAAACATAAGAAAGGGC
AATCAGCAAAGCTTTAAGGCCAATGTCGAAATCGTAATGCAACGTTTCAACCAAACAACA
GGTGTGCACGTTTTCCAGAATATGTATGGATGTGAGTGGGATGATGAGACTGGAGTCACA
GAGGGGTTTGATCAGGATGGATATGATGGAGAGGATTTCTTAGCATTTGACCTGAAGACA
TTGACATGGATCGCCCCAACGCCACAGGCAGTCATCACCAAACTCAAGTGGGACAGTGAC
ACAGCTCAGAATGAGCACCGAAAAAACTACTACACCCAGATCTGCATTGAG
>AS5_UBA2_s4_489
CTGAAGTATTTCTACACTGGAAGTACTGGCATTGAGGGGTTCCCACAGTTTGTTGCAGTG
GGCATCGTAGATGGAATGCACATTGACTACTTTGATAGTGTGTCTGAGAAGAATGTTCTG
AAGCAGTCTTGGATGGAGGGGGCACGTGATGAAAAAAGCATCACAAACATAAGAAAGGGC
AATCAGCAAAGCTTTAAGGCCAATGTCGAAATCGTAATGCAACGTTTCAACCAAACAACA
```

GGTGTGCACGTTTTCCAGAATATGTATGGATGTGAGTGGGATGATGAGACTGGAGTCACA
GAGGGGTTTGAACAGTGGGGATATGATGGAGAGGACTTCATAGCATTTGACCTGAAGACA
AAGTCATGGATCGCCCCAACGCCACAGGCAGTCATCACCAAACTCAAGTGGGACAGTGAC
ACAGCTCAGAATGAGCACCGAAAAAACTACTACACCCAGATCTGCATTGAG
>AS5_UBA2_s5_480
CTGAAGTATTTCTACACTGGAAGTACTGGCATTGAGGGGTTCCCACAGTTTGTTGCAGTG
GGCATCGTAGATGGAATGCACATTGACTACTTTGATAGTGTGTCTGAGAAGAATGTTCTG
AAGCAGTCTTGGATGGAGGGGGCACGTGATGAAAAAAGCATCACAAACATAAGAAAGGGC
AATCAGCAAAGCTTTAAGGCCAATGTCGAAATCGTAATGCAACGTTTCAACCAAACAACA
GGTGTGCACGTTAACCAGTGGATGTATGGATGTGAGTGGGATGATGAGGCTGGAGTCACA
GAGGGGTTTGATCAGGATGGATATGATGGAGAGGATTTCTTAGCATTTGACCTGAAGACA
TTGACATGGATCGCCCCAACGCCACAGGCAGTCAACACCAAACACAAGTGGGACAGTAAC
ACAGCTTACAATGAGCAGGAAAAAAACTACCTCACCCAGATCTGCATTGAG
>AS6_UBA1_s1_18686
ACAGCATCTGCTGTGACTCACTCCCTGAAGTATTTCTACACCGCATCTTCTGAAGTTCCC
AACTTCCCAGAGTTTGTAGTTGTGTCAATGGTGGATGGTGTTCAGATGGTTCACTATGAC
AGCAACAGCCAGAGAGCGGTGCCAAAACAGGACTGGATGAACAAGGCAGCAGAAGCACTG
CCACAGTACTGGGATATCGAGACAGGGAAATTTTTGGGTTCCCATCAGAGTTTCAAAGCC
AACATCGATATTTGTAAGCAGCGTTTTAACCAAAGTGGAGGTGTGCACATTGTCCAGAAG
ATGTATGGATGTGAGTGGGATGATGAGACTGGAGTCACAGAGGGGTTTAATCAGTATGGA
TATGATGGAGAGGATTTCATAGCATTTGACCTGAAGACATTGAAATGGATCGCCCCAACG
CCACAGGCAGTCATCACCAAACTCAAGTGGGACAGTGACACAGCTCAGAATGAGTACCGA
AAAAACTACTACACCCAGACCTGCATTGAG
>AS6_UBA1_s2_3956
ACAGTATCTGCTGCAACCAATACACTGCAGTATTTCTACACGGCCACTTCTGGTATAGAT
AACTTCCCAGAGTTTGTGACTATGGGAATCGTCAATGGCCATCAGATTGACCACTACGAC
AGTATCACCAAGAGAGCAATCCAGAAAGCTGAGTGGATCAGTGGGGCAGTTGACCCTGAC
TATTGGAAGACTAACACCCAGATCTATGCTGGTACAGAGACAGTTTTCGTGAACAACATT
AATGTTGCGAAATCCCGCTTCAATCAAACTGGAGGTGTGCACGTTAACCAGAAGATGTAT
GGATGTGAGTGGGATGATGAGACTGGAGTCACAGAGGGGTTTGATCAGGATGGATATGAT
GGAGAGGATTTCTTAGCATTTGACCTGAAAACATTGACATGGATCGCCCCAACGCCACAG
GCAGTCATCACCAAACTCAAGTGGGACAGTAACACAGCTCAGAATGAGTACCGAAAAAAC
TACCTCACCCAGACCTGCATTGAG
>AS6_UBA1_s3_399
ACAGTATCTGCTGCAACCAATACACTGCAGTATTTCTACACGGCCACTTCTGGTATAGAT
AACTTCCCAGAGTTTGTGACTATGGGAATCGTCAATGGCCATCAGATTGACCACTACGAC
AGTATCACCAAGAGAGCAATCCAGAAAGCTGAGTGGATCAGTGGGGCAGTTGACCCTGAC
TATTGGAAGACTAACACCCAGATCTATGCTGGTACAGAGACAGTTTTCGTGAACAACATT
AATGTTGCGAAATCCCGCTTCAATCAAACTGGAGGTGTGCACGTTAACCAGAAGATGTAT
GGATGTGAGTGGGATGATGAGACTGGAGTCACAGAGGGGTTTGATCAGGATGGATATGAT
GGAGAGGATTTCTTAGCATTTGACCTGAAAACATTGACATGGATCGCCCCAACGCCACAG
GCAGTCATCACCAAACTCAAGTGGGACAGTGACACAGCTCAGAATGAGTACCGAAAAAAC
TACTACACCCAGACCTGCATTGAG
>AS6_UBA1_s4_297
ACAGCATCTGCTGTGACTCACTCCCTGAAGTATTTCTACACCGCATCTTCTGAAGTTCCC
AACTTCCCAGAGTTTGTAGTTGTGTCAATGGTGGATGGTGTTCAGATGGTTCACTATGAC
AGCAACAGCCAGAGAGCGGTGCCAAAACAGGACTGGATGAACAAGGCAGCAGAAGCACTG
CCACAGTACTGGGATATCGAGACAGGGAAATTTTTGGGTTCCCATCAGAGTTTCAAAGCC
AACATCGATATTTGTAAGCAGCGTTTTAACCAAAGTGGAGGTGTGCACATTGTCCAGAAG
ATGTATGGATGTGAGTGGGATGATGAGACTGGAGTCACAGAGGGGTTTAATCAGTATGGA
TATGATGGAGAGGATTTCATAGCATTTGACCTGAAGACATTGAAATGGATCGCCCCAACG
CCACAGGCAGTCATCACCAAACTCAAGTGGGACAGTGACACAGCTCAGAATGAGTACCGA
AAAAACTACCTCACCCAGACCTGCATTGAG
>AS6_UBA1_s5_287
ACAGTATCTGCTGCAACCAATACACTGCAGTATTTCTACACGGCCACTTCTGGTATAGAT
AACTTCCCAGAGTTTGTGACTATGGGAATCGTCAATGGCCATCAGATTGACCACTACGAC
AGTATCACCAAGAGAGCAATCCAGAAAGCTGAGTGGATCAGTGGGGCAGTTGACCCTGAC
TATTGGAAGACTAACACCCAGATCTATGCTGGTACAGAGACAGTTTTCGTGAACAACATT
AATGTTGCGAAATCCCGCTTCAATCAAACTGGAGGTGTGCACGTTAACCAGAAGATGTAT
GGATGTGAGTGGGATGATGAGACTGGAGTCACAGAGGGGTTTGATCAGGATGGATATGAT
GGAGAGGATTTCTTAGCATTTGACCTGAAAACATTGACATGGATCGCCCCAACGCCACAG

GCAGTCATCACCAAACTCAAGTGGGACAGTAACACAGCTCAGAATGAGTACCGAAAAAAC
TACTACACCCAGACCTGCATTGAG
>AS7_UBA1_s1_25193
ACAGCATCTGCTGTGACTCACGCCCTGAAGTATTTCTACACCGCATCTTCTGAAGTTCCC
AACTTCCCAGAGTTTGTGGCTGTGGGGGTGGTGGATGGTGTTCAGATGTTTCACTATGAC
AGCAACAGCCAGAGAGCGGTGCCCAAACAGGACTGGGTAAACAAGGCAGCAGACCCACAG
TACTGGGAGAGGAACACTGGGAATTGCAAGGGTTCCCAGCAGATTTTCAAAGCCAACATC
GATATTGTAAAGCAGCGTTTTAACCAAAGTGGAGGTGTGCACGTTTTCCAGAATATGTAT
GGATGTGAGTGGGATGATGAGGCTGGAGTCACAGAGGGGTTTGATCAGTATGGATATGAT
GGAGAGGATTTCATAGCATTTGACCTGAAGACAAAGACATGGATCGCCCCAAAGACACAG
GCAGTCAACACCAAAAACAAGTGGGACAGTGACACAGCTCAGAATGAGTTCCTAAAAAAC
TACTACACCCAGACCTGCATTGAG
>AS7_UBA1_s2_6454
ACAGTATCTGCTGCAACCAATACACTGCAGTATTTCTACACGGCCACTTCTGGTATAGAT
AACTTCCCAGAGTTTGTGACTATGGGAATCGTCAATGGCCATCAGATTGACCACTACGAC
AGTATCACCAAGAGAGCAATCCAGAAAGCTGAGTGGATCAGTGGGGCAGTTGACCCTGAC
TATTGGAAGACTAACACCCAGATCTATGCTGGTACAGAGACAGTTTTCGTGAACAACATT
AATGTTGCGAAATCCCGCTTCAATCAAACTGGAGGTGTGCACGTTTTCCAGATGATGTGT
GGTTGTGAGTGGGATGATGAGGCTGGAGCCACAGAGGGGTTTGATCAGTATGGATATGAT
GGAGAGGATTTCATAGCATTTGACCTGAAGACAAAGTCATGGATCGCCCCAACGCCACAG
GCAGTCATCACCAAACTCAAGTGGGACAGTAACACAGCTCAGAATGAGTACTGGAAAAAC
TACCTCACCCAGGAGTGCATTGAG
>AS7_UBA1_s3_481
ACAGTATCTGCTGCAACCAATACACTGCAGTATTTCTACACGGCCACTTCTGGTATAGAT
AACTTCCCAGAGTTTGTGACTATGGGAATCGTCAATGGCCATCAGATTGACCACTACGAC
AGTATCACCAAGAGAGCAATCCAGAAAGCTGAGTGGATCAGTGGGGCAGTTGACCCTGAC
TATTGGAAGACTAACACCCAGATCTATGCTGGTACAGAGACAGTTTTCGTGAACAACATT
AATGTTGCGAAATCCCGCTTCAATCAAACTGGAGGTGTGCACGTTTTCCAGATGATGTGT
GGTTGTGAGTGGGATGATGAGGCTGGAGCCACAGAGGGGTTTGATCAGTATGGATATGAT
GGAGAGGATTTCATAGCATTTGACCTGAAGACAAAGACATGGATCGCCCCAAAGACACAG
GCAGTCAACACCAAAAACAAGTGGGACAGTGACACAGCTCAGAATGAGTTCCTAAAAAAC
TACTACACCCAGACCTGCATTGAG
>AS7_UBA1_s4_289
ACAGCATCTGCTGTGACTCACGCCCTGAAGTATTTCTACACCGCATCTTCTGAAGTTCCC
AACTTCCCAGAGTTTGTGGCTGTGGGGGTGGTGGATGGTGTTCAGATGTTTCACTATGAC
AGCAACAGCCAGAGAGCGGTGCCCAAACAGGACTGGGTAAACAAGGCAGCAGACCCACAG
TACTGGGAGAGGAACACTGGGAATTGCAAGGGTTCCCAGCAGATTTTCAAAGCCAACATC
GATATTGTAAAGCAGCGTTTTAACCAAAGTGGAGGTGTGCACGTTTTCCAGAATATGTAT
GGATGTGAGTGGGATGATGAGGCTGGAGTCACAGAGGGGTTTGATCAGTATGGATATGAT
GGAGAGGATTTCATAGCATTTGACCTGAAGACAAAGTCATGGATCGCCCCAACGCCACAG
GCAGTCATCACCAAACTCAAGTGGGACAGTAACACAGCTCAGAATGAGTACTGGAAAAAC
TACCTCACCCAGGAGTGCATTGAG
>AS7_UBA1_s5_171
ACAGCATCTGCTGTGACTCACGCCCTGAAGTATTTCTACACCGCATCTTCTGAAGTTCCC
AACTTCCCAGAGTTTGTGGCTGTGGGGGTGGTGGATGGTGTTCAGATGTTTCACTATGAC
AGCAACAGCCAGAGAGCGGTGCCCAAACAGGACTGGGTAAACAAGGCAGCAGACCCACAG
TACTGGGAGAGGAACACTGGGAATTGCAAGGGTTCCCAGCAGATTTTCAAAGCCAACATC
GATATTGTAAGGCAGCGTTTTAACCAAAGTGGAGGTGTGCACGTTTTCCAGAATATGTAT
GGATGTGAGTGGGATGATGAGGCTGGAGTCACAGAGGGGTTTGATCAGTATGGATATGAT
GGAGAGGATTTCATAGCATTTGACCTGAAGACAAAGACATGGATCGCCCCAAAGACACAG
GCAGTCAACACCAAAAACAAGTGGGACAGTGACACAGCTCAGAATGAGTTCCTAAAAAAC
TACTACACCCAGACCTGCATTGAG
>AS8_UBA1_s1_30959
ACAGCATCTGCTGTGACTCACTCCCTGAAGTATTTCTACACCGCATCTTCTGAAGTTCCC
AACTTCCCAGAGTTTGTAGTTGTGTCAATGGTGGATGGTGTTCAGATGGTTCACTATGAC
AGCAACAGCCAGAGAGCGGTGCCAAAACAGGACTGGATGAACAAGGCAGCAGAAGCACTG
CCACAGTACTGGGATATCGAGACAGGGAAATTTTTGGGTTCCCATCAGAGTTTCAAAGCC
AACATCGATATTTGTAAGCAGCGTTTTAACCAAAGTGGAGGTGTGCACATTGTCCAGAAG
ATGTATGGATGTGAGTGGGATGATGAGACTGGAGTCACAGAGGGGTTTAATCAGTATGGA
TATGATGGAGAGGATTTCATAGCATTTGACCTGAAGACATTGAAATGGATCGCCCCAACG
CCACAGGCAGTCATCACCAAACTCAAGTGGGACAGTGACACAGCTCAGAATGAGTACCGA

AAAAACTACTACACCCAGACCTGCATTGAG
>AS8_UBA1_s2_24959
ACAGCATCTGCTGCCACCCACTCTCTGAAATACTTCTACACAGCAGTCTCAGGTGATATT
GACTTCCCAGAGTTCACCATCGTTGGTCTGGTCAACAATGGCCAGTTTGTGTACTATGAC
AGCAACATAAAGAGGATGGTCCCCAAGACTGAGTGGATGAAGCAGAGCGCAGGAGCAGAT
TACTGGGACACGGAGAGTGAGAAGCAAGTTGGTCAAAATCAAGGCTTCAAAAACAACATT
CAAGTGCTAAAGGATCGCTTCAACCAGTCCATGTCCACAGGTGTGCACGTTAACCAGTGG
ATGTATGGATGTGAGTGGGATGATGAGGCTGGAGTCACAGAGGGGTTTGAACAGTGGGGA
TATGATGGAGAGGACTTCATAGCATTTGACCTGAAGACAAAGTCATGGATCGCCCCAACG
CCACAGGCAGTCATCACCAAACTCAAGTGGGACAGTGACACAGCTCAGAATGAGCACCGA
AAAAACTACTACACCCAGATCTGCATTGAG
>AS8_UBA1_s3_2725
ACAGCATCTGCTGTGACTCACTCCCTGAAGTATTTCTACACCGCATCTTCTGAAGTTCCC
AACTTCCCAGAGTTTGTAGTTGTGTCAATGGTGGATGGTGTTCAGATGGTTCACTATGAC
AGCAACAGCCAGAGAGCGGTGCCAAAACAGGACTGGATGAACAAGGCAGCAGAAGCACTG
CCACAGTACTGGGATATCGAGACAGGGAAATTTTTGGGTTCCCATCAGAGTTTCAAAGCC
AACATCGATATTTGTAAGCAGCGTTTTAACCAAAGTGGAGGTGTGCACATTGTCCAGAAG
ATGTATGGATGTGAGTGGGATGATGAGACTGGAGTCACAGAGGGGTTTAATCAGTATGGA
TATGATGGAGAGGATTTCATAGCATTTGACCTGAAGACATTGAAATGGATCGCCCCAACG
CCACAGGCAGTCATCACCAAACTCAAGTGGGACAGTGACACAGCTCAGAATGAGCACCGA
AAAAACTACTACACCCAGATCTGCATTGAG
>AS8_UBA1_s4_2424
ACAGCATCTGCTGCCACCCACTCTCTGAAATACTTCTACACAGCAGTCTCAGGTGATATT
GACTTCCCAGAGTTCACCATCGTTGGTCTGGTCAACAATGGCCAGTTTGTGTACTATGAC
AGCAACATAAAGAGGATGGTCCCCAAGACTGAGTGGATGAAGCAGAGCGCAGGAGCAGAT
TACTGGGACACGGAGAGTGAGAAGCAAGTTGGTCAAAATCAAGGCTTCAAAAACAACATT
CAAGTGCTAAAGGATCGCTTCAACCAGTCCATGTCCACAGGTGTGCACGTTAACCAGTGG
ATGTATGGATGTGAGTGGGATGATGAGGCTGGAGTCACAGAGGGGTTTGAACAGTGGGGA
TATGATGGAGAGGACTTCATAGCATTTGACCTGAAGACAAAGTCATGGATCGCCCCAACG
CCACAGGCAGTCATCACCAAACTCAAGTGGGACAGTGACACAGCTCAGAATGAGTACCGA
AAAAACTACTACACCCAGACCTGCATTGAG
>AS8_UBA1_s5_1597
ACAGCATCTGCTGTGACTCACTCCCTGAAGTATTTCTACACCGCATCTTCTGAAGTTCCC
AACTTCCCAGAGTTTGTAGTTGTGTCAATGGTGGATGGTGTTCAGATGGTTCACTATGAC
AGCAACAGCCAGAGAGCGGTGCCAAAACAGGACTGGATGAACAAGGCAGCAGAAGCACTG
CCACAGTACTGGGATATCGAGACAGGGAAATTTTTGGGTTCCCATCAGAGTTTCAAAGCC
AACATCGATATTTGTAAGCAGCGTTTTAACCAAAGTGGAGGTGTGCACATTGTCCAGAAG
ATGTATGGATGTGAGTGGGATGATGAGACTGGAGTCACAGAGGGGTTTAATCAGTATGGA
TATGATGGAGAGGATTTCATAGCATTTGACCTGAAGACATTGAAATGGATCGCCCCAACG
CCACAGGCAGTCATCACCAAACTCAAGTGGGACAGTGACACAGCTCAGAATGAGTACCGA
AAAAACTACTACACCCAGATCTGCATTGAG
>AS9_UBA1_s1_20357
ACAGCATCTGCTGTGACTCACTCCCTGAAGTATTTCTACACCGCATCTTCTGAAGTTCCC
AACTTCCCAGAGTTTGTAGTTGTGTCAATGGTGGATGGTGTTCAGATGGTTCACTATGAC
AGCAACAGCCAGAGAGCGGTGCCAAAACAGGACTGGATGAACAAGGCAGCAGAAGCACTG
CCACAGTACTGGGATATCGAGACAGGGAAATTTTTGGGTTCCCATCAGAGTTTCAAAGCC
AACATCGATATTTGTAAGCAGCGTTTTAACCAAAGTGGAGGTGTGCACATTGTCCAGAAG
ATGTATGGATGTGAGTGGGATGATGAGACTGGAGTCACAGAGGGGTTTAATCAGTATGGA
TATGATGGAGAGGATTTCATAGCATTTGACCTGAAGACATTGAAATGGATCGCCCCAACG
CCACAGGCAGTCATCACCAAACTCAAGTGGGACAGTGACACAGCTCAGAATGAGTACCGA
AAAAACTACTACACCCAGACCTGCATTGAG
>AS9_UBA1_s2_14112
ACAGCATCTGCTGTGACTCACGCCCTGAAGTATTTCTACACCGCATCTTCTGAAGTTCCC
AACTTCCCAGAGTTTGTGGCTGTGGGGGTGGTGGATGGTGTTCAGATGGTTCACTATGAC
AGCAACAGCCAGAGAGCGGTGCCAAAACAGGACTGGATGAACAAGGCAGCAGAAGCACTG
CCACAGTACTGGGATATCGAGACAGGGAATCTTTTGGGTTCCCATCAGAGTTTCAAAGCC
AACATCGATATTTGTAAGCAGCGTTTTAACCAAAGTGGAGGTGTGCACGTTAACCAGTGG
ATGTATGGATGTGAGTGGGATGATGAGGCTGGAGTCACAGAGGGGTTTGAACAGTGGGGA
TATGATGGAGAGGACTTCATAGCATTTGACCTGAAGACAAAGTCATGGATCGCCCCAACG
CCACAGGCAGTCATCACCAAACTCAAGTGGGACAGTGACACAGCTCAGAATGAGCACCGA
AAAAACTACTACACCCAGATCTGCATTGAG

>AS9_UBA1_s3_3551
ACAGCATCTGCTGTGACTCACTCCCTGAAGTATTTCTACACCGCATCTTCTGAAGTTCCC
AACTTCCCAGAGTTTGTAGTTGTGTCAATGGTGGATGGTGTTCAGATGGTTCACTATGAC
AGCAACAGCCAGAGAGCGGTGCCAAAACAGGACTGGATGAACAAGGCAGCAGAAGCACTG
CCACAGTACTGGGATATCGAGACAGGGAATCTTTTGGGTTCCCATCAGAGTTTCAAAGCC
AACATCGATATTTGTAAGCAGCGTTTTAACCAAAGTGGAGGTGTGCACGTTAACCAGTGG
ATGTATGGATGTGAGTGGGATGATGAGGCTGGAGTCACAGAGGGGTTTGAACAGTGGGGA
TATGATGGAGAGGACTTCATAGCATTTGACCTGAAGACAAAGTCATGGATCGCCCCAACG
CCACAGGCAGTCATCACCAAACTCAAGTGGGACAGTGACACAGCTCAGAATGAGCACCGA
AAAAACTACTACACCCAGATCTGCATTGAG
>AS9_UBA1_s4_3146
ACAGCATCTGCTGTGACTCACGCCCTGAAGTATTTCTACACCGCATCTTCTGAAGTTCCC
AACTTCCCAGAGTTTGTGGCTGTGGGGGTGGTGGATGGTGTTCAGATGGTTCACTATGAC
AGCAACAGCCAGAGAGCGGTGCCAAAACAGGACTGGATGAACAAGGCAGCAGAAGCACTG
CCACAGTACTGGGATATCGAGACAGGGAAATTTTTGGGTTCCCATCAGAGTTTCAAAGCC
AACATCGATATTTGTAAGCAGCGTTTTAACCAAAGTGGAGGTGTGCACATTGTCCAGAAG
ATGTATGGATGTGAGTGGGATGATGAGACTGGAGTCACAGAGGGGTTTAATCAGTATGGA
TATGATGGAGAGGATTTCATAGCATTTGACCTGAAGACATTGAAATGGATCGCCCCAACG
CCACAGGCAGTCATCACCAAACTCAAGTGGGACAGTGACACAGCTCAGAATGAGTACCGA
AAAAACTACTACACCCAGACCTGCATTGAG
>AS9_UBA1_s5_2549
ACAGCATCTGCTGTGACTCACTCCCTGAAGTATTTCTACACCGCATCTTCTGAAGTTCCC
AACTTCCCAGAGTTTGTAGTTGTGTCAATGGTGGATGGTGTTCAGATGGTTCACTATGAC
AGCAACAGCCAGAGAGCGGTGCCAAAACAGGACTGGATGAACAAGGCAGCAGAAGCACTG
CCACAGTACTGGGATATCGAGACAGGGAAATTTTTGGGTTCCCATCAGAGTTTCAAAGCC
AACATCGATATTTGTAAGCAGCGTTTTAACCAAAGTGGAGGTGTGCACGTTAACCAGTGG
ATGTATGGATGTGAGTGGGATGATGAGGCTGGAGTCACAGAGGGGTTTGAACAGTGGGGA
TATGATGGAGAGGACTTCATAGCATTTGACCTGAAGACAAAGTCATGGATCGCCCCAACG
CCACAGGCAGTCATCACCAAACTCAAGTGGGACAGTGACACAGCTCAGAATGAGCACCGA
AAAAACTACTACACCCAGATCTGCATTGAG
>AS10_UBA1_s1_71332
ACAGCATCTGCTGTGACTCACGCCCTGAAGTATTTCTACACCGCATCTTCTGAAGTTCCC
AACTTCCCAGAGTTTGTAGTTGTGGGGGTGGTGGATGGTGTTCAGATGGTTCACTATGAC
AGCAACAGCCAGAGAGCGGTGCCCAAACAGGACTGGGTAAACAAGGCAGCAGACCCACAG
TACTGGGAGAGGAACACTGGGATTTTCAAGGGTTCCCAGCAGACTTTCAAAGCCAACATC
GATATTGCAAAGCAGCGTTTTAACCAAAGTGGAGGTGTGCACGTTAACCAGTGGATGTAT
GGATGTGAGTGGGATGATGAGGCTGGAGTCACAGAGGGGTTTGAACAGTGGGGATATGAT
GGAGAGGACTTCATAGCATTTGACCTGAAGACAAAGTCATGGATCGCCCCAACGCCACAG
GCAGTCATCACCAAACTCAAGTGGGACAGTGACACAGCTCAGAATGAGCACCGAAAAAAC
TACTACACCCAGATCTGCATTGAG
>AS10_UBA1_s2_448
ACAGCATCTGCTGTGACCCATTCCCTGCGCTACTTCTACACTGCCACCACAGGAATCCCA
GACTTTCCTGAGTTTGTGGATGTGGGGGTGGTGAACGGTAAGGTCATCAGCTACTATGAC
AGCATCATAAAGAGGAAGGTCCCCAAGCAGAGCTGGATGGAGGAGAATCTCAACCAGCAG
TACTGGAATCAAGGCACAGATCAATTGAAGGGCACAGAGCAATCCTTCAAAGCCAACATT
CAAGTAGCACAGACACGTTTCAATCAAACTGGAGGTGTCCACATTTTCCAGTACATGTAC
GGCTGTACGTGGGATGATGACAGTGGAGTCACTGATGGGCTTAGGCAGTATGGATATGAT
GGGGAGGACTTCCTTGTGTACGACATGAAGGCGTTCACTTGGATCGCTCCTAAACTGCAA
GCTGAGATCACTACGCGCAAGTGGAACAATGAGCCTGCTCAAATGGAATACTTAAAGAGC
TACATCACCCAGGAGTGCGTTGAG
>AS10_UBA1_s3_331
ACAGCATCTGCTGTGACTCACGCCCTGAAGTATTTCTACACCGCATCTTCTGAAGTTCCC
AACTTCCCAGAGTTTGTAGTTGTGGGGGTGGTGGATGGTGTTCAGATGGTTCACTATGAC
AGCAACAGCCAGAGAGCGGTGCCCAAACAGGACTGGGTAAACAAGGCAGCAGACCCACAG
TACTGGGAGAGGAACACTGGGATTTTCAAGGGTTCCCAGCAGACTTTCAAAGCCAACATC
GATATTGCAAGGCAGCGTTTTAACCAAAGTGGAGGTGTGCACGTTAACCAGTGGATGTAT
GGATGTGAGTGGGATGATGAGGCTGGAGTCACAGAGGGGTTTGAACAGTGGGGATATGAT
GGAGAGGACTTCATAGCATTTGACCTGAAGACAAAGTCATGGATCGCCCCAACGCCACAG
GCAGTCATCACCAAACTCAAGTGGGACAGTGACACAGCTCAGAATGAGCACCGAAAAAAC
TACTACACCCAGATCTGCATTGAG
>AS10_UBA1_s4_325

ACAGCATCTGCTGTGACTCACGCCCTGAAGTATTTCTACACCGCATCTTCTGAAGTTCCC
AACTTCCCAGAGTTTGTAGTTGTGGGGGTGGTGGATGGTGCTCAGATGGTTCACTATGAC
AGCAACAGCCAGAGAGCGGTGCCCAAACAGGACTGGGTAAACAAGGCAGCAGACCCACAG
TACTGGGAGAGGAACACTGGGATTTTCAAGGGTTCCCAGCAGACTTTCAAAGCCAACATC
GATATTGCAAAGCAGCGTTTTAACCAAAGTGGAGGTGTGCACGTTAACCAGTGGATGTAT
GGATGTGAGTGGGATGATGAGGCTGGAGTCACAGAGGGGTTTGAACAGTGGGGATATGAT
GGAGAGGACTTCATAGCATTTGACCTGAAGACAAAGTCATGGATCGCCCCAACGCCACAG
GCAGTCATCACCAAACTCAAGTGGGACAGTGACACAGCTCAGAATGAGCACCGAAAAAAC
TACTACACCCAGATCTGCATTGAG
>AS10_UBA1_s5_322
ACAGCATCTGCTGTGACTCACGCCCTGAAGTATTTCTACACCGCATCTTCTGAAGTTCCC
AACTTCCCAGAGTTTGTAGTTGTGGGGGTGGTGGATGGTGTTCAGATGGTTCACTATGAC
AGCAACAGCCAGAGAGCGGTGCCCAAACAGGACTGGGTAAACAAGGCAGCAGACCCACAG
TACTGGGAGAGGAACACTGGGATTTTCAAGGGTTCCCAGCAGACTTTCAAAGCCAACATC
GATATTGCAAAGCAGCGTTTTAACCAAAGTGGAGGTGTGCACGTTAACCAGGGGATGTAT
GGATGTGAGTGGGATGATGAGGCTGGAGTCACAGAGGGGTTTGAACAGTGGGGATATGAT
GGAGAGGACTTCATAGCATTTGACCTGAAGACAAAGTCATGGATCGCCCCAACGCCACAG
GCAGTCATCACCAAACTCAAGTGGGACAGTGACACAGCTCAGAATGAGCACCGAAAAAAC
TACTACACCCAGATCTGCATTGAG

Supplementary file 3B. Deduced MHC class I and class II amino acid sequences identified in this study

Deduced amino acid sequences for the MHC sequences identified in this study using the custom library preparation and Illumina MiSeq sequencing. The top five sequences are shown for each gene in each animal.

Deduced MHC class II alpha (DAA) sequences:

>AS1_DAA_s1_39861
HKVLHIDLVISGCSDSDGVDMYGLDGEEMWYADFNKGEGVMPLPPFADPFTYPGAYEQAV
GNQGVCKANLAVNIKAYKNPEEKIAPPHSSIYPRDDVDLGVENTLICHVSGFYPAPVRVR
WTRNNQNLTEGVRLSTP
>AS1_DAA_s2_37247
HKVLHIDLHIIGCSDSDGVDMYGLDGEEMWYADFNKGEGVVALPPFADPFTFPGFYEGAV
GNQGVCKANLAVNIKAYKNPEEKIDPPHSSIYPRDDVDLGVENTLICHVSGFFPAPVRVR
WTRNNQNLTEGVRLSTP
>AS1_DAA_s3_2785
HKVLHIDLHIIGCSDSDGVDMYGLDGEEMWYADFNKGEGVVALPPFADPFTFPGFYEGAV
GNQGVCKANLAVNIKAYKNPEEKIDPPHSSIYPRDDVDLGVENTLICHVSGFYPAPVRVR
WTRNNQNLTEGVRLSTP
>AS1_DAA_s4_2703
HKVLHIDLVISGCSDSDGVDMYGLDGEEMWYADFNKGEGVMPLPPFADPFTYPGAYEQAV
GNQGVCKANLAVNIKAYKNPEEKIDPPHSSIYPRDDVDLGVENTLICHVSGFFPAPVRVR
WTRNNQNLTEGVRLSTP
>AS1_DAA_s5_2591
HKVLHIDLHIIGCSDSDGVDMYGLDGEEMWYADFNKGEGVMPLPPFADPFTYPGAYEQAV
GNQGVCKANLAVNIKAYKNPEEKIAPPHSSIYPRDDVDLGVENTLICHVSGFYPAPVRVR
WTRNNQNLTEGVRLSTP
>AS2_DAA_s1_29496
HKVLHIDLVISGCSDSDGVDMYGLDGEEMWYADFNKGEGVMPLPPFADPFTYPGAYEQAV
GNQGVCKANLAVNIKAYKNPEEKIAPPHSSIYPRDDVDLGVENTLICHVSGFYPAPVRVR
WTRNNQNLTEGVRLSTP
>AS2_DAA_s2_28281
HKVLHIDLAITGCSDSDGLDMYGLDGEEMWYADFNKGEGVMPLPPFADPFTYPGAYEGAV
GNQGICKANLATCIKAYKNPEEKIAPPHSSIYPRDDVDLGVENTLICHVSGFHPAPVRVR
WTRNNQNLTEGVRLSTP
>AS2_DAA_s3_4098
HKVLHIDLVISGCSDSDGVDMYGLDGEEMWYADFNKGEGVMPLPPFADPFTYPGAYEQAV
GNQGVCKANLAVNIKAYKNPEEKIAPPHSSIYPRDDVDLGVENTLICHVSGFHPAPVRVR
WTRNNQNLTEGVRLSTP

>AS2_DAA_s4_4026
HKVLHIDLAITGCSDSDGLDMYGLDGEEMWYADFNKGEGVMPLPPFADPFTYPGAYEGAV
GNQGICKANLATCIKAYKNPEEKIAPPHSSIYPRDDVDLGVENTLICHVSGFYPAPVRVR
WTRNNQNLTEGVRLSTP
>AS2_DAA_s5_3783
HKVLHIDLVISGCSDSDGVDMYGLDGEEMWYADFNKGEGVMPLPPFADPFTYPGAYEGAV
GNQGICKANLATCIKAYKNPEEKIAPPHSSIYPRDDVDLGVENTLICHVSGFHPAPVRVR
WTRNNQNLTEGVRLSTP
>AS3_DAA_s1_14569
HKVLHIDLVITGCSDSDGLDMYGLDGEEMWYADFNKQEGVVALPPFADPFTFPGFYEQAV
GNQGVCKGNLAKCIKAYKNPEEKIDPPHSSIYPRDDVDLGVENTLICHVSGFFPAPVRVR
WTRNNQNLTEGVRLSTP
>AS3_DAA_s2_12123
HKVLHIDLVITGCSDSDGVDMYGLDGEEMWYADFNKGEGVVALPPFADPFTFHGAYEGAV
GNQGVCKANLAVNIKAYKNPEEKIDPPHSSIYPRDDVDLGVENTLICHVSGFHPAPVRVR
WTRNNQNLTEGVRLSTP
>AS3_DAA_s3_2271
HKVLHIDLVITGCSDSDGLDMYGLDGEEMWYADFNKQEGVVALPPFADPFTFPGFYEQAV
GNQGVCKGNLAKCIKAYKNPEEKIDPPHSSIYPRDDVDLGVENTLICHVSGFHPAPVRVR
WTRNNQNLTEGVRLSTP
>AS3_DAA_s4_2119
HKVLHIDLVITGCSDSDGVDMYGLDGEEMWYADFNKGEGVVALPPFADPFTFHGAYEGAV
GNQGVCKANLAVNIKAYKNPEEKIDPPHSSIYPRDDVDLGVENTLICHVSGFFPAPVRVR
WTRNNQNLTEGVRLSTP
>AS3_DAA_s5_888
HKVLHIDLVITGCSDSDGLDMYGLDGEEMWYADFNKGEGVVALPPFADPFTFHGAYEGAV
GNQGVCKANLAVNIKAYKNPEEKIDPPHSSIYPRDDVDLGVENTLICHVSGFHPAPVRVR
WTRNNQNLTEGVRLSTP
>AS5_DAA_s1_38290
HKVLHIDLVISGCSDSDGVDMYGLDGEEMWYADFNKGEGVMPLPPFADPFTYPGAYEQAV
GNQGVCKANLAVNIKAYKNPEEKIAPPHSSIYPRDDVDLGVENTLICHVSGFYPAPVRVR
WTRNNQNLTEGVRLSTP
>AS5_DAA_s2_181
HKVLHIDLVISGCSDSDGVDMYGLDGEGMWYADFNKGEGVMPLPPFADPFTYPGAYEQAV
GNQGVCKANLAVNIKAYKNPEEKIAPPHSSIYPRDDVDLGVENTLICHVSGFYPAPVRVR
WTRNNQNLTEGVRLSTP
>AS5_DAA_s3_176
HKVQHIDLVISGCSDSDGVDMYGLDGEEMWYADFNKGEGVMPLPPFADPFTYPGAYEQAV
GNQGVCKANLAVNIKAYKNPEEKIAPPHSSIYPRDDVDLGVENTLICHVSGFYPAPVRVR
WTRNNQNLTEGVRLSTP
>AS5_DAA_s4_172
HKVLHIDLVISGCSDSDGVDMYGLDGEEMWYADFNKGEGVMPLPPFADPFTYPGAYEQAV
GNQGVCKANLAVNIKAYKNPEEKIAPPHSSIYPRDDVDLGVENTLICHVSGFYPAPVRVR
WTRNNQNLTEGVRLSTP
>AS5_DAA_s5_162
HKVLHIDLVISGCSDSDGVDMYGLDGEEMWYADFNKGEGVMPLPPFADPFTYPGAYEQAV
GNQGVCKANLAVNIKAYKNPEGKIAPPHSSIYPRDDVDLGVENTLICHVSGFYPAPVRVR
WTRNNQNLTEGVRLSTP
>AS6_DAA_s1_21935
HKVLHIDLYISGCSDSDGLDMYGLDGEEMWYADFNKGEGVVALPPFADPFTFPGFYEGAV
GNQGVCKANLAVNIKAYKNPEEKIDPPHSSIYPRDDVDLGVENTLICHVSGFHPAPVRVR
WTRNNQNLTEGVRLSTP
>AS6_DAA_s2_19807
HKVLHIDLVITGCSDSDGVDMYGLDGEEMWYADFNKGEGVVALPPFADPFTFHGAYEGAV
GNQGVCKANLAVNIKAYKNPEEKIDPPHSSIYPRDDVDLGVENTLICHVSGFHPAPVRVR
WTRNNQNLTEGVRLSTP
>AS6_DAA_s3_2360
HKVLHIDLVITGCSDSDGVDMYGLDGEEMWYADFNKGEGVVALPPFADPFTFPGFYEGAV
GNQGVCKANLAVNIKAYKNPEEKIDPPHSSIYPRDDVDLGVENTLICHVSGFHPAPVRVR
WTRNNQNLTEGVRLSTP
>AS6_DAA_s4_2123

HKVLHIDLYISGCSDSDGLDMYGLDGEEMWYADFNKGEGVVALPPFADPFTFHGAYEGAV
GNQGVCKANLAVNIKAYKNPEEKIDPPHSSIYPRDDVDLGVENTLICHVSGFHPAPVRVR
WTRNNQNLTEGVRLSTP
>AS6_DAA_s5_393
HKVLHIDLYISGCSDSDGVDMYGLDGEEMWYADFNKGEGVVALPPFADPFTFHGAYEGAV
GNQGVCKANLAVNIKAYKNPEEKIDPPHSSIYPRDDVDLGVENTLICHVSGFHPAPVRVR
WTRNNQNLTEGVRLSTP
>AS7_DAA_s1_31665
HKVLHIDLVISGCSDSDGVDMYGLDGEEMWYADFNKGEGVMPLPPFADPFTYPGAYEQAV
GNQGVCKANLAVNIKAYKNPEEKIAPPHSSIYPRDDVDLGVENTLICHVSGFYPAPVRVR
WTRNNQNLTEGVRLSTP
>AS7_DAA_s2_29058
HKVLHIDLYISGCSDSDGLDMYGLDGEEMWYADFNKGEGVVALPPFADPFTFPGFYEGAV
GNQGVCKANLAVNIKAYKNPEEKIDPPHSSIYPRDDVDLGVENTLICHVSGFHPAPVRVR
WTRNNQNLTEGVRLSTP
>AS7_DAA_s3_2271
HKVLHIDLVISGCSDSDGVDMYGLDGEEMWYADFNKGEGVMPLPPFADPFTYPGAYEQAV
GNQGVCKANLAVNIKAYKNPEEKIDPPHSSIYPRDDVDLGVENTLICHVSGFHPAPVRVR
WTRNNQNLTEGVRLSTP
>AS7_DAA_s4_2177
HKVLHIDLVISGCSDSDGVDMYGLDGEEMWYADFNKGEGVMPLPPFADPFTYPGAYEQAV
GNQGVCKANLAVNIKAYKNPEEKIAPPHSSIYPRDDVDLGVENTLICHVSGFHPAPVRVR
WTRNNQNLTEGVRLSTP
>AS7_DAA_s5_2085
HKVLHIDLYISGCSDSDGLDMYGLDGEEMWYADFNKGEGVVALPPFADPFTFPGFYEGAV
GNQGVCKANLAVNIKAYKNPEEKIDPPHSSIYPRDDVDLGVENTLICHVSGFYPAPVRVR
WTRNNQNLTEGVRLSTP
>AS8_DAA_s1_25961
HKVLHIDLHIIGCSDSDGVDMYGLDGEEMWYADFNKGEGVVALPPFADPFTFPGFYEGAV
GNQGVCKANLAVNIKAYKNPEEKIDPPHSSIYPRDDVDLGVENTLICHVSGFFPAPVRVR
WTRNNQNLTEGVRLSTP
>AS8_DAA_s2_22389
HKVLHIDLVITGCSDSDGLDMYGLDGEEMWYADFNKQEGVVALPPFADPFTFPGFYEQAV
GNQGVCKGNLAKCIKAYKNPEEKIDPPHSSIYPRDDVDLGVENTLICHVSGFFPAPVRVR
WTRNNQNLTEGVRLSTP
>AS8_DAA_s3_1672
HKVLHIDLHIIGCSDSDGVDMYGLDGEEMWYADFNKGEGVVALPPFADPFTFPGFYEQAV
GNQGVCKGNLAKCIKAYKNPEEKIDPPHSSIYPRDDVDLGVENTLICHVSGFFPAPVRVR
WTRNNQNLTEGVRLSTP
>AS8_DAA_s4_1497
HKVLHIDLVITGCSDSDGLDMYGLDGEEMWYADFNKQEGVVALPPFADPFTFPGFYEGAV
GNQGVCKANLAVNIKAYKNPEEKIDPPHSSIYPRDDVDLGVENTLICHVSGFFPAPVRVR
WTRNNQNLTEGVRLSTP
>AS8_DAA_s5_876
HKVLHIDLVITGCSDSDGLDMYGLDGEEMWYADFNKGEGVVALPPFADPFTFPGFYEGAV
GNQGVCKANLAVNIKAYKNPEEKIDPPHSSIYPRDDVDLGVENTLICHVSGFFPAPVRVR
WTRNNQNLTEGVRLSTP
>AS9_DAA_s1_27650
HKVLHIDLAITGCSDSDGLDMYGLDGEEMWYADFNKGEGVMPLPPFADPFTYPGAYEGAV
GNQGICKANLATCIKAYKNPEEKIAPPHSSIYPRDDVDLGVENTLICHVSGFHPAPVRVR
WTRNNQNLTEGVRLSTP
>AS9_DAA_s2_22549
HKVLHIDLVITGCSDSDGVDMYGLDGEEMWYADFNKGEGVVALPPFADPFTFHGAYEGAV
GNQGVCKANLAVNIKAYKNPEEKIDPPHSSIYPRDDVDLGVENTLICHVSGFHPAPVRVR
WTRNNQNLTEGVRLSTP
>AS9_DAA_s3_2292
HKVLHIDLAITGCSDSDGLDMYGLDGEEMWYADFNKGEGVVALPPFADPFTFHGAYEGAV
GNQGVCKANLAVNIKAYKNPEEKIDPPHSSIYPRDDVDLGVENTLICHVSGFHPAPVRVR
WTRNNQNLTEGVRLSTP
>AS9_DAA_s4_2277
HKVLHIDLVITGCSDSDGVDMYGLDGEEMWYADFNKGEGVMPLPPFADPFTYPGAYEGAV

GNQGICKANLATCIKAYKNPEEKIAPPHSSIYPRDDVDLGVENTLICHVSGFHPAPVRVR
WTRNNQNLTEGVRLSTP
>AS9_DAA_s5_1054
HKVLHIDLAITGCSDSDGLDMYGLDGEEMWYADFNKGEGVMPLPPFADPFTYPGAYEGAV
GNQGICKANLATCIKAYKNPEEKIDPPHSSIYPRDDVDLGVENTLICHVSGFHPAPVRVR
WTRNNQNLTEGVRLSTP
>AS10_DAA_s1_90816
HKVLHIDLHIIGCSDSDGVDMYGLDGEEMWYADFNKGEGVVALPPFADPFTFPGFYEGAV
GNQGVCKANLAVNIKAYKNPEEKIDPPHSSIYPRDDVDLGVENTLICHVSGFFPAPVRVR
WTRNNQNLTEGVRLSTP
>AS10_DAA_s2_422
HKVPHIDLHIIGCSDSDGVDMYGLDGEEMWYADFNKGEGVVALPPFADPFTFPGFYEGAV
GNQGVCKANLAVNIKAYKNPEEKIDPPHSSIYPRDDVDLGVENTLICHVSGFFPAPVRVR
WTRNNQNLTEGVRLSTP
>AS10_DAA_s3_419
HKVLHIDLHIIGCSDSDGVDMYGLDGEEMWYADFNKGEGVVALPPFADPFTFPGFYEGAV
GNQGVCKANLAVNIKAYKNPEEKIDPPHSSIYPRDDVDLGVENTLICHVSGFFPAPVRVR
WTRNNQNLTEGVRLSTP
>AS10_DAA_s4_410
HKVLHIDLHIIGCSDSDGVDMYGLDGEEMWYADFNKGGGVVALPPFADPFTFPGFYEGAV
GNQGVCKANLAVNIKAYKNPEEKIDPPHSSIYPRDDVDLGVENTLICHVSGFFPAPVRVR
WTRNNQNLTEGVRLSTP
>AS10_DAA_s5_407
HKVLHIDLHIIGCSDSDGVDMYGLDGEEMWYADFNKGEGVVALPPFADPFTFPGFYEGAV
GNQGVCKANLAVNIKAYKNPEEKIDPPHSSIYPRDDVDLGVENTLICHVSGFFPAPVRVR
WTRNNQNLTEGVRLSTP
>AS1_DAB2_s1_63791
VLSIFSGTDGYFEQVVRQCRYSSKDLQGIEFIDSYVFNKAEYVRFNSTVGKYVGYTELGV
KNAEAWNKGPELAVELGELERYCKLNAPIDYSAILDKTVEPHVRLSSVAPPSGRHPAMLM
CSAYDFYPKPIRVTWLRDGREVKSDVTSTEE

Deduced MHC class II beta (DAB) sequences:

>AS1_DAB2_s2_20621
VLSIFSGTDGYFYHMMTQCRYSSKDLQGIELITSYVFNQAENIRFNSTVGKFVGYTEHGV
KNAEAWNKGPELAGELGVLERYCKFNAPIDYSAILDKTVEPHVRLSSVAPPSGRHPAMLM
CSAYDFFPKPIRVTWLRDGREVKSDVTSTEE
>AS1_DAB2_s3_5382
VLSIFSGTDGYFYHMMTQCRYSSKDLQGIELITSYVFNQAENIRFNSTVGKFVGYTEHGV
KNAEAWNKGPELAGELGVLERYCKFNAPIDYSAILDKTVEPHVRLSSVAPPSGRHPAMLM
CSAYDFYPKPIRVTWLRDGREVKSDVTSTEE
>AS1_DAB2_s4_4227
VLSIFSGTDGYFEQVVRQCRYSSKDLQGIEFIDSYVFNKAEYVRFNSTVGKYVGYTELGV
KNAEAWNKGPELAVELGELERYCKLNAPIDYSAILDKTVEPHVRLSSVAPPSGRHPAMLM
CSAYDFFPKPIRVTWLRDGREVKSDVTSTEE
>AS1_DAB2_s5_3463
VLSIFSGTDGYFEQVVRQCRYSSKDLQGIEFIDSYVFNKAEYVRFNSTVGKYVGYTELGV
KNAEAWNKGPELAVELGELERFCKHNAAIYYSAILDKTVEPHVRLSSVAPPSGRHPAMLM
CSAYDFYPKPIRVTWLRDGREVKSDVTSTEE
>AS2_DAB1_s1_30852
VSLTLVLSIFSGTDGYFFQVVRQCRYSSKDLQGIEFIHSYVFNQAENIRFNSTVGKYVGY
TELGVKNAEAWNKGPELAVELGELERYCKHNADIDYSAILDKTVEPHVRLSSVAPPSGRH
PAMLMCSAYDFYPKPIRVTWLRDGREVKSDVTSTEE
>AS2_DAB1_s2_17876
VSLTLVLSIFSGTDGYFYHMMTQCRYSSKDLQGIELITSYVFNQAENIRFNSTVGKFVGY
TEHGVKNAEAWNKGPELAGELGVLERYCKFNAPIDYSAILDKTVEPHVRLSSVAPPSGRH
PAMLMCSAYDFFPKPIRVTWLRDGREVKSDVTSTEE
>AS2_DAB1_s3_3147
VSLTLVLSIFSGTDGYFYHMMTQCRYSSKDLQGIELITSYVFNQAENIRFNSTVGKFVGY
TEHGVKNAEAWNKGPELAGELGVLERYCKFNAPIDYSAILDKTVEPHVRLSSVAPPSGRH
PAMLMCSAYDFYPKPIRVTWLRDGREVKSDVTSTEE

```
>AS2_DAB1_s4_2822
VSLTLVLSIFSGTDGYFFQVVRQCRYSSKDLQGIEFIHSYVFNQAENIRFNSTVGKYVGY
TELGVKNAEAWNKGPELAVELGELERYCKHNADIDYSAILDKTVEPHVRLSSVAPPSGRH
PAMLMCSAYDFFPKPIRVTWLRDGREVKSDVTSTEE
>AS2_DAB1_s5_1143
VSLTLVLSIFSGTDGYFYHMMTQCRYSSKDLQGIELITSYVFNQAENIRFNSTVGKFVGY
TEHGVKNAEAWNKGPELAVELGELERYCKHNADIDYSAILDKTVEPHVRLSSVAPPSGRH
PAMLMCSAYDFYPKPIRVTWLRDGREVKSDVTSTEE
>AS3_DAB1_s1_10179
VSLTLVLSIFSGTDGYFEQVVRQCRYSSKDLQGIEFIDSYVFNKAEYVRFNSTVGKYVGY
TELGVKNAEAWNKGPELAVELGELERFCKHNADLHYRAILDKTVEPHVRLSSVAPPSGRH
PAMLMCSAYDFYPKPIRVTWLRDGREVKSDVTSTEE
>AS3_DAB1_s2_7127
VSLTLVLSIFSGTDGYFYQRVSECRYSSKDLQGIEFIDSYVFNKAEYVRFNSTVGKYVGY
TEYGVKNAEAWNKGPELAGELGELERVCKHNAPIDYSAILDKTVEPHVRLSSVAPPSGRH
PAMLMCSAYDFFPKPIRVTWLRDGREVKSDVTSTEE
>AS3_DAB1_s3_1585
VSLTLVLSIFSGTDGYFYQRVSECRYSSKDLQGIEFIDSYVFNKAEYVRFNSTVGKYVGY
TEYGVKNAEAWNKGPELAGELGELERVCKHNAPIDYSAILDKTVEPHVRLSSVAPPSGRH
PAMLMCSAYDFYPKPIRVTWLRDGREVKSDVTSTEE
>AS3_DAB1_s4_1553
VSLTLVLSIFSGTDGYFEQVVRQCRYSSKDLQGIEFIDSYVFNKAEYVRFNSTVGKYVGY
TEYGVKNAEAWNKGPELAGELGELERVCKHNAPIDYSAILDKTVEPHVRLSSVAPPSGRH
PAMLMCSAYDFFPKPIRVTWLRDGREVKSDVTSTEE
>AS3_DAB1_s5_1509
VSLTLVLSIFSGTDGYFYQRVSECRYSSKDLQGIEFIDSYVFNKAEYVRFNSTVGKYVGY
TELGVKNAEAWNKGPELAVELGELERFCKHNADLHYRAILDKTVEPHVRLSSVAPPSGRH
PAMLMCSAYDFYPKPIRVTWLRDGREVKSDVTSTEE
>AS5_DAB2_s1_11545
VLSIFSGTDGYFYHMMTQCRYSSKDLQGIELITSYVFNQAENIRFNSTVGKFVGYTEHGV
KNAEAWNKGPELAGELGVLERYCKFNAPIDYSAILDKTVEPHVRLSSVAPPSGRHPAMLM
CSAYDFFPKPIRVTWLRDGREVKSDVTSTEE
>AS5_DAB2_s2_9933
VLSIFSGTDGYFYHMMRQCRYSSKDLQGIELITSYVFNQAENIRFNSTVGKFVGYTEHGV
KNAEAWNKGPELAGELGELERVCKHNAPIYYSAILDKTVEPHVRLSSVAPPSGRHPAMLM
CSAYDFFPKPIRVTWLRDGREVKSDVTSTEE
>AS5_DAB2_s3_1500
VLSIFSGTDGYFYHMMRQCRYSSKDLQGIELITSYVFNQAENIRFNSTVGKFVGYTEHGV
KNAEAWNKGPELAGELGVLERYCKFNAPIDYSAILDKTVEPHVRLSSVAPPSGRHPAMLM
CSAYDFFPKPIRVTWLRDGREVKSDVTSTEE
>AS5_DAB2_s4_1433
VLSIFSGTDGYFYHMMTQCRYSSKDLQGIELITSYVFNQAENIRFNSTVGKFVGYTEHGV
KNAEAWNKGPELAGELGELERVCKHNAPIYYSAILDKTVEPHVRLSSVAPPSGRHPAMLM
CSAYDFFPKPIRVTWLRDGREVKSDVTSTEE
>AS5_DAB2_s5_994
VLSIFSGTDGYFEQVVRQCRYSSKDLQGIEFIDSYVFNKAEYVRFNSTVGKYVGYTELGV
KNAEAWNKGPELAVELGELERFCKHNAAIYYSAILDKTVEPHVRLSSVAPPSGRHPAMLM
CSAYDFYPKPIRVTWLRDGREVKSDVTSTEE
>AS6_DAB1_s1_10396
VSLTLVLSIFSGTDGYFEQVVRQCRYSSKDLQGIEFIDSYVFNKAEYVRFNSTVGKYVGY
TELGVKNAEAWNKGPELAVELGELERYCKLNAPIDYSAILDKTVEPHVRLSSVAPPSGRH
PAMLMCSAYDFYPKPIRVTWLRDGREVKSDVTSTEE
>AS6_DAB1_s2_9420
VSLTLVLSIFSGTDGYFEQVVRQCRYSSKDLQGIEFIDSYVFNKAEYVRFNSTVGKYVGY
TELGVKNAEAWNKGPELAVELGELERFCKHNAAIYYSAILDKTVEPHVRLSSVAPPSGRH
PAMLMCSAYDFYPKPIRVTWLRDGREVKSDVTSTEE
>AS6_DAB1_s3_1464
VSLTLVLSIFSGTDGYFEQVVRQCRYSSKDLQGIEFIDSYVFNKAEYVRFNSTVGKYVGY
TELGVKNAEAWNKGPELAVELGELERYCKLNAPIDYSAILDKTVEPHVRLSSVAPPSGRH
PAMLMCSAYDFYPKPIRVTWLRDGREVKSDVTSTEE
>AS6_DAB1_s4_1282
```

VSLTLVLSIFSGTDGYFEQVVRQCRYSSKDLQGIEFIDSYVFNKAEYVRFNSTVGKYVGY
TELGVKNAEAWNKGPELAVELGELERFCKHNAAIYYSAILDKTVEPHVRLSSVAPPSGRH
PAMLMCSAYDFYPKPIRVTWLRDGREVKSDVTSTEE
>AS6_DAB1_s5_82
VSLTLVLSIFSGTDGYFEQVVRQCRYSSKDLQGIEFIDSYVFNKAEYVRFNSTVGKYVGY
TELGVKNAEAWNKGPELAVELGELERFCKHNAAIYYRAILDKTVEPHVRLSSVAPPSGRH
PAMLMCSAYDFYPKPIRVTWLRDGREVKSDVTSTEE
>AS7_DAB2_s1_71872
VLSIFSGTDGYFEQVVRQCRYSSKDLQGIEFIDSYVFNKAEYVRFNSTVGKYVGYTELGV
KNAEAWNKGPELAVELGELERFCKHNAAIYYSAILDKTVEPHVRLSSVAPPSGRHPAMLM
CSAYDFYPKPIRVTWLRDGREVKSDVTSTEE
>AS7_DAB2_s2_24758
VLSIFSGTDGYFYHMMTQCRYSSKDLQGIELITSYVFNQAENIRFNSTVGKFVGYTEHGV
KNAEAWNKGPELAGELGVLERYCKFNAPIDYSAILDKTVEPHVRLSSVAPPSGRHPAMLM
CSAYDFFPKPIRVTWLRDGREVKSDVTSTEE
>AS7_DAB2_s3_7352
VLSIFSGTDGYFYHMMTQCRYSSKDLQGIELITSYVFNQAENIRFNSTVGKFVGYTEHGV
KNAEAWNKGPELAGELGVLERYCKFNAPIDYSAILDKTVEPHVRLSSVAPPSGRHPAMLM
CSAYDFYPKPIRVTWLRDGREVKSDVTSTEE
>AS7_DAB2_s4_5908
VLSIFSGTDGYFEQVVRQCRYSSKDLQGIEFIDSYVFNKAEYVRFNSTVGKYVGYTELGV
KNAEAWNKGPELAVELGELERFCKHNAAIYYSAILDKTVEPHVRLSSVAPPSGRHPAMLM
CSAYDFFPKPIRVTWLRDGREVKSDVTSTEE
>AS7_DAB2_s5_1869
VLSIFSGTDGYFEQVVRQCRYSSKDLQGIEFIDSYVFNKAEYVRFNSTVGKYVGYTELGV
KNAEAWNKGPELAGELGVLERYCKFNAPIDYSAILDKTVEPHVRLSSVAPPSGRHPAMLM
CSAYDFFPKPIRVTWLRDGREVKSDVTSTEE
>AS8_DAB2_s1_41329
VLSIFSGTDGYFEQVVRQCRYSSKDLQGIEFIDSYVFNKAEYVRFNSTVGKFVGYTELGV
KNAEAWNKGPELAVELGELERYCKLNAPIDYSAILDKTVEPHVRLSSVAPPSGRHPAMLM
CSAYDFYPKPIRVTWLRDGREVKSDVTSTEE
>AS8_DAB2_s2_29683
VLSIFSGTDGYFYQRVSECRYSSKDLQGIEFIDSYVFNKAEYVRFNSTVGKYVGYTEYGV
KNAEAWNKGPELAGELGELERVCKHNAPIDYSAILDKTVEPHVRLSSVAPPSGRHPAMLM
CSAYDFFPKPIRVTWLRDGREVKSDVTSTEE
>AS8_DAB2_s3_7380
VLSIFSGTDGYFEQVVRQCRYSSKDLQGIEFIDSYVFNKAEYVRFNSTVGKFVGYTELGV
KNAEAWNKGPELAVELGELERYCKLNAPIDYSAILDKTVEPHVRLSSVAPPSGRHPAMLM
CSAYDFFPKPIRVTWLRDGREVKSDVTSTEE
>AS8_DAB2_s4_7176
VLSIFSGTDGYFYQRVSECRYSSKDLQGIEFIDSYVFNKAEYVRFNSTVGKYVGYTEYGV
KNAEAWNKGPELAGELGELERVCKHNAPIDYSAILDKTVEPHVRLSSVAPPSGRHPAMLM
CSAYDFYPKPIRVTWLRDGREVKSDVTSTEE
>AS8_DAB2_s5_5682
VLSIFSGTDGYFYQRVSECRYSSKDLQGIEFIDSYVFNKAEYVRFNSTVGKFVGYTELGV
KNAEAWNKGPELAVELGELERYCKLNAPIDYSAILDKTVEPHVRLSSVAPPSGRHPAMLM
CSAYDFYPKPIRVTWLRDGREVKSDVTSTEE
>AS9_DAB1_s1_16301
VSLTLVLSIFSGTDGYFEQVVRQCRYSSKDLQGIEFIDSYVFNKAEYVRFNSTVGKYVGY
TELGVKNAEAWNKGPELAVELGELERFCKHNADLHYRAILDKTVEPHVRLSSVAPPSGRH
PAMLMCSAYDFYPKPIRVTWLRDGREVKSDVTSTEE
>AS9_DAB1_s2_10525
VSLTLVLSIFSGTDGYFFQVVRQCRYSSKDLQGIEFIHSYVFNQAENIRFNSTVGKYVGY
TELGVKNAEAWNKGPELAVELGELERYCKHNADIDYSAILDKTVEPHVRLSSVAPPSGRH
PAMLMCSAYDFYPKPIRVTWLRDGREVKSDVTSTEE
>AS9_DAB1_s3_2669
VSLTLVLSIFSGTDGYFEQVVRQCRYSSKDLQGIEFIDSYVFNKAEYVRFNSTVGKYVGY
TELGVKNAEAWNKGPELAVELGELERFCKHNADLHYRAILDKTVEPHVRLSSVAPPSGRH
PAMLMCSAYDFYPKPIRVTWLRDGREVKSDVTSTEE
>AS9_DAB1_s4_2356
VSLTLVLSIFSGTDGYFFQVVRQCRYSSKDLQGIEFIHSYVFNQAENIRFNSTVGKYVGY

```
TELGVKNAEAWNKGPELAVELGELERYCKHNADIDYSAILDKTVEPHVRLSSVAPPSGRH
PAMLMCSAYDFYPKPIRVTWLRDGREVKSDVTSTEE
>AS9_DAB1_s5_2051
VSLTLVLSIFSGTDGYFEQVVRQCRYSSKDLQGIEFIDSYVFNKAEYVRFNSTVGKYVGY
TELGVKNAEAWNKGPELAVELGELERYCKHNADIDYSAILDKTVEPHVRLSSVAPPSGRH
PAMLMCSAYDFYPKPIRVTWLRDGREVKSDVTSTEE
>AS10_DAB1_s1_74482
VSLTLVLSIFSGTDGYFEQVVRQCRYSSKDLQGIEFIDSYVFNKAEYVRFNSTVGKYVGY
TELGVKNAEAWNKGPELAVELGELERYCKLNAPIDYSAILDKTVEPHVRLSSVAPPSGRH
PAMLMCSAYDFYPKPIRVTWLRDGREVKSDVTSTEE
>AS10_DAB1_s2_380
VSLTLVLSIFSGTDGYFEQVVRQCRYSSKDLQGIEFIDSYVFNKAEYVRFNSTVGKYVGY
TELGVKNAEAWNKGPELAVELGELERYCKLNAPIDYSAILDKTVEPHVRLSSVAPPSGRH
PAMLMCSAYDFYPKPIRVTWLRDGREVKSDVTSTEE
>AS10_DAB1_s3_353
VSLTLVLSIFSGTDGYFEQVVRQCRYSSKDLQGIEFIDSYVFNKAEYVRFNSTVGKYVGY
TELGVKNAEAWNKGPELAVELGELERYCKLNAPIDYSAILDKTVEPHVRLGSVAPPSGRH
PAMLMCSAYDFYPKPIRVTWLRDGREVKSDVTSTEE
>AS10_DAB1_s4_342
VSLTLVLSIFSGTDGYFEQVVRQCRYSSKDLQGIEFIDSYVFNKAEYVRFNSTVGKYVGY
TELGVKNAEAWNKGPELAVELGELERYCKLNAPIDYSAILDKTVEPHVRLSSVAPPSGRH
PAMLMCSAYDFYPKPIRVTWLRDGRGVKSDVTSTEE
>AS10_DAB1_s5_337
VSLTLVLSIFSGTDGYFEQVVRQCRYSSKDLQGIEFIDSYVFNKAEYVRFNSTVGKYVGY
TELGVKNAEAWNKGPELAVELGELERYCKLNAPIDYSAILDKTVEPHVRLSSVAPPSGRH
PAMLMCSAYDFYPKPIRVAWLRDGREVKSDVTSTEE
```

Deduced MHC class I alpha (UBA) sequences:

```
>AS1_UBA1_s1_30927
TASAATHSLKYFYTAVSGDIDFPEFTIVGLVNNGQFVYYDSNIKRMVPKTEWMKQSAGAD
YWDTESEKQVGQNQGFKNNIQVLKDRFNQSMSTGVHVFQVMYGCEWDDEAGATEGFDQYG
YDGEDFLAFDLKTLKWIAPTPQAVITKLKWDSDTAQNEYRKNYYTQTCIE
>AS1_UBA1_s2_203
TASAATHSLKYFYTAVSGVHVFQVMYGCEWDDEAGATEGFDQYGYDGEDFLAFDLKTLKW
IAPTPQAVITKLKWDSDTAQNEYRKNYYTQTCIE
>AS1_UBA1_s369
TASAATHSLKYFYTAVSGDIDFPEFTIVGLVNNGQFVYYDSNIKRMVPKTEWMKQSAGAD
YWDTESEKQVGQNQGFKNNIQVLKDRFNQSMSTGVHVCQVMYGCEWDDEAGATEGFDQYG
YDGEDFLAFDLKTLKWIAPTPQAVITKLKWDSDTAQNEYRKNYYTQTCIE
>AS1_UBA1_s461
TASAATHSLKYFYTAVSGDIDFPEFTIVGLVNNGQFVYYDSNIKRMVPKTEWMKQSAGAD
YWDTESEKQVGQNQGFKNNIQVLKDRFNQSMSTGVHVFQVMYGCEWDDEAGATEGFDQYG
YDGEDFLAFDLKTLKWIAPTPQAVITKLKWDSGTAQNEYRKNYYTQTCIE
>AS1_UBA1_s553
TASAATHSLKYFYTAVSGDIDFPEFTIVGLVNNGQFVYYDSNIKRMVPKTEWMKQSAGAD
YWDTESGKQVGQNQGFKNNIQVLKDRFNQSMSTGVHVFQVMYGCEWDDEAGATEGFDQYG
YDGEDFLAFDLKTLKWIAPTPQAVITKLKWDSDTAQNEYRKNYYTQTCIE
>AS2_UBA1_s1_46437
TASAVTHALKYFYTASSEVPNFPEFVVVAMVDGVQMVHYDSNSQRAVPKQDWVNKAADPQ
YWERNTGIFKGSQQTFKANIDIVKQRFNQSGGVHIYQNMYGCEWDDEAGVTEGFDQYGYD
GEDFLAFDLKTLKWIAPTPQSLITKLKWDNNMAQIQQDKHYLTQTCIE
>AS2_UBA1_s2_33647
TASAVTHALKYFYTASSEVPNFPEFVVVGVVDGVQMVHYDSNSQRAVPKQDWVNKAADPQ
YWERNTGNCKGSQQIFKANIDIVKQRFNQSGGVHVNQNMYGCEWDDEAGVTEGFDQYGYD
GEDFLAFDLKTLKWIAPTPQSLITKLKWDNNMAQIQQDKHYLTQTCIE
>AS2_UBA1_s3_8559
TASAVTHALKYFYTASSEVPNFPEFVVVAMVDGVQMVHYDSNSQRAVPKQDWVNKAADPQ
YWERNTGNCKGSQQIFKANIDIVKQRFNQSGGVHVNQNMYGCEWDDEAGVTEGFDQYGYD
GEDFLAFDLKTLKWIAPTPQSLITKLKWDNNMAQIQQDKHYLTQTCIE
>AS2_UBA1_s4_7940
```

```
TASAVTHALKYFYTASSEVPNFPEFVVVGVVDGVQMVHYDSNSQRAVPKQDWVNKAADPQ
YWERNTGIFKGSQQTFKANIDIVKQRFNQSGGVHIYQNMYGCEWDDEAGVTEGFDQYGYD
GEDFLAFDLKTLKWIAPTPQSLITKLKWDNNMAQIQQDKHYLTQTCIE
>AS2_UBA1_s5_4195
TASAVTHALKYFYTASSEVPNFPEFVVVAMVDGVQMVHYDSNSQRAVPKQDWVNKAADPQ
YWERNTGIFKGSQQTFKANIDIVKQRFNQSGGVHVNQNMYGCEWDDEAGVTEGFDQYGYD
GEDFLAFDLKTLKWIAPTPQSLITKLKWDNNMAQIQQDKHYLTQTCIE
>AS3_UBA1_s1_13465
TASAATHSLKYFYTAVSGDIDFPEFTIVGLVNNGQFVYYDSNIKRMVPKTEWMKQSAGAD
YWDTESEKQVGQNQGFKNNIQVLKDRFNQSMSTGVHVFQVMYGCEWDDEAGATEGFDQYG
YDGEDFLAFDLKTLKWIAPTPQAVITKLKWDSDTAQNEYRKNYYTQTCIE
>AS3_UBA1_s2_12935
TASAVTHSLKYFYTASSEVPNFPEFVVVSMVDGVQMVHYDSNSQRAVPKQDWMNKAAEAL
PQYWDIETGKFLGSHQSFKANIDICKQRFNQSGGVHIVQKMYGCEWDDETGVTEGFNQYG
YDGEDFIAFDLKTLKWIAPTPQAVITKLKWDSDTAQNEYRKNYYTQTCIE
>AS3_UBA1_s3_609
TASAVTHSLKYFYTASSEVPNFPEFVVVSMVDGVQMVHYDSNSQRAVPKQDWMNKAAEAL
PQYWDIETGKFLGSHQSFKANIDICKQRFNQSGGVHIVQKMYGCEWDDETGVTEGFNQYG
YDGEDFLAFDLKTLKWIAPTPQAVITKLKWDSDTAQNEYRKNYYTQTCIE
>AS3_UBA1_s4_609
TASAATHSLKYFYTAVSGDIDFPEFTIVGLVNNGQFVYYDSNIKRMVPKTEWMKQSAGAD
YWDTESEKQVGQNQGFKNNIQVLKDRFNQSMSTGVHVFQVMYGCEWDDEAGATEGFDQYG
YDGEDFIAFDLKTLKWIAPTPQAVITKLKWDSDTAQNEYRKNYYTQTCIE
>AS3_UBA1_s5_288
TASAVTHSLKYFYTASSEVPNFPEFVVVSMVDGVQMVHYDSNSQRAVPKQDWMNKAAEAL
PQYWDIETGKFLGSHQSFKANIDICKQRFNQSGGVHIVQKMYGCEWDDEAGATEGFDQYG
YDGEDFLAFDLKTLKWIAPTPQAVITKLKWDSDTAQNEYRKNYYTQTCIE
>AS5_UBA2_s1_22247
LKYFYTGSTGIEGFPQFVAVGIVDGMHIDYFDSVSEKNVLKQSWMEGARDEKSITNIRKG
NQQSFKANVEIVMQRFNQTTGVHVFQNMYGCEWDDETGVTEGFDQDGYDGEDFLAFDLKT
LTWIAPTPQAVNTKHKWDSNTAYNEQEKNYLTQICIE
>AS5_UBA2_s2_20661
LKYFYTGSTGIEGFPQFVAVGIVDGMHIDYFDSVSEKNVLKQSWMEGARDEKSITNIRKG
NQQSFKANVEIVMQRFNQTTGVHVNQWMYGCEWDDEAGVTEGFEQWGYDGEDFIAFDLKT
KSWIAPTPQAVITKLKWDSDTAQNEHRKNYYTQICIE
>AS5_UBA2_s3_576
LKYFYTGSTGIEGFPQFVAVGIVDGMHIDYFDSVSEKNVLKQSWMEGARDEKSITNIRKG
NQQSFKANVEIVMQRFNQTTGVHVFQNMYGCEWDDETGVTEGFDQDGYDGEDFLAFDLKT
LTWIAPTPQAVITKLKWDSDTAQNEHRKNYYTQICIE
>AS5_UBA2_s4_489
LKYFYTGSTGIEGFPQFVAVGIVDGMHIDYFDSVSEKNVLKQSWMEGARDEKSITNIRKG
NQQSFKANVEIVMQRFNQTTGVHVFQNMYGCEWDDETGVTEGFEQWGYDGEDFIAFDLKT
KSWIAPTPQAVITKLKWDSDTAQNEHRKNYYTQICIE
>AS5_UBA2_s5_480
LKYFYTGSTGIEGFPQFVAVGIVDGMHIDYFDSVSEKNVLKQSWMEGARDEKSITNIRKG
NQQSFKANVEIVMQRFNQTTGVHVNQWMYGCEWDDEAGVTEGFDQDGYDGEDFLAFDLKT
LTWIAPTPQAVNTKHKWDSNTAYNEQEKNYLTQICIE
>AS6_UBA1_s1_18686
TASAVTHSLKYFYTASSEVPNFPEFVVVSMVDGVQMVHYDSNSQRAVPKQDWMNKAAEAL
PQYWDIETGKFLGSHQSFKANIDICKQRFNQSGGVHIVQKMYGCEWDDETGVTEGFNQYG
YDGEDFIAFDLKTLKWIAPTPQAVITKLKWDSDTAQNEYRKNYYTQTCIE
>AS6_UBA1_s2_3956
TVSAATNTLQYFYTATSGIDNFPEFVTMGIVNGHQIDHYDSITKRAIQKAEWISGAVDPD
YWKTNTQIYAGTETVFVNNINVAKSRFNQTGGVHVNQKMYGCEWDDETGVTEGFDQDGYD
GEDFLAFDLKTLTWIAPTPQAVITKLKWDSNTAQNEYRKNYLTQTCIE
>AS6_UBA1_s3_399
TVSAATNTLQYFYTATSGIDNFPEFVTMGIVNGHQIDHYDSITKRAIQKAEWISGAVDPD
YWKTNTQIYAGTETVFVNNINVAKSRFNQTGGVHVNQKMYGCEWDDETGVTEGFDQDGYD
GEDFLAFDLKTLTWIAPTPQAVITKLKWDSDTAQNEYRKNYYTQTCIE
>AS6_UBA1_s4_297
TASAVTHSLKYFYTASSEVPNFPEFVVVSMVDGVQMVHYDSNSQRAVPKQDWMNKAAEAL
```

PQYWDIETGKFLGSHQSFKANIDICKQRFNQSGGVHIVQKMYGCEWDDETGVTEGFNQYG
YDGEDFIAFDLKTLKWIAPTPQAVITKLKWDSDTAQNEYRKNYLTQTCIE
>AS6_UBA1_s5_287
TVSAATNTLQYFYTATSGIDNFPEFVTMGIVNGHQIDHYDSITKRAIQKAEWISGAVDPD
YWKTNTQIYAGTETVFVNNINVAKSRFNQTGGVHVNQKMYGCEWDDETGVTEGFDQDGYD
GEDFLAFDLKTLTWIAPTPQAVITKLKWDSNTAQNEYRKNYYTQTCIE
>AS7_UBA1_s1_25193
TASAVTHALKYFYTASSEVPNFPEFVAVGVVDGVQMFHYDSNSQRAVPKQDWVNKAADPQ
YWERNTGNCKGSQQIFKANIDIVKQRFNQSGGVHVFQNMYGCEWDDEAGVTEGFDQYGYD
GEDFIAFDLKTKTWIAPKTQAVNTKNKWDSDTAQNEFLKNYYTQTCIE
>AS7_UBA1_s2_6454
TVSAATNTLQYFYTATSGIDNFPEFVTMGIVNGHQIDHYDSITKRAIQKAEWISGAVDPD
YWKTNTQIYAGTETVFVNNINVAKSRFNQTGGVHVFQMMCGCEWDDEAGATEGFDQYGYD
GEDFIAFDLKTKSWIAPTPQAVITKLKWDSNTAQNEYWKNYLTQECIE
>AS7_UBA1_s3_481
TVSAATNTLQYFYTATSGIDNFPEFVTMGIVNGHQIDHYDSITKRAIQKAEWISGAVDPD
YWKTNTQIYAGTETVFVNNINVAKSRFNQTGGVHVFQMMCGCEWDDEAGATEGFDQYGYD
GEDFIAFDLKTKTWIAPKTQAVNTKNKWDSDTAQNEFLKNYYTQTCIE
>AS7_UBA1_s4_289
TASAVTHALKYFYTASSEVPNFPEFVAVGVVDGVQMFHYDSNSQRAVPKQDWVNKAADPQ
YWERNTGNCKGSQQIFKANIDIVKQRFNQSGGVHVFQNMYGCEWDDEAGVTEGFDQYGYD
GEDFIAFDLKTKSWIAPTPQAVITKLKWDSNTAQNEYWKNYLTQECIE
>AS7_UBA1_s5_171
TASAVTHALKYFYTASSEVPNFPEFVAVGVVDGVQMFHYDSNSQRAVPKQDWVNKAADPQ
YWERNTGNCKGSQQIFKANIDIVRQRFNQSGGVHVFQNMYGCEWDDEAGVTEGFDQYGYD
GEDFIAFDLKTKTWIAPKTQAVNTKNKWDSDTAQNEFLKNYYTQTCIE
>AS8_UBA1_s1_30959
TASAVTHSLKYFYTASSEVPNFPEFVVVSMVDGVQMVHYDSNSQRAVPKQDWMNKAAEAL
PQYWDIETGKFLGSHQSFKANIDICKQRFNQSGGVHIVQKMYGCEWDDETGVTEGFNQYG
YDGEDFIAFDLKTLKWIAPTPQAVITKLKWDSDTAQNEYRKNYYTQTCIE
>AS8_UBA1_s2_24959
TASAATHSLKYFYTAVSGDIDFPEFTIVGLVNNGQFVYYDSNIKRMVPKTEWMKQSAGAD
YWDTESEKQVGQNQGFKNNIQVLKDRFNQSMSTGVHVNQWMYGCEWDDEAGVTEGFEQWG
YDGEDFIAFDLKTKSWIAPTPQAVITKLKWDSDTAQNEHRKNYYTQICIE
>AS8_UBA1_s3_2725
TASAVTHSLKYFYTASSEVPNFPEFVVVSMVDGVQMVHYDSNSQRAVPKQDWMNKAAEAL
PQYWDIETGKFLGSHQSFKANIDICKQRFNQSGGVHIVQKMYGCEWDDETGVTEGFNQYG
YDGEDFIAFDLKTLKWIAPTPQAVITKLKWDSDTAQNEHRKNYYTQICIE
>AS8_UBA1_s4_2424
TASAATHSLKYFYTAVSGDIDFPEFTIVGLVNNGQFVYYDSNIKRMVPKTEWMKQSAGAD
YWDTESEKQVGQNQGFKNNIQVLKDRFNQSMSTGVHVNQWMYGCEWDDEAGVTEGFEQWG
YDGEDFIAFDLKTKSWIAPTPQAVITKLKWDSDTAQNEYRKNYYTQTCIE
>AS8_UBA1_s5_1597
TASAVTHSLKYFYTASSEVPNFPEFVVVSMVDGVQMVHYDSNSQRAVPKQDWMNKAAEAL
PQYWDIETGKFLGSHQSFKANIDICKQRFNQSGGVHIVQKMYGCEWDDETGVTEGFNQYG
YDGEDFIAFDLKTLKWIAPTPQAVITKLKWDSDTAQNEYRKNYYTQICIE
>AS9_UBA1_s1_20357
TASAVTHSLKYFYTASSEVPNFPEFVVVSMVDGVQMVHYDSNSQRAVPKQDWMNKAAEAL
PQYWDIETGKFLGSHQSFKANIDICKQRFNQSGGVHIVQKMYGCEWDDETGVTEGFNQYG
YDGEDFIAFDLKTLKWIAPTPQAVITKLKWDSDTAQNEYRKNYYTQTCIE
>AS9_UBA1_s2_14112
TASAVTHALKYFYTASSEVPNFPEFVAVGVVDGVQMVHYDSNSQRAVPKQDWMNKAAEAL
PQYWDIETGNLLGSHQSFKANIDICKQRFNQSGGVHVNQWMYGCEWDDEAGVTEGFEQWG
YDGEDFIAFDLKTKSWIAPTPQAVITKLKWDSDTAQNEHRKNYYTQICIE
>AS9_UBA1_s3_3551
TASAVTHSLKYFYTASSEVPNFPEFVVVSMVDGVQMVHYDSNSQRAVPKQDWMNKAAEAL
PQYWDIETGNLLGSHQSFKANIDICKQRFNQSGGVHVNQWMYGCEWDDEAGVTEGFEQWG
YDGEDFIAFDLKTKSWIAPTPQAVITKLKWDSDTAQNEHRKNYYTQICIE
>AS9_UBA1_s4_3146
TASAVTHALKYFYTASSEVPNFPEFVAVGVVDGVQMVHYDSNSQRAVPKQDWMNKAAEAL
PQYWDIETGKFLGSHQSFKANIDICKQRFNQSGGVHIVQKMYGCEWDDETGVTEGFNQYG

YDGEDFIAFDLKTLKWIAPTPQAVITKLKWDSDTAQNEYRKNYYTQTCIE
>AS9_UBA1_s5_2549
TASAVTHSLKYFYTASSEVPNFPEFVVVSMVDGVQMVHYDSNSQRAVPKQDWMNKAAEAL
PQYWDIETGKFLGSHQSFKANIDICKQRFNQSGGVHVNQWMYGCEWDDEAGVTEGFEQWG
YDGEDFIAFDLKTKSWIAPTPQAVITKLKWDSDTAQNEHRKNYYTQICIE
>AS10_UBA1_s1_71332
TASAVTHALKYFYTASSEVPNFPEFVVVGVVDGVQMVHYDSNSQRAVPKQDWVNKAADPQ
YWERNTGIFKGSQQTFKANIDIAKQRFNQSGGVHVNQWMYGCEWDDEAGVTEGFEQWGYD
GEDFIAFDLKTKSWIAPTPQAVITKLKWDSDTAQNEHRKNYYTQICIE
>AS10_UBA1_s2_448
TASAVTHSLRYFYTATTGIPDFPEFVDVGVVNGKVISYYDSIIKRKVPKQSWMEENLNQQ
YWNQGTDQLKGTEQSFKANIQVAQTRFNQTGGVHIFQYMYGCTWDDDSGVTDGLRQYGYD
GEDFLVYDMKAFTWIAPKLQAEITTRKWNNEPAQMEYLKSYITQECVE
>AS10_UBA1_s3_331
TASAVTHALKYFYTASSEVPNFPEFVVVGVVDGVQMVHYDSNSQRAVPKQDWVNKAADPQ
YWERNTGIFKGSQQTFKANIDIARQRFNQSGGVHVNQWMYGCEWDDEAGVTEGFEQWGYD
GEDFIAFDLKTKSWIAPTPQAVITKLKWDSDTAQNEHRKNYYTQICIE
>AS10_UBA1_s4_325
TASAVTHALKYFYTASSEVPNFPEFVVVGVVDGAQMVHYDSNSQRAVPKQDWVNKAADPQ
YWERNTGIFKGSQQTFKANIDIAKQRFNQSGGVHVNQWMYGCEWDDEAGVTEGFEQWGYD
GEDFIAFDLKTKSWIAPTPQAVITKLKWDSDTAQNEHRKNYYTQICIE
>AS10_UBA1_s5_322
TASAVTHALKYFYTASSEVPNFPEFVVVGVVDGVQMVHYDSNSQRAVPKQDWVNKAADPQ
YWERNTGIFKGSQQTFKANIDIAKQRFNQSGGVHVNQGMYGCEWDDEAGVTEGFEQWGYD
GEDFIAFDLKTKSWIAPTPQAVITKLKWDSDTAQNEHRKNYYTQICIE
