# Supplementary material for: An Illumina approach to MHC typing of Atlantic salmon
Source: Immunogenetics. 2019 Nov 12;72(1-2):89–100. doi: 10.1007/s00251-019-01143-8 (PMC6970960; doi:10.1007/s00251-019-01143-8)
Supplement: Supplementary file 4 — UBA primer regions (PDF 543 kb) [file 251_2019_1143_MOESM4_ESM.pdf]

Supplementary file 4. Nucleotide sequence alignment of Atlantic salmon MHC class I UBA sequences.

Alignment of Atlantic salmon MHC class I UBA nucleotide sequences containing entire open reading frames. Dots indicate identity, while dashes show missing sequence. Individual domains and amino acid translation are shown above the sequence. Nucleotide numbering on top relates to the UBA*0101 sequence. Primer sequences tested on the study material are colour coded as follows: Forward primers pUBA1F is green, pUBA2F is purple, pUBA3F is gray, pUBA4F is yellow, pUBA5F is cyan, while both the reverse primer used for Illumina (pUBAR) and the reverse primer to be used for Sanger amplification (pUBAR.Tail) are shaded yellow. Lines with no sequence are not shown. Sequence accession number are as follows: UBA*01:01 AAN75113.1, UBA*03:01 AAN75116.1, UBA*33:01 AAZ73110.1, TSA#1=GBHC01001870.1, TSA#2 9GEGX01184951.1.

```
            Leader sequence                                               Alpha 1 domain
                 Individual forward primer reions
                     *            20        *         40        *          60
             M  K  G  F  I  L     L  V  L  G  I  G  L  L  H  T  A  S  A   V  T  H
UBA*01:01 : atgaaaggttttatcttg---ctggtgctgggaataggccttctacatacagcatctgctg tgactcacg :    67
TSA#1     : .....g...gg....c.c---a..t.c..........tg.ac..t.g.ga........... ......... :    67
TSA#2     : .....ga...g....c.tctg...t.c..........t......t.......t........ ca..ca.ta :    70
UBA*03:01 : .....gt....c..tc.t---...c.tt.a.......---cc......t.tt.......c. ct..g..tt :    64
UBA*33:01 : ------ttca.c..t...------c.c........---------ta..g.g........a. .a..c...t :    49

              *         80        *         100       *         120       *
            A  L  K  Y  F  Y  T  A  S  S  E  V  P  N  F  P  E  F  V  V  V  G  V  V
UBA*01:01 : ccctgaagtatttctacaccgcatcttctgaagttcccaacttcccagagtttgtagttgtgggggtggtg :   138
TSA#1     : .......................................................g.c............. :   138
TSA#2     : .a...c.............g..ca......gta.agat.................gac.a....aa.c..c :   141
UBA*03:01 : .a...cgt...g.g.....t..ga.c..a.g.a.a..ag................gac...t...t..... :   135
UBA*33:01 : .t.................t.g.ag.a...gca..gagggg......c.......t.ca.....ca.c..a :   120

             140       *         160       *         180       *         200
             D  G  V  Q  M  V  H  Y  D  S  N  S  Q  R  A  V  P  K  Q  D  W  V  N  K
UBA*01:01 : gatggtgttcagatggttcactatgacagcaacagccagagagcggtgcccaaacaggactgggtaaacaa :   209
TSA#1     : ....................................................................... :   209
TSA#2     : a...cca.......t.ac.....c.....t.t..c.a......aa.c.ag...gct...g...a.c.gtgg :   212
UBA*03:01 : a...a.aa.cc...ctcgt............t..t..gc....aaact...cg........a.ggc..... :   206
UBA*33:01 : ....aa.g...c..t.act...t...t..tgtgtctg...agaat..t.tg..g...tct...a.gg.ggg :   191

            *         220       *         240       *         260       *         280
              A  A  D  P  Q  Y  W  E  R  N  T  G  I  F  K  G  S  Q  Q  T  F  K  A
UBA*01:01 : ggcagcagacccacagtactgggagaggaacactgggattttcaagggttcccagcagactttcaaagcca :   280
TSA#1     : ......................................a..g.................t........... :   280
TSA#2     : .....tt.....tg.c..t...a...ct.....cca...c.atgct...a.ag..acagt....gtgaa.. :   283
UBA*03:01 : .a.t.ag.ggt.tg.c..........tc..g..ca.g.c.c...tt.....tg.a.....c.......... :   277
UBA*33:01 : ..---------...gtg.tgaaa.a..c.t...aaac..aaga....ccat....a.gc..t..g...... :   253

                                                   Alpha 2 domain
                     *         300       *          320       *         340       *
            N  I  D  I  A  K  Q  R  F  N  Q  S  G   G  V  H  V  N  Q  W  M  Y  G  C
UBA*01:01 : acatcgatattgcaaagcagcgttttaaccaaagtgga ggtgtgcacgttaaccagtggatgtatggatg :   350
TSA#1     : ............t......................... .........a..t.....aat........... :   350
TSA#2     : ....ta..g....g..atcc..c..c..t....c.... ..................aa............ :   353
UBA*03:01 : ....t...g....c........c..c..t..g.c.... ..................aa............ :   347
UBA*33:01 : .tg....a..c.t..t...a.....c.......caac. ............tt....aa............ :   323

                     360       *         380       *         400       *         420
              E  W  D  D  E  A  G  V  T  E  G  F  E  Q  W  G  Y  D  G  E  D  F  I
UBA*01:01 : tgagtgggatgatgaggctggagtcacagaggggtttgaacagtggggatatgatggagaggacttcatag :   421
TSA#1     : .......................................t....at.................t...t... :   421
TSA#2     : ................a....................a.t....at.................t....... :   424
UBA*03:01 : ................a......................t...gat.................t...t... :   418
UBA*33:01 : .......................c............gact....at.................t....... :   394
```

```
                    *         440       *         460       *         480       *
            A  F  D  L  K  T  K  S  W  I  A  P  T  P  Q  S  V  I  T  K  L  K  W  D
UBA*01:01 : catttgacctgaagacaaagtcatggatcgccccaacccacaggtcagtcatcaccaaactcaagtgggac :  492
TSA#1     : .................tt.aa...............g.caca....c......................t :  492
TSA#2     : .................tt.aa...............g.caca.g.......................... :  495
UBA*03:01 : .............a...tt.a................g.caca.g.......................... :  489
UBA*33:01 : .....................................g.caca....c....................... :  465

                                                                        pUBAR primer
                   500       *         520       *         540       *         560
             S  D  T  A  Q  N  E  H  D  K  H  Y  L  T  Q  T  C  I  E  W  L  K  K  Y
UBA*01:01 : agtgacacagctcagaatgagcacgataaacattacctcacccagacctgcattgagtggctgaagaagta :  563
TSA#1     : .a.a...tg.......tac....g............................................... :  563
TSA#2     : .....................t..cga...a.c...ta................................. :  566
UBA*03:01 : ...a.................t..cga...a.c...................................... :  560
UBA*33:01 : ............t.c........g..a...a.c.............t........................ :  536
                                                Alpha 3 domain
                  *         580       *          600       *         620       *
              L  D  Y  G  K  S  T  L  M  R  T   V  P  P  S  V  S  L  L  Q  K  T  P
UBA*01:01 : tctggactatgggaagagcactctgatgaggacag tccctccgtcagtgtctctgctccagaagaccccc :  633
TSA#1     : .g................................. ................................... :  633
TSA#2     : .g................................. ................................... :  636
UBA*03:01 : ................................... ....c..c........................... :  630
UBA*33:01 : .g................................. ................................... :  606

                  640       *         660       *         680       *         700
             S  S  P  V  T  C  H  A  T  G  F  Y  P  S  G  V  M  V  S  W  Q  K  D  G
UBA*01:01 : tcctctccagtgacctgccacgcgacaggtttctaccccagtggagtcatggtgtcctggcagaaagacgg :  704
TSA#1     : ....................................................................... :  704
TSA#2     : ....................................................................... :  707
UBA*03:01 : ....................................................................... :  701
UBA*33:01 : ....................................................................... :  677

                 *         720       *         740       *         760       *
              Q  D  H  H  E  D  V  E  Y  G  E  T  L  Q  N  D  D  G  T  F  Q  K  S
UBA*01:01 : acaagatcaccatgaagatgtggagtacggagagactctccagaacgatgacggaaccttccagaaaagct :  775
TSA#1     : ....................................................................... :  775
TSA#2     : ....................................................................... :  778
UBA*03:01 : .........................c.t.......................t................... :  772
UBA*33:01 : ....................................................................... :  748

                780       *         800       *         820       *         840
            S  H  L  T  V  T  P  E  E  W  K  N  N  K  Y  Q  C  V  V  Q  V  T  G  V
UBA*01:01 : cccacctgacagtgacacctgaggagtggaagaacaacaagtatcagtgtgtggttcaagtcacgggtgtc :  846
TSA#1     : ................g...................................................c.. :  846
TSA#2     : ................g...................................................c.. :  849
UBA*03:01 : ................g...................................................c.. :  843
UBA*33:01 : ....................................................................... :  819

                                        Connecting peptide
               *         860       *          880       *         900       *
             K  E  D  F  I  K  V  L  T   E  S  E  I  K  T  N  W  N  E  P  N  I  V
UBA*01:01 : aaggaggacttcatcaaggttctgact gagtctgagatcaagaccaactggaatgagcccaacattgtcc :  916
TSA#1     : ........................... .............................c............. :  916
TSA#2     : c.......................... .............................c............. :  919
UBA*03:01 : c.......................... .............................c............. :  913
UBA*33:01 : ........................... .............................c............. :  889

            Transmembrane region
               920       *         940       *               960       *         980
            L  I  I  V  V  V  V  A  L  L  L  L  V  V  A        V  V  V  G  V  V  I
UBA*01:01 : tcatcattgtagtggtggtagctctcctcctgctcgtcgttgc------tgttgttgttggggtcgtcatt :  981
TSA#1     : ................................g..........------...................... :  981
TSA#2     : ...........................................tgttgt...................... :  990
UBA*03:01 : .........g......................g..........------...................... :  978
UBA*33:01 : ...........................................------...................... :  954
```

```
                **Cytoplasmic domain**
                       *        1000       *        1020       *        140        *
                W   K  K  K  S  K  K  G  F  V  P  A  S  T  S  D  T  D  S  D  N  S  G
UBA*01:01 : tgg aagaagaagagcaagaaaggctttgttccggccagcacttccgacactgactctgacaactctggga : 1050
TSA#1     : ... ................................................................... : 1051
TSA#2     : ... ................................................................... : 1060
UBA*03:01 : ... ................................................................... : 1048
UBA*33:01 : ... ................................................................... : 1024

                                 **Stopp codon**
                      1060       *        1080       *        1100       *         1120
              R  A  A  Q  M  T
UBA*01:01 : gagctgcccagatgactTGA gagacctctctgctcttctgtagcatcagagaaggtgctgaagaggaacg : 1120
TSA#1     : .................... .................................................. : 1121
TSA#2     : .................... .................................................. : 1130
UBA*03:01 : .................... -------------------------------------------------- : 1068
UBA*33:01 : .................... -------------------------------------------------- : 1044

                      *        1140       *        1160       *        1180
UBA*01:01 : tctcaagaacataacacacgtatacttgcacacacacacacacacacacacacacacacaacaca---- : 1187
TSA#1     : ..............................................................-....acac : 1191
TSA#2     : ....................................................------------------- : 1182

                                                 pUBAR.tail
                 *        1200       *        1220       *
UBA*01:01 : -ggaggttggtgccaccttaattgggtcgagcgggcttgtggtaattg : 1234
TSA#1     : a......----------------------------------------- : 1198
```
