# Supplementary material for: An Illumina approach to MHC typing of Atlantic salmon
Source: Immunogenetics. 2019 Nov 12;72(1-2):89–100. doi: 10.1007/s00251-019-01143-8 (PMC6970960; doi:10.1007/s00251-019-01143-8)
Supplement: Supplementary file 5 — MHC sequence alignments (PDF 1303 kb) [file 251_2019_1143_MOESM5_ESM.pdf]

Supplementary file 5. Alignment of deduced MHC amino acid sequences from Atlantic salmon.

Table of Contents		Page
SF5A	Alignment of deduced MHC class II alpha amino acid sequences	1
SF5B	Alignment of deduced MHC class II beta amino acid sequences	5
SF5C	Alignment of deduced MHC class I amino acid sequences	9

Supplementary Figure 5A. Alignment of deduced Atlantic salmon MHC class II alpha DAA amino acid sequences

Deduced amino acid alignment of Atlantic salmon MHC class II alpha sequences. Dots indicates sequence identity, dashes show missing sequence. Amino acids are numbered above the sequence alignment according to the DAA*01:01 sequence. Individual domains are shown above the alignment. Lines without sequence are not shown. Location of primer sequences used to amplify fragments for Illumina and Sanger sequencing are indicated using green color. CP is connecting peptide, TM is transmembrane region and CYT is cytoplasmic region.

```
             Leader sequence     Alpha 1 domain
                       Illumina/ Sanger Forward primer
                       *
DAA*01:01   : MKTSVIVLILCWQVYAEHK- :  19
DAA*01:02   : ----------.........- :   9
AS3_DAA_s1  : -----------------..- :   2
AS8_DAA_s2  : -----------------..- :   2
DAA*02:01   : ----------.........- :   9
AS6_DAA_s1  : -----------------..- :   2
AS7_DAA_s2  : -----------------..- :   2
DAA*03:01   : ----------.........- :   9
DAA*03:02   : -----------------..- :   2
AS2_DAA_s2  : -----------------..- :   2
AS9_DAA_s1  : -----------------..- :   2
DAA*03:03:01: -----------------..- :   2
DAA*04:01   : ----------.........- :   9
AS1_DAA_s2  : -----------------..- :   2
AS8_DAA_s1  : -----------------..- :   2
AS10_DAA_s1 : -----------------..- :   2
DAA*05:01   : ----------.........- :   9
DAA*05:02   : ----------.........- :   9
DAA*06:01   : ----------.........- :   9
AS1_DAA_s1  : -----------------..- :   2
AS2_DAA_s1  : -----------------..- :   2
AS5_DAA_s1  : -----------------..- :   2
AS7_DAA_s1  : -----------------..- :   2
DAA*07:01   : ----------.........- :   9
DAA*08:01   : -----------------..- :   2
DAA*09:01   : ------.............- :  13
AS3_DAA_s2  : -----------------..- :   2
AS6_DAA_s2  : -----------------..- :   2
AS9_DAA_s2  : -----------------..- :   2
DAA*10:01   : ------.............- :  13
DAA*11:01   : -----------------..- :   2
DAA*12:01   : -----------------..- :   2
DAA*13:01   : ----------.........- :   9
DAA*14:01   : ----------.........- :   9
```

```
Alpha 1 domain
              20        *         40        *         60        *         80        *        100
DAA*01:01   : VLHIDLVITGCSDSDGLDMYGLDGEEMWYADFNKQEGVVALPPFADPFTFPGFYEQAVGNQGVCKGNLAKCIKAYKNPEEKI : 101
DAA*01:02   : .................................................................................. :  91
AS3_DAA_s1  : .................................................................................. :  84
AS8_DAA_s2  : .................................................................................. :  84
DAA*02:01   : ......Y.S.........................G....................G.........A...VN........... :  91
AS6_DAA_s1  : ......Y.S.........................G....................G.........A...VN........... :  84
AS7_DAA_s2  : ......Y.S.........................G....................G.........A...VN........... :  84
DAA*03:01   : ................V.................G...MP.........Y..A..G......I..A...T............ :  91
DAA*03:02   : ......A...........................G...MP.........Y..A..G......I..A...T............ :  84
AS2_DAA_s2  : ......A...........................G...MP.........Y..A..G......I..A...T............ :  84
AS9_DAA_s1  : ......A...........................G...MP.........Y..A..G......I..A...T............ :  84
DAA*03:03:01: .................N................G...MP.........Y..A..G......I..A...T............ :  84
DAA*03:03:02: .................N................G...MP.........Y..A..G......I..A...T............ :  82
DAA*03:04   : ---------.......V.................G...MP.........Y..A............A...T.........--- :  70
DAA*03:05   : ---------........N................G...MP.........Y..A..R.........A...I.........--- :  70
DAA*04:01   : ......H.I.......V.................G....................G.........A...VN........... :  91
AS1_DAA_s2  : ......H.I.......V.................G....................G.........A...VN........... :  84
AS8_DAA_s1  : ......H.I.......V.................G....................G.........A...VN........... :  84
AS10_DAA_s1 : ......H.I.......V.................G....................G.........A...VN........... :  84
DAA*05:01   : ......A.........V.................G.................H..G.........A...TS........... :  91
DAA*05:02   : ......A.........V.................G..................................TS........... :  91
DAA*06:01   : ........S.......V.................G...MP.........Y..A............A...VN........... :  91
AS1_DAA_s1  : ........S.......V.................G...MP.........Y..A............A...VN........... :  84
AS2_DAA_s1  : ........S.......V.................G...MP.........Y..A............A...VN........... :  84
AS5_DAA_s1  : ........S.......V.................G...MP.........Y..A............A...VN........... :  84
AS7_DAA_s1  : ........S.......V.................G...MP.........Y..A............A...VN........... :  84
DAA*07:01   : ......A..........................................YH.A.........I................... :  91
DAA*08:01   : ......A.............................................H..G.......................... :  84
DAA*09:01   : ................V.................G...............H.A..G.........A...VN........... :  95
AS3_DAA_s2  : ................V.................G...............H.A..G.........A...VN........... :  84
AS6_DAA_s2  : ................V.................G...............H.A..G.........A...VN........... :  84
AS9_DAA_s2  : ................V.................G...............H.A..G.........A...VN........... :  84
DAA*10:01   : ......A..........N................G..............................A...TS........... :  95
DAA*11:01   : ......A.........V..........S......G..............................A...TS........... :  84
DAA*12:01   : ......H.........VE................G...MP.........Y..A.........I..A...VN........... :  84
DAA*12:02   : ---------.......VE................G...MP............A............A...VN........--- :  70
DAA*13:01   : ........S.......V.................G.................A............A...VN........... :  91
DAA*14:01   : ...K..Y...........................G..............................A...T............ :  91
```

```
Alpha 2 domain
                                                                 Illumina reverse primer
                       *         120       *         140       *         160       *         180       *
DAA*01:01   : DPPHSSIYPRDDVLGVENTLICHVSGFFPAPVRVRWTRNNQNLTEGVRLSTPYPNADFTLNQFSSLPFTPEEGDIYGCTVEHKGLAEPLTRIW : 195
DAA*01:02   : ............................................................................................. : 185
AS3_DAA_s1  : ....................................................----------------------------------------- : 137
AS8_DAA_s2  : ....................................................----------------------------------------- : 137
DAA*02:01   : ............................H................................................................ : 185
AS6_DAA_s1  : ............................H.......................----------------------------------------- : 137
AS7_DAA_s2  : ............................H.......................----------------------------------------- : 137
DAA*03:01   : A...........................H................................................................ : 185
DAA*03:02   : A...........................H................................................................ : 178
AS2_DAA_s2  : A...........................H.......................----------------------------------------- : 137
AS9_DAA_s1  : A...........................H.......................----------------------------------------- : 137
DAA*03:03:01: A...........................H................................................................ : 178
DAA*03:03:02: --------------------------------------------------------------------------------------------- :   -
DAA*03:04   : --------------------------------------------------------------------------------------------- :   -
DAA*03:05   : --------------------------------------------------------------------------------------------- :   -
DAA*04:01   : ............................................................................................. : 185
AS1_DAA_s2  : ....................................................----------------------------------------- : 137
AS8_DAA_s1  : ....................................................----------------------------------------- : 137
AS10_DAA_s1 : ....................................................----------------------------------------- : 137
DAA*05:01   : A...........................Y............................V.F................................. : 185
DAA*05:02   : A...........................Y............................V.F................................. : 185
DAA*06:01   : A...........................Y............................V.F................................. : 185
AS1_DAA_s1  : A...........................Y.......................----------------------------------------- : 137
AS2_DAA_s1  : A...........................Y.......................----------------------------------------- : 137
AS5_DAA_s1  : A...........................Y.......................----------------------------------------- : 137
AS7_DAA_s1  : A...........................Y.......................----------------------------------------- : 137
DAA*07:01   : ............................H........A....................................................... : 185
DAA*08:01   : ............................H................................................................ : 178
DAA*09:01   : ............................H................................................................ : 189
AS3_DAA_s2  : ............................H.......................----------------------------------------- : 137
AS6_DAA_s2  : ............................H.......................----------------------------------------- : 137
AS9_DAA_s2  : ............................H.......................----------------------------------------- : 137
DAA*10:01   : A...........................H................................................................ : 189
DAA*11:01   : A...........................H................................................................ : 178
DAA*12:01   : ............................H................................................................ : 178
DAA*12:02   : --------------------------------------------------------------------------------------------- :   -
DAA*13:01   : A........................................................V.F................................. : 185
DAA*14:01   : A...........................H................................................................ : 185
```

```
CP/ TM/ CYT domains
                                             Sanger reverse primer
                  200       *        220          *
DAA*01:01   : EPEVIQPSVGPDVFCGVGLTLGLLGVAAGTFFLI-KGNQCN : 235
DAA*01:02   : ...........A......................-...... : 225
DAA*02:01   : ...........A........V.............-...... : 225
DAA*03:01   : ...........A......................-...... : 225
DAA*03:02   : ...........A......................-...... : 218
DAA*03:03:01: ...........A......................-...... : 218
DAA*04:01   : ...........A........V.............-...... : 225
DAA*05:01   : ...........A........V.............-...... : 225
DAA*05:02   : ...........A........V.............-...... : 225
DAA*06:01   : ...........A......................-...... : 225
DAA*07:01   : ...........A......................-...... : 225
DAA*08:01   : ...........A......................-...... : 218
DAA*09:01   : ...........A........V.............-...... : 229
DAA*10:01   : ...........A......................-...... : 229
DAA*11:01   : ...........A......................-...... : 218
DAA*12:01   : ...........A........V.............-...... : 218
DAA*13:01   : ...........A......................-...... : 225
DAA*14:01   : ...........A......................-...... : 225
```

SF5B. Alignment of deduced Atlantic salmon MHC class II beta amino acid sequences

Alignment of deduced Atlantic salmon MHC class II beta amino acid sequences. Dots indicates sequence identity, dashes show missing sequence. Amino acids are numbered above the sequence alignment according to the DAB*01:01 sequence. Individual domains are shown above the alignment. Lines without sequence are not shown. Location of primer sequences used to amplify fragments for Illumina sequencing are indicated using green color. CP is connecting peptide, TM is transmembrane region and CYT is cytoplasmic region.

Leader sequence

```
           Forward primer regions
                         *
DAB*01:01  : --MSIFCVSLTLVLSIFSGT- :  18
DAB*01:02  : MS..................- :  20
DAB*12:01  : -------.............- :  13
DAB*02:01  : MS..................- :  20
AS6DAB1s2  : -------.............- :  13
AS7DAB2s1  : ------------........- :   8
DAB*02:02  : -------.............- :  13
DAB*07:01  : MS..................- :  20
AS3DAB1s1  : -------.............- :  13
AS9DAB1s1  : -------.............- :  13
DAB*09:01  : -------.............- :  13
AS1DAB2s1  : ------------........- :   8
AS6DAB1s1  : -------.............- :  13
AS8DAB2s1  : ------------........- :   8
AS10_DAB1  : -------.............- :  13
DAB*09:02  : -------.............- :  13
DAB*15:02  : MS..................- :  20
DAB*17:03  : MS..................- :  20
DAB*26:01  : MS..................- :  20
DAB*28:01  : MS..................- :  20
DAB*05:01  : MS..................- :  20
DAB*08:01  : MS..................- :  20
AS3DAB1s2  : -------.............- :  13
AS8DAB2s2  : ------------........- :   8
DAB*08:02  : -------.............- :  13
DAB*21:01  : MS..................- :  20
DAB*24:01  : MS..................- :  20
DAB*04:01  : MS..................- :  20
DAB*20:01  : MS..................- :  20
AS2DAB1s1  : -------.............- :  13
AS9DAB1s2  : -------.............- :  13
DAB*10:01  : -------.............- :  13
DAB*11:01  : -------.............- :  13
DAB*23:02  : MS..................- :  20
DAB*06:01  : MS..................- :  20
AS1DAB2s2  : ------------........- :   8
AS2DAB1s2  : -------.............- :  13
AS5DAB2s1  : ------------........- :   8
AS7DAB2s2  : ------------........- :   8
DAB*13:03  : MS..................- :  20
DAB*27:01  : MS..................- :  20
DAB*03:01  : MS..................- :  20
DAB*03:03  : MS..................- :  20
```

Beta 1 domain

```
              20         *        40         *        60         *        80         *       100
DAB*01:01  : DGYFEQVVRQCRYSSKDLQGIEFIDSYVFNKAEYIRFNSTVGKFVGYTELGVKNAEAWNSDAAVLAVERGELERYCKHNADLHYSTILDKT : 109
DAB*01:02  : ......................L.................................................................... : 111
DAB*12:01  : ...........................................Y.....Y.........KGPE-..RAL.....V.....PIY..A..... : 103
DAB*12:02  : ...........................................Y.....Y.........KGPE-..RAL........F..PIY..A----- :  85
DAB*02:01  : ..................................V........Y...............KGPE-....L.....F.....AIY..A..... : 110
AS6DAB1s2  : ..................................V........Y...............KGPE-....L.....F.....AIY..A..... : 103
AS7DAB2s1  : ..................................V........Y...............KGPE-....L.....F.....AIY..A..... :  98
DAB*02:02  : .....H............................V........Y...............KGPE-....L.....F.....AIY..A..... : 103
DAB*07:01  : ..................................V........Y...............KGPE-....L.....F.........RA..... : 110
AS3DAB1s1  : ..................................V........Y...............KGPE-....L.....F.........RA..... : 103
AS9DAB1s1  : ..................................V........Y...............KGPE-....L.....F.........RA..... : 103
DAB*09:01  : ..................................V........Y...............KGPE-....L........L..PID..A..... : 103
AS1DAB2s1  : ..................................V........Y...............KGPE-....L........L..PID..A..... :  98
AS6DAB1s1  : ..................................V........Y...............KGPE-....L........L..PID..A..... : 103
AS8DAB2s1  : ..................................V........................KGPE-....L........L..PID..A..... :  98
AS10DAB1s1 : ..................................V........Y...............KGPE-....L........L..PID..A..... : 103
DAB*09:02  : ..................................V........................KGPE-....L........L..PID..A..... : 103
DAB*16:01  : ..............................Q.............................KGPE-....L........L..PID..A----- :  85
DAB*15:02  : ....Y.........................Q..................H.........KGPE-..G.L........L..PID..A..... : 110
DAB*17:01  : ............F.......T.............V........Y...............KGPE-..G.L.....F.....PID..A----- :  85
DAB*17:02  : ........S...F.......T.............V........Y...............KGPE-....L.....F..Q..PID..A----- :  85
DAB*17:03  : ........S...F.......T.............V........Y...............KGPE-....L.....F.....PID..A..... : 110
DAB*25:01  : ........S...F...........H..................Y...............KGPE-..G.L.....F.....PID..A----- :  85
DAB*26:01  : ........S...F...........H..................Y...............KGPE-..G.L.....F................ : 110
DAB*28:01  : ........S...F...........H..................Y...............KGPE-..G.L........L..PID..A..... : 110
DAB*05:01  : ....Y.............................V........Y.....Y.........KGPE-..G.L...........PIY..A..... : 110
DAB*08:01  : ....Y.R.SE........................V........Y.....Y.........KGPE-..G.L.....V.....PID..A..... : 110
AS3DAB1s2  : ....Y.R.SE........................V........Y.....Y.........KGPE-..G.L.....V.....PID..A..... : 103
AS8DAB2s2  : ....Y.R.SE........................V........Y.....Y.........KGPE-..G.L.....V.....PID..A..... :  98
DAB*08:02  : ....Y.R.SE........................V........Y.....Y.........KGPE-..G.L.....V.....PIY..A..... : 103
DAB*21:01  : ....Y.R.SE....................Q..N..........................KGPE-..G.L.....F................ : 110
DAB*14:01  : ....Y.........................Q..................H.........KGPE-....L.................----- :  85
DAB*15:01  : ....Y.........................Q..................H.........KGPE-....L.....F.....PID..A----- :  85
DAB*24:01  : ....F.........................Q..N..........................KGPE-..RAL........L..PIY..A..... : 110
DAB*04:01  : ....F...................H.....Q..N.........Y...............KGPE-..G.L.....F................ : 110
DAB*20:01  : ....F...................H.....Q..N.........Y...............KGPE-....L............ID..A..... : 110
AS2DAB1s1  : ....F...................H.....Q..N.........Y...............KGPE-....L............ID..A..... : 103
AS9DAB1s2  : ....F...................H.....Q..N.........Y...............KGPE-....L............ID..A..... : 103
DAB*10:01  : ....H.R.T.....................Q..........................L.KGPE-....L........L..PID..A..... : 103
DAB*11:01  : ....H.R.T.....................Q..................H.........KGPE-..G.L........F..PIY..A..... : 103
DAB*11:02  : ....H.R.T.....................Q..................H.........KGPE-..G.L.....V.....PIY..A----- :  85
DAB*23:01  : ....H.R.T.....................Q.............................KGPE-..G.L.V..........ID..A----- :  85
DAB*23:02  : ....H.R.T..P..................Q.............................KGPE-..G.L.V..........ID..A..... : 110
DAB*18:01  : ....H.R.T.....................Q..................H.............G..G.L.....F..........------ :  85
DAB*06:01  : ....YHMMT.............L.T.....Q..N...............H.........KGPE-..G.L.V......F..PID..A..... : 110
AS1DAB2s2  : ....YHMMT.............L.T.....Q..N...............H.........KGPE-..G.L.V......F..PID..A..... :  98
AS2DAB1s2  : ....YHMMT.............L.T.....Q..N...............H.........KGPE-..G.L.V......F..PID..A..... : 103
AS5DAB2s1  : ....YHMMT.............L.T.....Q..N...............H.........KGPE-..G.L.V......F..PID..A..... :  98
AS7DAB2s2  : ....YHMMT.............L.T.....Q..N...............H.........KGPE-..G.L.V......F..PID..A..... :  98
DAB*13:03  : ....YHMM..............L.T.....Q..N...............H.........KGPE-..G.L.....V................ : 110
DAB*13:02  : ....YHMM..............L.T.....Q..N...............H.........KGPE-..G.L.....F...........----- :  85
DAB*22:01  : ....YHMM..............L.T.....Q..N...............H.........KGPE-..G.L.....F.....PID..A----- :  85
AS5DAB2s2  : ....YHMM..............L.T.....Q..N...............H.........KGPE-..G.L.....V.....PIY..A..... :  98
DAB*27:01  : ....YHMM..............L.T.....Q..N..........................KGPE-..G.L.V......L..PIY........ : 110
DAB*13:01  : ....YHMM..............L.T.....Q..N..........................KGPE-..G.L.V...F...........----- :  85
DAB*03:01  : ....YHMM..............L.T.....Q............Y.....Y.........KGPE-..G.L.....V.....PID..A..... : 110
DAB*03:02  : ....YHMM..............L.T.....Q............Y.....Y.........KGPE-..G.L.....V.....PIY..A----- :  85
DAB*03:03  : ....YHMM..............L.T.....Q............Y.....Y.........KGPE-..G.L.V..........PIY..A..... : 110
DAB*19:01  : ....YHMM..............L.T.....Q............Y.....Y.........KGPE-..G.L...........AN...A----- :  85
```

Beta 2 domain

```
                                                                  Illumina reverse primer
                       120         *       140         *       160         *       180         *       200
DAB*01:01  : VEPHVRLSSVAPPSGRHPAMLMCSAYDFYPKPIRVTWLRDGREVKSDVTSTEELANGDWYQIHSHLEYTPRSGEKISCMVEHISLTEPMVYHW : 203
DAB*01:02  : ............................................................................................. : 205
DAB*12:01  : ............................................................................................. : 197
DAB*02:01  : ............................................................................................. : 204
AS6DAB1s2  : .....................................................---------------------------------------- : 156
AS7DAB2s1  : .....................................................---------------------------------------- : 151
DAB*02:02  : ............................................................................................. : 197
DAB*07:01  : ............................................................................................. : 204
AS3DAB1s1  : .....................................................---------------------------------------- : 156
AS9DAB1s1  : .....................................................---------------------------------------- : 156
DAB*09:01  : ............................................................................................. : 197
AS1DAB2s1  : .....................................................---------------------------------------- : 151
AS6DAB1s1  : .....................................................---------------------------------------- : 156
AS8DAB2s1  : .....................................................---------------------------------------- : 151
AS10DAB1s1 : .....................................................---------------------------------------- : 156
DAB*09:02  : ............................................................................................. : 197
DAB*15:02  : ............................................................................................. : 204
DAB*17:03  : ............................................................................................. : 204
DAB*26:01  : ............................................................................................. : 204
DAB*28:01  : ............................................................................................. : 204
DAB*05:01  : .....................I....................................................................... : 204
DAB*08:01  : ............................F................................................................ : 204
AS3DAB1s2  : ............................F........................---------------------------------------- : 156
AS8DAB2s2  : ............................F........................---------------------------------------- : 151
DAB*08:02  : ............................F................................................................ : 197
DAB*21:01  : ............................................................................................. : 204
DAB*24:01  : ............................................................................................. : 204
DAB*04:01  : ............................................................................................. : 204
DAB*20:01  : ............................................................................................. : 204
AS2DAB1s1  : .....................................................---------------------------------------- : 156
AS9DAB1s2  : .....................................................---------------------------------------- : 156
DAB*10:01  : ............................................................................................. : 197
DAB*11:01  : ............................................................................................. : 197
DAB*23:02  : ............................................................................................. : 204
DAB*06:01  : ............................F................................................................ : 204
AS1DAB2s2  : ............................F........................---------------------------------------- : 151
AS2DAB1s2  : ............................F........................---------------------------------------- : 156
AS5DAB2s1  : ............................F........................---------------------------------------- : 151
AS7DAB2s2  : ............................F........................---------------------------------------- : 151
DAB*13:03  : ............................F................................................................ : 204
AS5DAB2s2  : ............................F........................---------------------------------------- : 151
DAB*27:01  : ............................F................................................................ : 204
DAB*03:01  : ............................F................................................................ : 204
DAB*03:03  : ............................F................................................................ : 204
```

CP/ TM/ CYT domains

```
                     *       220         *       240
DAB*01:01  : DPSLPEAERNKIAIGASGLVLGAILALAGLIYYKKKSSGVL : 244
DAB*01:02  : ...................---------------------- : 224
DAB*12:01  : ............----------------------------- : 209
DAB*02:01  : ...................---------------------- : 223
DAB*02:02  : ............----------------------------- : 209
DAB*07:01  : ......................................... : 245
DAB*09:01  : ............----------------------------- : 209
DAB*09:02  : ............----------------------------- : 209
DAB*15:02  : ...................---------------------- : 223
DAB*17:03  : ...................---------------------- : 223
DAB*26:01  : ...................---------------------- : 223
DAB*28:01  : ...................---------------------- : 223
DAB*05:01  : ...................---------------------- : 223
DAB*08:01  : ...................---------------------- : 223
DAB*08:02  : ............----------------------------- : 209
DAB*21:01  : ...................---------------------- : 223
DAB*24:01  : ...................---------------------- : 223
DAB*04:01  : ...................---------------------- : 223
DAB*20:01  : ...................---------------------- : 223
DAB*10:01  : ............----------------------------- : 209
DAB*11:01  : ............----------------------------- : 209
DAB*23:02  : ...................---------------------- : 223
DAB*06:01  : ......................................... : 245
DAB*13:03  : ...................---------------------- : 223
DAB*27:01  : ...................---------------------- : 223
DAB*03:01  : ...................---------------------- : 223
DAB*03:03  : ...................---------------------- : 223
```

SF5C. Alignment of deduced Atlantic salmon MHC class I amino acid sequences

Alignment of deduced Atlantic salmon MHC class I amino acid sequences. Dots indicates sequence identity, dashes show missing sequence. Amino acids are numbered above the sequence alignment according to the UBA*01:01 sequence. Primer regions and individual domains are shown above the alignment. See Table 1 and supplementary file 1 for location of individual forward primers. The seven alpha 1 domain lineages (Kiruy et al.2005) are shown on the right hand side of the alpha 1 domain alignment. Lines without sequence are not shown. Location of primer sequences used to amplify fragments are indicated using green color. CP is connecting peptide, TM is transmembrane region and CYT is cytoplasmic region. Accession numbers for the non-UBA sequences are as follows: UGA is ACX35601.1, UDA is ACY30371.1 and ULA is ABQ59666.1.

```
Leader sequence
                           Illumina Forward primers
                            *
UBA*01:01  : ------MKGFILLVLGIGLLHTASA :  19
AS10UBA1s1 : ---------------------.... :   4
AS2UBA1s1  : ---------------------.... :   4
UBA*34:02  : --------------........... :  11
UBA*05:01  : ---------................ :  16
UBA*35:01  : -------------------...... :   6
AS2UBA1s2  : ---------------------.... :   4
AS7UBA1s1  : ---------------------.... :   4
AS3UBA1s2  : ---------------------.... :   4
AS6UBA1s1  : ---------------------.... :   4
AS9UBA1s1  : ---------------------.... :   4
AS8UBA1s1  : ---------------------.... :   4
AS9UBA1s2  : ---------------------.... :   4
UBA*37:01  : --------------........... :  11
UBA*11:01  : --------------........... :  11
UBA*04:01  : -------------I........... :  12
UBA*03:01  : -------MKCFI.L.LGIA..SS.. :  18
UBA*03:02  : ---------CFI.L.LGIA..SS.. :  16
UBA*36:01  : ---------CFI.L.LGIA..SS.. :  16
UBA*38:01  : ---------CFI.L.LGIA..SS.. :  16
UBA*08:01  : ---------................ :  16
UBA*02:01  : ---------........V....V.. :  16
AS6UBA1s2  : ---------------------.V.. :   4
AS7UBA1s2  : ---------------------.V.. :   4
UBA*06:01  : ---------................ :  16
AS1UBA1s1  : ---------------------.... :   4
AS3UBA1s1  : ---------------------.... :   4
AS8UBA1s2  : ---------------------.... :   4
UBA*06:03  : --------------........... :  11
UBA*07:01  : ---------................ :  16
UBA*14:01  : ------................... :  19
UBA*15:01  : ---------------.......T.. :  10
UBA*33:01  : ------------FIILLLGINA... :  13
UBA*09:01  : ----------............... :  15
UBA*10:01  : -------------------..SS.. :   6
UDA        : ------......MFM.TCH.FE.FG :  19
ULA        : -------MKCFI.L.LSIS..A... :  18
UGA        : MKTRLISAMK.YF..-LSCI.G.LS :  24
```

Alpha 1 domain

```
              Illumina forward primer
              20        *         40        *         60        *         80        *         100
UBA*01:01   : VTHALKYFYTASSEVPNFPEFVVVGVVDGVQMVHYDSNSQRAVPKQDWVNKAAD--PQYWERNTGIFKGSQQTFKANIDIAKQRFNQSG-- : 106  I
UBA*34:01   : ......................................................--.................................-- :  87
AS10UBA1s1  : ......................................................--.................................-- :  91
UBA*34:02   : ........................AM............................--........................V........-- :  98
AS2UBA1s1   : ........................AM............................--........................V........-- :  91
UBA*05:01   : ......................................................--.................................-- : 103
UBA*18:01   : ......................................................--.................................-- :  87
UBA*18:02   : ......................................................--.................................-- :  87
UBA*35:01   : ......................................................--.........NC.....I.......V........-- :  93
AS2UBA1s2   : ......................................................--.........NC.....I.......V........-- :  91
AS7UBA1s1   : ......................A.........F.....................--.........NC.....I.......V........-- :  91
UBA*29:01   : ...T..................A...............C...............--.........NC.....I.......V........-- :  87
UBA*19:01   : ...................G..A...............................--.........N.......................-- :  87
UBA*27:01   : ......................A.........F.....................--.........NC.....I.......V........-- :  87
UBA*13:01   : ...S....................SM......................M....EAL....DIE..K.L..H.S.......C........-- :  89
AS3UBA1s2   : ...S....................SM......................M....EAL....DIE..K.L..H.S.......C........-- :  93
AS6UBA1s1   : ...S....................SM......................M....EAL....DIE..K.L..H.S.......C........-- :  93
AS9UBA1s1   : ...S....................SM......................M....EAL....DIE..K.L..H.S.......C........-- :  93
AS8UBA1s1   : ...S....................SM......................M....EAL....DIE..K.L..H.S.......C........-- :  93
UBA*37:01   : ...S....................SM......................M....EAL....DIE..K.L..H.S.......C........-- : 100
UBA*21:01   : ...S....................SM......................M....EAL....DIE..K.L..H.S.......C........-- :  89
UBA*11:01   : ...S.G..................SM......................M....EAL....DIE..K.L..H.S.......C........-- : 100
UBA*04:01   : ...S....................SM......................M....EAL....DIE..K.L..H.S.......C........-- : 101
UBA*04:02   : ...S....................SM.....................CI....EAL....DIE..K.L..H.S.......C........-- :  89
UBA*28:01   : ...S....................SM......................M....EAL....DIE..K.L..H.S.......C........-- :  89
UBA*22:01   : ......................A.........................M....EAL....DIE..NLL..H.S.......C........-- :  89
AS9UBA1s2   : ......................A.........................M....EAL....DIE..NLL..H.S.......C........-- :  93
UBA*03:01   : A..S.R.V...T.GI.D.....T..L.N.EPISY...IIR.ET.R...MA.TEG--SD...SQ.QVSI..E........V.......T.-- : 105  III
UBA*03:02   : A..S.R.V...T.GI.D.....T..L.N.EPISY...IIR.ET.R...MA.TEG--SD...SQ.QVSI..E........V.......T.-- : 103
UBA*36:01   : A..S.R.V...T.GI.D.....T..L.N.EPISY...IIR.ET.R...MA.TEG--SD...SQ.QVSI..E........V.......T.-- : 103
UBA*38:01   : A..S.R.V...T.GI.D.....T..L.N.EPISY...IIR.ET.R...MA.TEG--SD...SQ.QVSI..E........V.......T.-- : 103
UBA*12:01   : --.S.R.V...T.GI.D.....T..L.N.EPISY...IIR.ET.R...MA.TEG--SD...SQ.QVSI..E........V.......T.-- :  85
UBA*25:01   : A..S.R.V...T.GI.D.....NL.I...M.IDY....TK........MA.TEG--SD..D.Q.QVSI..E.........V......T.-- :  87
UBA*32:01   : A..S.R.V...T.GI.D.....NL.I...M.IDY....TK........MA.TEG--SD..D.Q.QVSI..E.........V......T.-- :  87
UBA*30:01   : A..S.R.V...T.GI.D.....NL.I...M.IDY....TK........MA.TEG--SD.RD.Q.QVSI..E.........V......T.-- :  87
UBA*31:01   : A..S.R.V...T.GI.D.....NL.I...M.IDY....TK........MA.TEG--SD..D.Q.QVSI..E.........V......T.-- :  87
UBA*08:01   : A..S.R.V...T.GI.D.....NL.I...M.IDY....TK........MA.TEG--SD..D.Q.QVSI..E.........V......T.-- : 103
UBA*20:01   : A.NT.Q.....T.GID......TM.I.N.H.ID....ITK..IQ.AE.ISG.V.--.D..KT..Q.YA.TETV.VN..NV..S....T.-- :  87 VI
AS7UBA1s2   : A.NT.Q.....T.GID......TM.I.N.H.ID....ITK..IQ.AE.ISG.V.--.D..KT..Q.YA.TETV.VN..NV..S....T.-- :  91
UBA*20:02   : --NT.Q.....T.GID......TM.I.N.H.ID....ITK..IQ.AE.ISG.V.--.DI.KT..Q.YA.TETV.VN..NV..S....T.-- :  85
UBA*24:01   : A.NT.Q.....T.GIG......TM.I.N.H.ID....ITK..IQ.AE.ISG.V.--.D..KT..Q.YA.TETV.VN..NV..S....T.-- :  87
UBA*24:02   : A.NT.Q.....T.GID......TM.I.N.H.ID....ITK..IQ.AE.ISG.V.--.D..KT..Q.YA.TETV.VN..NV..S....T.-- :  87
UBA*24:03   : A.NT.Q.....T.GID......TM.I.N.H.ID....ITK..IQ.AE.ISG.V.--.D..KT..Q.YA.TETV.VN..NV..S....T.-- :  87
UBA*23:01   : A.NT.Q.....T.GID......TM.I.N.H.ID....ITK..IQ.AE.ISG.V.--.D..KT..Q.YA.TETV.VN..NV..S....T.-- :  87
UBA*02:01   : A.NT.Q.....T.GID......TM.I.N.H.ID....ITK..IQ.AE.ISG.V.--.D..KT..Q.YA.TETV.VN..NV..S....T.-- : 103
AS6UBA1s2   : A.NT.Q.....T.GID......TM.I.N.H.ID....ITK..IQ.AE.ISG.V.--.D..KT..Q.YA.TETV.VN..NV..S....T.-- :  91
UBA*06:01   : A..S.......V.GDID....TI..L.NNG.F.Y....IK.M...TE.MKQS.G--AD..DTESEKQV.QN.G..N..QVL.D.....MST : 105 V
AS8UBA1s2   : A..S.......V.GDID....TI..L.NNG.F.Y....IK.M...TE.MKQS.G--AD..DTESEKQV.QN.G..N..QVL.D.....MST :  93
UBA*06:02   : A..S.......V.GDID....TI..L.NNG.F.Y....IK.M...TE.MKQS.G--AD..DTESEKQV.QN.G..N..QVP.D.....MST :  89
UBA*06:03   : A..S.......V.GDID....TI..L.NNG.F.Y....IK.M...TE.MKQS.G--AD..DTESEKQV.QN.G..N..QVL.D.....MST : 100
UBA*07:01   : A..S.......V.GDID....TI..L.NNG.F.Y....IK.M...TE.MKQS.G--AD..DTESEKQV.QN.G..N..QVL.D.....MST : 105
AS1UBA1s1   : A..S.......V.GDID....TI..L.NNG.F.Y....IK.M...TE.MKQS.G--AD..DTESEKQV.QN.G..N..QVL.D.....MST :  93
AS3UBA1s1   : A..S.......V.GDID....TI..L.NNG.F.Y....IK.M...TE.MKQS.G--AD..DTESEKQV.QN.G..N..QVL.D.....MST :  93
UBA*26:01   : A..S.......V.GDID....T...L..EG.FMYF...TKT....TE.MK.SVG--AD..D.Q.Q.GI.AH.N.....QV..D.....KST :  89
UBA*26:02   : A..S.......V.GDID....T...L..EG.FMYF...TKT....TE.MK.SVG--AD..D.Q.Q.GI.AH.N.....QV..D.....KST :  89
UBA*16:01   : A..S.......V.GDID....T...L..EG.FMYF...TKT....TE.MK.SVG--AD..D.Q.Q.GI.AH.N.....QV..D.....KST :  89
UBA*17:01   : AA.S.......V.GDID....T...L..EG.FMYF...TKT....TE.MK.SVG--AD..D.Q.Q.GI.AH.N.....QV..D.....KST :  89
UBA*14:01   : A..S.......V.GDID....T...L..EG.FMYF...TKT....TE.MK.SVG--AD..D.Q.Q.GI.AH.N.....QV..D.....KST : 108
UBA*15:01   : AI.T..N....A.GDIG....II..L..NTPYLYF...TKT....TE.MK.SVG--AD..DSM.Q.GI.AN.I.....QVV.D.....KST :  99
UBA*33:01   : ...S......G.TGIEG..Q..A..I...MHIDYF..V.EKN.L..S.MEG.R.-----EKSI.N.R..H..S....VE.VM.....TT-- :  97 IV
AS5UBA2s1   : ----......G.TGIEG..Q..A..I...MHIDYF..V.EKN.L..S.MEG.R.-----EKSI.N.R..N..S....VE.VM.....TT-- :  80
AS5UBA2s2   : ----......G.TGIEG..Q..A..I...MHIDYF..V.EKN.L..S.MEG.R.-----EKSI.N.R..N..S....VE.VM.....TT-- :  80
UBA*09:01   : ...S.R.....TTGI.D.....D....N.KVISY...IIK.K....S.MEENLN--Q...NQG.DQL..TE.S.....QV.QT....T.-- : 102 II
UBA*10:01   : .I.SW.A.L...TGLSD.....ALNL..DEL.GYF.TKTN.FEG..S..EEKLG--Q..L.QQEN.LRSTS.S..V.VG..ME....TK-- :  93 VII
UDA         : ...S..H......K.T.....M...M.....ID.....I..M......M..QTEAE--....E...AFD...V..DDVN.L........-- : 106
ULA         : AM.S.R.V...T.GM.D....MT..L.N.EPISY...IIR.ET.R...MKE.V.--.D..N...QTSI.DE........V.......T.-- : 105
UGA         : .I.S.R....S..GISD.....DM.M.NDQVIS....ITK.K....S.MG.VF.--Q...DST.EDLR.AEKV..N.LQT.QK....T.-- : 111
```

Alpha 2 domain

```
                                                                                    Illumina reverse primer
                  *        120         *        140         *        160         *        180         *
UBA*01:01   : GVHVNQWMYGCEWDDEAGVTEGFEQWGYDGEDFIAFDLKTKSWIAPTPQSVITKLKWDSDTAQNEHDKHYLTQTCIEWLKKYLDYGKSTLMRT : 199
UBA*34:01   : .................................................A................R.N.Y..I........V.--------- : 171
AS10UBA1s1  : .................................................A................R.N.Y..I...---------------- : 168
UBA*34:02   : .................................................A................R.N.Y..I........V.......... : 191
AS2UBA1s1   : ...IY.N................D.Y.......L......LK........L.......NNM..IQQ...........---------------- : 168 new
UBA*05:01   : ......R.........T......N.D..............LK.......A........NNM..IQQ........................... : 196
UBA*18:01   : ....F.M.C.........A....D.Y.......................A.........N.....YW.N....E........V.........- : 179
UBA*18:02   : ....F.M.C.........T....D.Y.......................A....I....N..H..CW.N....E........V.........- : 179
UBA*35:01   : ....F.N................D.Y...............T....KT.A.N..N..........FL.N.Y...................... : 186
AS2UBA1s2   : ......N................D.Y.......L......LK........L.......NNM..IQQ...........---------------- : 168 new
AS7UBA1s1   : ....F.N................D.Y...............T....KT.A.N..N..........FL.N.Y......---------------- : 168 new
UBA*29:01   : ....F.M.C.........A....D.Y.........L.............A.........N.....YW.N....E........V.........- : 179
UBA*19:01   : ....F.ICM.......T.A....N.D.......L......LK.........N..H....N..Y...W.N....E........V.........- : 179
UBA*27:01   : ................T......N.D.......................A...............YL.N....E..........--------- : 171
UBA*13:01   : ...IV.K.........T......N.Y..............LK.......A...............YR.N.Y...........V.--------- : 173
AS3UBA1s2   : ...IV.K.........T......N.Y..............LK.......A...............YR.N.Y......---------------- : 170
AS6UBA1s1   : ...IV.K.........T......N.Y..............LK.......A...............YR.N.Y......---------------- : 170
AS9UBA1s1   : ...IV.K.........T......N.Y..............LK.......A...............YR.N.Y......---------------- : 170
AS8UBA1s1   : ...IV.K.........T......N.Y..............LK.......A...............YR.N.Y......---------------- : 170
UBA*37:01   : ................T......N.D.......................A...............YL.N....E................... : 193
UBA*21:01   : .................................................A................R.N.Y..I........V.........- : 181
UBA*11:01   : ...IV.M.C.......T......N.D.......................A.........N.....YW.N....E........V.......... : 193
UBA*04:01   : ....F.K...........A...LT.Y...............--.......L...........Y..QE.N....I........V.......... : 192
UBA*04:02   : ....F.K...........A...LT.Y........................L...........Y..QE.N....I........V.......... : 182
UBA*28:01   : ...IF.Y....T...DS...D.LR.Y.......LVY.M.AFT....KL.AE..TR..NNEP..M.YL.S.I..E.V......V.--------- : 173
UBA*22:01   : ...IV.R.C.......T......N.D.......................A.........N.....YW.N....E........V.........- : 181
AS9UBA1s2   : .................................................A................R.N.Y..I...---------------- : 170 new
UBA*03:01   : ......K.........T......D.D.......L......LT.......A.........N.....YR.N........................ : 198
UBA*03:02   : ......K...S.....T......D.D.......L......LT.......A.........N.....YR.N................E....... : 196
UBA*36:01   : ....F.RT.........................................A............Y..QE.N....I........V.......... : 196
UBA*38:01   : .................................................A................R.N.Y..I........V.......... : 196
UBA*12:01   : ....F.M.C.........A....D.Y.......................A.........N.....YW.N....E........V.......... : 178
UBA*25:01   : .................................................A................R.N.Y..I...R....V.........- : 179
UBA*32:01   : ..............................K..M...M...........A................RED.Y..M........V.........- : 179
UBA*30:01   : ..................A..W...........................A.........G..H..YR.N.YSHI.T..M...VE.....V..- : 179
UBA*31:01   : .........................Y.......................A.........N.....YW.N....E........V.........- : 179
UBA*08:01   : ......N................D.Y.......L......LK........L.......NNM..IQQ........................... : 196
UBA*20:01   : ....F.M.C.........A....D.Y.......................A.........N.....YW.N....E........V.--------- : 171
AS7UBA1s2   : ....F.M.C.........A....D.Y.......................A.........N.....YW.N....E...---------------- : 168
UBA*20:02   : ....F.M.C.........A....D.Y.......................A.........N.....YW.N....E........V.......... : 178
UBA*24:01   : ...IV.R.C.......T......N.D.......................A.........N.....YW.N....E........V.........- : 179
UBA*24:02   : ...IV.R.C.......T......N.D.......................A.........N.....YW.N....E........V.--------- : 171
UBA*24:03   : ...IV.R.C.......T......N.N........Q..............A.........N.....YW.N....E........VG........- : 179
UBA*23:01   : ..P...K.........T......N.D..............LK.......A........NNM..IQQ..........................- : 179
UBA*02:01   : ......K.........T......D.D.......L......LT.......A.........N.....YR.N........................ : 196
AS6UBA1s2   : ......K.........T......D.D.......L......LT.......A.........N.....YR.N........---------------- : 168
UBA*06:01   : .................................................A................R.N.Y..I........V.......... : 198
AS8UBA1s2   : .................................................A................R.N.Y..I...---------------- : 170
UBA*06:02   : .................................................A................R.N.Y..I........V.......... : 182
UBA*06:03   : ....S.......................................V....A................R.NHY..I........V.......... : 193
UBA*07:01   : ....F.V...........A....D.Y.......L......LK.......A...............YR.N.Y...........V.......... : 198
AS1UBA1s1   : ....F.V...........A....D.Y.......L......LK.......A...............YR.N.Y......---------------- : 170
AS3UBA1s1   : ....F.V...........A....D.Y.......L......LK.......A...............YR.N.Y......---------------- : 170
UBA*26:01   : ....F.N.........T......D.D.......L......LT.......A.N..H....N..Y..QR.N....I........V.........- : 181
UBA*26:02   : ....F.N.........T......DHD.......L......LT.......A.N..H....N..Y..QE.N....I....M...V.........- : 181
UBA*16:01   : ......N................D.Y.......L......LK........L.......NNM..IQQ..........................- : 181
UBA*17:01   : .................................................A................R.N.Y..I........V.........- : 181
UBA*14:01   : ....F.K...........A...LT.Y........................L...........Y..QE.N....I........V.......... : 201
UBA*15:01   : ....F.N................D.Y....G.......E..T....KT.A.N..N..........FL.N.Y...................... : 192
UBA*33:01   : ....F.K...........A...LT.Y........................L...........Y..QE.N....I........V.......... : 190
AS5UBA2s1   : ....F.N.........T......D.D.......L......LT.......A.N..H....N..Y..QE.N....I...---------------- : 157 new
AS5UBA2s2   : .................................................A................R.N.Y..I...---------------- : 157 new
UBA*09:01   : ...IF.Y....T...DS...D.LR.Y.......LVY.M.AFT....KL.AE..TR..NNEP..M.YL.S.I..E.V......V....N..... : 195
UBA*10:01   : ....F.N.........T......D.D.......L......LT.......A.N..H....N..Y..QE.N....I........V.......... : 186
UDA         : ....L.YI...S....TEQRD..G.L..N....LVY.MN.LT.K.LKQ.ADVMRD..NR.ISRLVFW.T.FS.....C...QVVN....-L.. : 198
ULA         : ....Y.N................D.Y.......L......LK........L.......NNM..IQQ........................... : 198
UGA         : .M.IS.D.........T.L....HHI....Q.LLV....RAT...SV..ALHS.M..EG.PSSI.SE.R....D..V......E...T..Q.. : 204
```

Alpha 3 domain

```
               200        *        220        *        240        *        260        *        280        *
UBA*01:01  : VPPSVSLLQKTPSSPVTCHATGFYPSGVMVSWQKDGQDHHEDVEYGETLQNDDGTFQKSSHLTVTPEEWKNNKYQCVVQVTGVKEDFIKVLT : 291
UBA*34:02  : ............................................................................................ : 283
UBA*05:01  : ............................................H.....................................LQ........ : 288
UBA*35:01  : ..............................................................................H............. : 278
UBA*37:01  : .................................................P.........I.......DR...........K.I......... : 285
UBA*11:01  : ............................................H.....................................I.D.I..... : 285
UBA*04:01  : ............................................................................................ : 284
UBA*03:01  : ............................................H.....................................LQ........ : 290
UBA*03:02  : ............................................H.....................................LQ........ : 288
UBA*36:01  : ............................................................................................ : 288
UBA*38:01  : ............................................................................................ : 288
UBA*12:01  : ............................................H.....................................I.D.I..... : 270
UBA*08:01  : ............................................H.....................................LQ........ : 288
UBA*20:02  : ............................................H.....................................I.D.I..... : 270
UBA*02:01  : ............................................H.....................................LQ........ : 288
UBA*06:01  : ............................................................................................ : 290
UBA*06:03  : ............................................................................................ : 285
UBA*07:01  : ............................................H.....................................I.D.I..... : 290
UBA*14:01  : ............................................................................................ : 293
UBA*15:01  : ............................................H...................................K.LQ........ : 284
UBA*33:01  : ............................................................................................ : 282
UBA*09:01  : ..........S...................F.......Q..........P.H...............DR.............I.D....... : 287
UBA*10:01  : ..................................................................................LQ........ : 278
UDA        : A.............................F......EQ.....H..I.H.........T..R...................I......... : 290
ULA        : ............................................................................................ : 290
UGA        : ..............................F.............N....H........RT..K..S................I......... : 296
```

CP/ TM/ CYT domains

```
                     300        *        320         *         340       *
UBA*01:01  : ESEIKTNWNEPNIVLIIVVVVALLLLVVA-VVVGVVIWK-KKSKKGFVPASTSDTDSDNSGRAAQMT : 356
UBA*34:02  : .........D...................-.........-........................L.. : 348
UBA*05:01  : .........D...................-.........-........................... : 353
UBA*35:01  : .........D..............P....-.........-..................H.------- : 336
UBA*37:01  : D------LDD.......G.......V...-.------------------------------------ : 309
UBA*11:01  : .........D.......G...........-A.A......-........................... : 350
UBA*04:01  : .........D...................-.........-........................... : 349
UBA*03:01  : .........D.......G.......V...-.........-........................... : 355
UBA*03:02  : .........D.......G..E....V...-.........-.-------------------------- : 327
UBA*36:01  : .........D...................-.........-.-------------------------- : 327
UBA*38:01  : .........D...................-.........-.-------------------------- : 327
UBA*12:01  : .........D.......G...........-A.A......-........................... : 335
UBA*08:01  : .........D...................-.........-........................... : 353
UBA*20:02  : .........D.......G...........-A.A......-........................... : 335
UBA*02:01  : .........D.......G.......V...-.........-........................... : 353
UBA*06:01  : .........D...................-.........-........................... : 355
UBA*06:03  : .........D...................-.........-........................... : 350
UBA*07:01  : .........D.......G...........-A.A......-........................... : 355
UBA*14:01  : .........D...................-.........-........................... : 358
UBA*15:01  : .........D...................-.........-..........................A : 349
UBA*33:01  : .........D...................-.........-........................... : 347
UBA*09:01  : DLDDP.----....P..........V...-.........-.................E...KG..KI : 348
UBA*10:01  : .........D...................-.........-........................L.. : 343
UDA        : ....Q...GD.APIIVPIIGGVVA..L.V-.........-............N....VY..KDLLK. : 355
ULA        : ........GNT..--------------------------------.....N...VG.NS.HNTVPKE : 325
UGA        : ....Q..RGVNT.GSAPIIG.VVA....VV....L.M.RR.................E...KG..KI : 363
```
